# Nanopatterns of molecular spoked wheels as giant homologues of benzene tricarboxylic acids†‡

**DOI:** 10.1039/d1sc01381e

**Published:** 2021-06-09

**Authors:** Tristan J. Keller, Christopher Sterzenbach, Joshua Bahr, Taria L. Schneiders, Markus Bursch, Julia Kohn, Theresa Eder, John M. Lupton, Stefan Grimme, Sigurd Höger, Stefan-S. Jester

**Affiliations:** Kekulé-Institut für Organische Chemie und Biochemie, Rheinische Friedrich-Wilhelms-Universität Bonn Gerhard-Domagk-Str. 1 53121 Bonn Germany hoeger@uni-bonn.de stefan.jester@uni-bonn.de; Mulliken Center for Theoretical Chemistry, Rheinische Friedrich-Wilhelms-Universität Bonn Bering str. 4 53115 Bonn Germany grimme@thch.uni-bonn.de; Institut für Experimentelle und Angewandte Physik, Universität Regensburg Universitätsstr. 31 93053 Regensburg Germany john.lupton@physik.uni-regensburg.de

## Abstract

Molecular spoked wheels with *D*_3h_ and *C*_s_ symmetry are synthesized by Vollhardt trimerization of *C*_2v_-symmetric dumbbell structures with central acetylene units and subsequent intramolecular ring closure. Scanning tunneling microscopy of the *D*_3h_-symmetric species at the solid/liquid interface on graphite reveals triporous chiral honeycomb nanopatterns in which the alkoxy side chains dominate the packing over the carboxylic acid groups, which remain unpaired. In contrast, *C*_s_-symmetric isomers partially allow for pairing of the carboxylic acids, which therefore act as a probe for the reduced alkoxy chain nanopattern stabilization. This observation also reflects the adsorbate substrate symmetry mismatch, which leads to an increase of nanopattern complexity and unexpected templating of alkoxy side chains along the graphite armchair directions. State-of-the-art GFN-FF calculations confirm the overall structure of this packing and attribute the unusual side-chain orientation to a steric constraint in a confined environment. These calculations go far beyond conventional simple space-filling models and are therefore particularly suitable for this special case of molecular packing.

## Abstract 2

Scanning tunneling microscopy investigations of phenylene-based molecular spoked wheels with *D*_3h_ and *C*_s_ symmetries on graphite show the competitive or complementary effects of carboxylic acid groups and alkoxy chains on the nanopattern formation.

## Graphical abstract

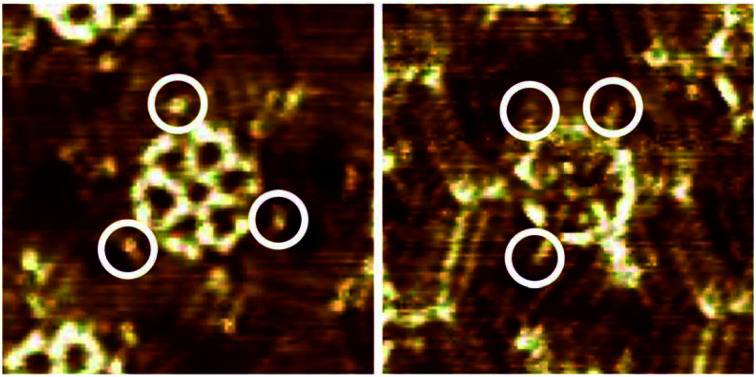

## Introduction

Carbon-based structures composed of arylene, arylene–ethynylene, and arylene–butadiynylene units have become prominent target structures in basic research with broad applications in materials as well as medical chemistry,^1–4^ and several outstanding properties of such compounds are manifest.^5^ Moreover, they have been repeatedly highlighted for their ability to form defined two-dimensionally (2D) crystalline self-assembled monolayers (SAMs) on highly oriented pyrolytic graphite (HOPG) at the solid/liquid interface.^6^ However, the limited shape-persistence^7^ of structures composed of single *p*-phenylene-ethynylene, *p*-phenylene-butadiynylene, and *p*-phenylene units also limits the order of supramolecular nanopatterns.^7*c*,8^ Moreover, *o*- and *m*-phenylene units add rotational degrees of freedom to the otherwise linear segments, sometimes leading to polydispersity regarding conformers.^9^ Such limits can be overcome in strutted architectures, and molecular spoked wheels (MSWs) are prominent examples thereof.^7*b*,10^ The lateral fixation of (smaller) molecular rigid backbones on HOPG can be mediated by a variety of functional groups, such as carboxylic acids as, *e.g.*, in trimesic acid (benzene 1,3,5-tricarboxylic acid)^11*a*,*b*^ and its larger homologues.^11*c*,*d*^ On the other hand, alkyl/alkoxy side chains additionally show a high affinity to graphite,^12^ have an energetically favorable tendency for interdigitating and additionally lead to sufficient compound solubility in common organic solvents. They adsorb along the HOPG main axis (*i.e.* zig–zag) directions,^13^ and only few exceptions are reported for rotated domains in constrained environments, which are then oriented along the armchair direction, whereas other random orientations have not been observed.^14^ Disk-shaped molecular backbones of a certain size and shape generally require an adequate alkyl/alkoxy proportion in order to keep them soluble, and 3,4,5-trihexadecyloxybenzyloxy groups have been proven as successful substituents for this task as well as for the physisorption of shape-persistent macrocycles (SPMs) and MSWs on HOPG.^10*g*^ In the latter case, the formation of *D*_6h_-symmetric structures is a direct consequence of the synthetic strategies in which either six equal spoke-rim-segments are coupled to a central (arylene) hub (*via* Sonogashira reaction),^10*a*,*b*,*d*,*f*^ or symmetrical acetylenes are trimerized (*via* Vollhardt cyclization)^10*g*,15^ before the rim is closed. While efforts have been made to study how the symmetry of 2D supramolecular tectons is related to the nanopatterns formed,^16,17^ reducing the symmetry of the MSWs has not yet been described. Vollhardt acetylene trimerization of an unsymmetrical acetylene is a straightforward method to obtain such compounds.

**Scheme 1:**
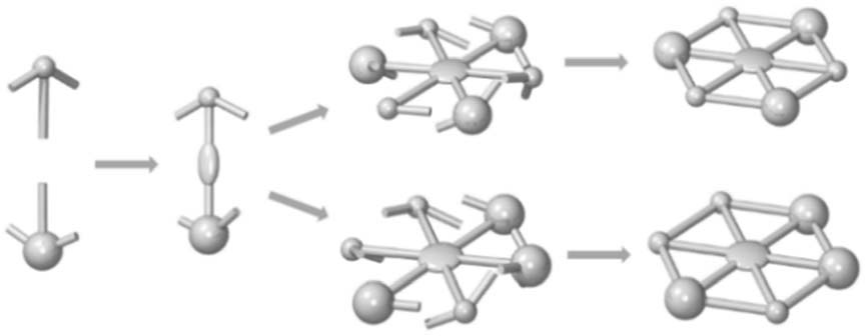
Schematic synthesis of molecular spoked wheels (MSWs) with reduced symmetry *via* Vollhardt trimerization of asymmetrically substituted acetylenes.

When one of the acetylene ends contains the aforementioned 3,4,5-trihexadecyloxybenzyloxy unit (bulky corner in Scheme 1), the processability of the MSWs is maintained even when the remaining corner positions of the final product are widely varied. In case of carboxylic groups, giant size homologues of trimesic acid^11*a*,*b*^ and also trimellitic acid (benzene 1,2,4-tricarboxylic acid)^18^ are available and allow a comparison between the packing behavior of the respective esters and acids. The latter generally dimerize intermolecularly at the surface and stabilize the adsorbate packing. However, it is not predictable if the bulky flexible side groups allow this stabilization also in the present case or if instead the alkyl packing dominates the pattern formation.

## Results and discussion

**Fig. 1:**
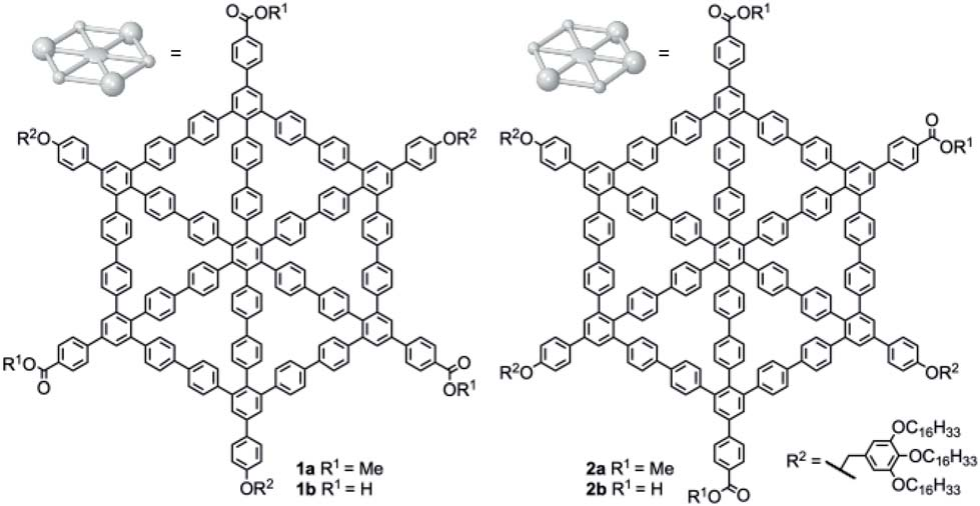
Molecular spoked wheels (MSWs) **1a/b** and **2a/b**.

Here, we report on the synthesis of *D*_3h_- and *C*_s_-symmetric MSWs **1a/b** and **2a/b** (Fig. 1) and the supramolecular nanopatterns formed by them. The compounds were prepared by cobalt-catalyzed trimerization of acetylenes with one trialkoxybenzyloxy group and one methyl carboxylate, leading to a mixture of the corresponding 1,3,5- and 1,2,4-substituted arylene hubs (Scheme 1). These wheel precursors were separated by column chromatography on silica gel, assuming that the nonzero net dipole moment of the 1,2,4-isomer leads to a lower retardation factor (*R*_f_) as compared to the 1,3,5-isomer, in which the different polarities of the ester groups are cancelled out. Final ring closure of both species was performed under Yamamoto coupling conditions, and the products were obtained as *D*_3h_-symmetric (or, 1,3,5-substituted) and *C*_s_-symmetric (or, 1,2,4-substituted) methyl esters **1a** and **2a**, from which the carboxylic acids **1b** and **2b** were obtained.

**Fig. 2:**
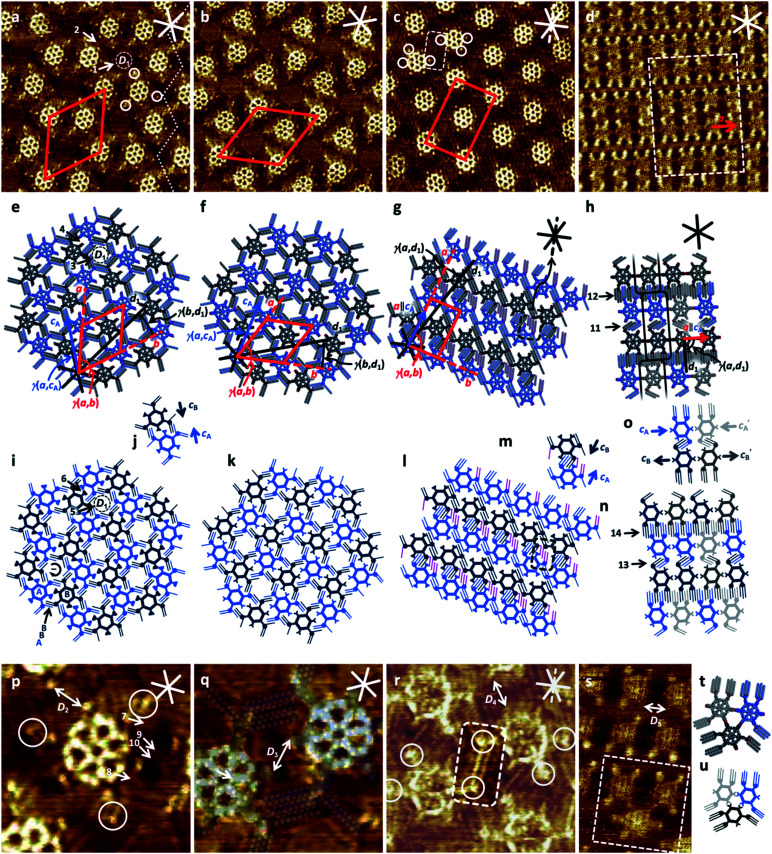
STM images and (supra-) molecular and schematic models of self-assembled monolayers of **1a** and **1b**, and of **2a** and **2b** at the solution/HOPG interface using OA as a solvent. Samples of **1a**, **1b**, and **2a** (a–c and p–r) were thermally annealed for 20 s at 100 °C; samples of **2b** (d and s) were thermally annealed for 20 s at 110 °C prior to imaging. (a), (e), (i), (j), (p) **1a** (a): 25.5 × 25.5 nm^2^, p: 7.3 × 7.3 nm^2^, a, p: *V*_S_ = −0.6 V, *I*_t_ = 10 pA, *c* = 1 × 10^−6^ M; unit cell: *a* = *b* = (8.2 ± 0.2) nm, *γ*(*a*,*b*) = (60 ± 2)°, additional packing/pore parameters: *γ*(*a*,*c*_A_) = (16 ± 2)°, *γ*(*b*,*d*_1_) = (18 ± 2)°, pore diameter: *D*_1_ = 2.8 nm, C–C distance of carboxylate groups: *D*_2_ = 1.5 nm, arrows 1, 3, and 5: hexagonal nanopore; arrows 2, 4, and 6: smaller nanopore with four intercalated OA dimers (arrows 7 to 10); (b), (f), (k), (q) **1b** (b): 25.5 × 25.5 nm^2^, (q): 7.3 × 7.3 nm^2^, (b and q): *V*_S_ = −1.6 V, *I*_t_ = 30 pA, *c* = 1 × 10^−6^ M; unit cell: *a* = *b* = (8.2 ± 0.2) nm, *γ*(*a*,*b*) = (60 ± 2)°, additional packing parameters: *γ*(*a*,*c*_A_) = (16 ± 3)°, *γ*(*b*,*d*_1_) = (18 ± 3)°, *D*_3_ = 1.5 nm; (c), (g), (l), (m), (r) **2a** (c): 25.5 × 25.5 nm^2^, *V*_S_ = −1.1 V, *I*_t_ = 11 pA, *c* = 1 × 10^−6^ M; (r): 10.5 × 10.5 nm^2^, *V*_S_ = −1.6 V, *I*_t_ = 20 pA, *c* = 10^−6^ M; unit cell: *a* = (9.3 ± 0.2) nm, *b* = (5.7 ± 0.2) nm, *γ*(*a*,*b*) = (88 ± 2)°, additional packing parameters: *a*‖*c*_A_, *γ*(*a*,*d*_1_) = (12 ± 2)°, C–C distance of carboxylate groups: *D*_4_ = 2.3 nm; (d), (h), (n), (o), (s)–(u) **2b** (d): 37.2 × 37.2 nm^2^, *V*_S_ = −0.9 V, *I*_t_ = 22 pA, *c* = 1 × 10^−6^ M; s: 10.0 × 16.9 nm^2^, *V*_S_ = −0.8 V, *I*_t_ = 15 pA, *c* = 1 × 10^−6^ M; unit vector (neglecting different backbone orientations): *a* = (4.4 ± 0.2) nm, additional packing parameters: *a*‖*c*_A_, *γ*(*a*,*d*_1_) = (90 ± 2)°, C–C distance of carboxylate groups: *D*_5_ = 2.4 nm. Side chains that are not adsorbed along the HOPG substrate but point towards the solution phase are not shown in the models. The red and solid white (black) lines indicate the unit cells (*a*, *b*) and HOPG main-axis directions (*d*_1_, *d*_2_, *d*_3_), respectively, the dashed black (white) lines in (c), (g), (r) indicate the HOPG armchair direction (*d*_4_) along which the four hexadecyloxy side chains per two molecules of **2a** are oriented. Bright and dark blue and grey colors of the models indicate molecules having different backbone orientations (*c*_A_, *c*^′^_A_, *c*_B_, *c*^′^_B_).

All compounds, as well as the aggregation of **1a** in dichloromethane, were fully characterized in solution by NMR and PL lifetime measurements (for details see ESI‡).^19^ Since the wheel precursors as well as the wheels **1a** and **1b** show identical NMR spectra, the assignment of the respective isomers is thus far based only on their different polarities (*R*_f_ values). However, a final proof of the structure of the wheels **1a/b** and **2a/b** is obtained by scanning tunneling microscopy (STM, Fig. 2). All four compounds form self-assembled monolayers (SAMs) at the solid/liquid interface of highly oriented pyrolytic graphite (HOPG) and a dilute (1–3 × 10^−6^ M) solution using octanoic acid (OA) as a solvent. The choice of solvent was optimized in thorough previous work on different MSWs.^20^ Initial studies on **1a** in OA (see ESI‡) showed that thermal treatment of the adlayers at sufficiently high temperatures (of 100–110 °C) is required for the formation of 2D nanopatterns visible by STM at ambient temperature. This requirement is not uncommon for such large molecules and is related to the high compound affinity to the substrate and the thermal activation barrier of 2D diffusion,^10*e*^ as well as kinetic trapping of molecules in amorphous film regions, where the molecules retain some residual mobility and therefore reduced visibility in the STM. The monolayers were investigated with the STM tip immersed into the solution phase (for details see ESI‡). Bright and dark image regions indicate low and high tunneling resistivities, respectively. For all MSWs dark regions in the central hub and at the corners are observed, probably due to a sterically driven electronic decoupling of these molecule parts from the substrate.^21^

**1a** forms domains (of about 100 × 100 nm^2^ size, see ESI‡) with a chiral 2D crystalline honeycomb packing (Fig. 2a, left part) that is a union of two triangular sublattices (*cf.* bright and dark blue colored molecules, Fig. 2e and i). The structure is triporous, *i.e.* a nanopattern containing pores of three different shapes or sizes, while it is biporous with respect to the intermolecular pores, and each molecule inherits another six smaller intramolecular triangular pores. The domains are separated by uncovered regions (see ESI‡) or nonporous domain boundaries (Fig. 2a, right part).^22^ A unit cell with parameters *a* = *b* = (8.2 ± 0.2) nm and *γ*(*a*,*b*) = (60 ± 2)° containing two molecules with identical conformations but different (relative) orientations *c*_A_ and *c*_B_ with *γ*(*c*_A_,*c*_B_) = 180° (or 60°/120°, Fig. 2j) is indexed to the 2D lattice. The phenylene units carrying the three hexadecyloxy side chains appear as bright dots (*cf.* white circles in Fig. 2a and p), and prove the overall threefold symmetry of the backbone. Its side chains are oriented along two different HOPG zig–zag directions (with an angle of 120° relative to each other). Hexagonal nanopores (arrows 1, 3, and 5 in Fig. 2a, e, and i) of diameter *D*_1_ = 2.8 nm are surrounded by six intermolecular triples of hexadecyloxy side chains of ABB ordering (*cf.*Fig. 2i) with the innermost chains aligned in clockwise (−) (Fig. 2a, e, i and p) or counterclockwise (+) orientation (see ESI‡).^23^ While some contrast variation is observed in these pores (Fig. 2a), we cannot assign a specific arrangement of intercalated OA molecules. We relate this to rapid dynamics on the timescale of STM and/or a mismatch of the solvent cluster size relative to the dimensions of the nanopore (*cf.* ESI‡). Smaller intermolecular nanopores (arrows 2, 4, and 6) between pairs of molecules are surrounded by two side-chain triples of ABB ordering with the innermost chains aligned in either clockwise (−) or counterclockwise (+) orientation, and two MSW backbone segments, containing the methyl carboxylate units in a distance of *D*_2_ = 1.5 nm (Fig. 2p).^24^ In these pores, four intercalated hydrogen-bonded OA dimers are clearly resolved by STM (arrows 7 to 10 in Fig. 2p, S4c and d in the ESI‡). **1b** (Fig. 2b) forms a packing identical to **1a** despite the presence of three carboxylic acid functions per molecule. As observed for the esters, the carboxylic acids point towards the smaller nanopore and point past each other. They do not form intermolecular hydrogen-bonded (homo)dimers, in contrast to trimesic acid^11*a*,*b*^ and its larger homologues.^11*c*,*d*^ The functional group distances (*D*_3_ = 1.5 nm, Fig. 2q) are unaltered compared to **1a**. This similarity demonstrates that the alkyl interdigitation pattern and the interaction of the 1,3,5-isomer with the HOPG substrate is so stable that it overcomes the driving force of carboxylic acids to form hydrogen-bonded dimers. However, OA as a non-inert solvent can coordinate to the carboxylic acid functions and thus prevent dimerization.^25^ The absence of any OA dimers in the packing of **1b** (which is in contrast to **1a**, *cf.* arrows 7 to 10 in Fig. 2p and ESI‡) provides further evidence for an altered solvent intercalation scenario with less dense packing and therefore dynamics faster than the timescale of STM (*cf.* Fig. S7 in the ESI‡). Moreover, in contrast to the acceptably resolved STM images of 2D crystals of **1a** after adsorption from a solution of **1a** in 1-phenyloctane (PHO; *cf.* Fig. S6 in the ESI‡), and the well resolved 2D crystalline packings of **1b** using OA (Fig. 2b, q, and S9 in the ESI‡), we observed a highly disordered monolayer for **1b** using PHO (*cf.* Fig. S10 in the ESI‡). More precisely, **1b** showed some tendency to form a honeycomb-like assembly at least in some surface regions, however, with irregular distances and alignments. In addition, some contrast variation, probably due to solvent intercalation, is found in the large hexagonal nanopores of **1b** using OA as solvent, as it was already discussed for **1a**.

**2a** forms an oblique packing (Fig. 2c, domain sizes of about 40 × 40 nm^2^, see ESI‡) to which a unit cell of *a* = (9.3 ± 0.2) nm, *b* = (5.7 ± 0.2) nm, *γ*(*a*,*b*) = (88 ± 2)° containing two molecules (with backbones rotated by 180°) is indexed. Of each pair of molecules, 14 of the 18 hexadecyloxy side chains (shown in bright and dark blue color in Fig. 2g, l, and m) are oriented along the zig–zag directions of the HOPG substrate, and therefore define the overall packing. As a result of steric constraint, the remaining four side chains of each pair of **2a** (shown in purple color in Fig. 2g, l, and m and marked by dashed boxes in Fig. 2c, g, l, and r) cannot adsorb along one of the HOPG zig–zag directions. We interpret the medium bright (Fig. 2c and ESI‡) to bright (Fig. 2r) lines as hexadecyloxy side chains that are aligned along one of the three HOPG armchair directions, indicated as dashed lines in Fig. 2c, g, and r. Being energetically less favorable,^13^ domains of (cyclo-) alkanes oriented along the armchair direction in a constrained environment have been observed previously,^14^ although this arrangement is rather rare. In addition, in the STM images medium-bright features similar in shape and length to those of the alkoxy side chains are clearly visible, which cannot be attributed to MSW entities. As in the case of earlier results, OA (*i.e.* solvent) molecules form dimers on the surface, which fill the otherwise uncovered space on the surface, align in parallel to the (MSW) alkoxy chains, and therefore stabilize the packing. As such, we suspect that the alkoxy chains aligned along the less favorable armchair direction are stabilized by two intercalating solvent dimers, which are clearly visible in the STM images (see ESI‡).

**Fig. 3:**
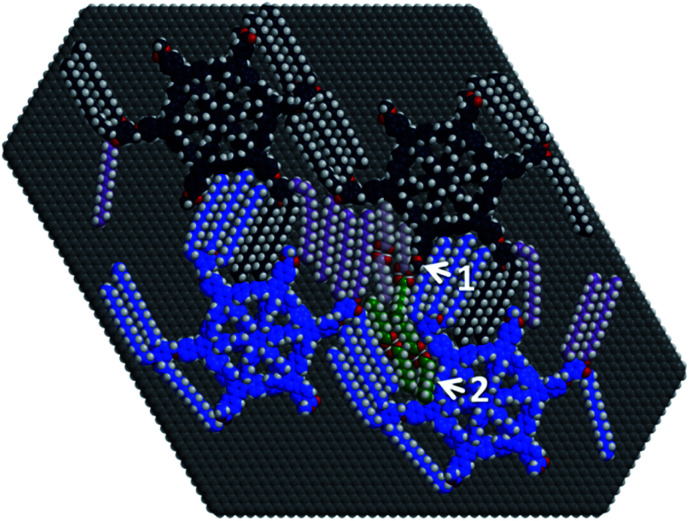
Structure fragment (of 3652 atoms) of four MSWs **2a** (bright/dark blue and purple) with four OA dimers (pale purple and green), optimized at the GFN-FF level of theory on a hydrogen terminated graphene monolayer (C_5644_H_190_). Arrow 1 depicts a methoxybenzoate group, tilted due to steric strain. Arrow 2 indicates an OA molecule pushed out of the plane due to steric interaction.

To scrutinize this assumption, for this highly specific question we performed geometry optimizations of four MSWs **2a** with four intercalated OA dimers (shown in pale purple and green) on a graphene cutout (C_5644_H_190_) containing the relevant monolayer regions (Fig. 3, with 3652 atoms). Our calculations were conducted with a new general force field (called GFN-FF). In brief, this method outperforms other FFs in terms of generality and accuracy, and may reach even more elaborate quantum-mechanical methods in terms of performance. The method therefore appears suitable to verify the robustness of the rather unexpected structural model found in Fig. 2, helping us to clearly distinguish the assignment of STM image contrasts to adsorbate film structures from possible misleading image artefacts. The calculations require only starting coordinates and elemental composition as input, basically treating any chemical structure consisting of elements up to radon automatically, and allows access to systems composed of roughly 9500 atoms arranged in, *e.g.*, highly unsaturated components (such as arylenes and graphite or graphene), which would be prohibitively expensive using even fast semi-empirical quantum mechanical tight-binding calculations (*e.g.* GFN2-xTB) due to the number of atoms involved. Additional details of the calculations are found in the ESI‡ and ref. 26 and 27. First, the results (Fig. 3) agree perfectly with the models (*cf.*Fig. 2g, l, and m) fitted to the data obtained in STM imaging (Fig. 2c and r).^28^ More precisely, after geometry optimization of the aforementioned starting geometries by GFN-FF, the alkoxy side chains in Fig. 3 (bright and dark blue, purple) and the OA dimers (pale purple, green) retain their general orientations, even if they are orientated along the unfavorable HOPG armchair directions.^13*a*,14^ Moreover, hydrogen bond dimerization of the OA carboxylic acid groups is also retained during optimization. Both these results resemble the high degree of descriptiveness of non-covalent interactions by the GFN-FF method. A more detailed discussion of this issue is given in the ESI.‡

As for **1a**, the ester groups in **2a** do not interact, and are *D*_4_ = 2.3 nm apart (Fig. 2r). However, while both ester **1a** and acid **1b** MSWs form the same pattern on HOPG, this does not hold for the ester **2a** and acid **2b** MSWs. The 1,2,4-substitution pattern in **2b** allows a packing of the molecules such that the carboxylic acids drive the formation of 1D supramolecular chains *via* hydrogen bonds of the acids in the 1- and 4-positions – despite using OA as a solvent. To this 1D packing, a unit vector of *a* = (4.4 ± 0.2) nm is indexed, however, neglecting different backbone orientations. According to this packing distance along *a*, we assume that only 10 of the 12 side chains along arrow 12 (Fig. 2h) or arrow 14 (Fig. 2n) can adsorb along the HOPG main axis directions (assuming the parallel conformer),^13*a*^ while the residual chains point towards the solution phase. In terms of their packing distances, the alkoxy chains have to adapt to the distances given by backbone sizes and cyclic carboxylic acid dimer packing distances. In addition, only 2 of 3 chains per molecule can adopt the conformation marked by arrow 11 (Fig. 2h) or arrow 13 (Fig. 2n), while the third chain may share the remaining space with intercalated solvent molecules.^29^ Regarding the 1D packing, **2b** can be viewed as a larger homologue of terephthalic acid.^11*b*^ The carboxylic acids in the 2-position in turn do not interact with other carboxylic acids and are spaced apart *D*_5_ = 2.4 nm in the nearest possible orientations. The two possible orientations of the molecules (*c*_A_ and *c*^′^_A_ in Fig. 2) within a chain occur randomly, introducing some disorder. The reduced STM image resolution of **1b** (compared to the images of **1a**, **1b**, and **2a**) is attributed to the reduced order owing to the aforementioned factors. However, each molecule determines the orientation of the other molecule (*c*_B_ or *c*^′^_B_, respectively) by side-chain interactions (arrows 11 and 13), whereas the orientation of the molecules in the subsequent row is not influenced (arrows 12 and 14). Occasionally, hydrogen-bonded trimers are observed (Fig. 2s–u), which result from the interaction of carboxylic acids in the 1- and 2-positions, which is sterically unfavorable for phthalic acid.^11*b*^ In addition, trapezoid superstructures containing 11 and 12, as well as both enantiomers of a rhomboid superstructure containing 10 molecules were observed under alike imaging conditions (see Fig. S17 in ESI‡). These moieties include carboxylic acid dimers at the 1-/4-positions as well as at the 1-/2-positions. The superstructures formed are interpreted as a consequence of two energetically similar packaging motives as a result of the increased size as compared to benzene dicarboxylic acids.^11*b*^

## Conclusion

In summary, we described low-symmetry MSWs with ester groups in the 1,3,5- or 1,2,4-positions, respectively, which upon hydrolysis give the corresponding free acids that can be viewed as giant alkoxy-substituted homologues of trimesic and trimellitic acids. Besides spectroscopic, spectrometric, and chromatographic methods, STM is an integral part of the structure assignment. All four MSWs (**1a/b** and **2a/b**) form self-assembled monolayers on HOPG, and were studied by STM with submolecular resolution. These investigations show that the *D*_3h_-symmetric 1,3,5-isomer forms the same packing regardless of the exact carboxylic group substitution (*i.e.* ester or acid), implying that the 2D crystal structures are dominated by the hexadecyloxy side chains. Contrary, the packing of the *C*_s_-symmetric 1,2,4-isomer is strongly influenced by the absence or presence of substituents prone to forming hydrogen-bonded dimers. Alkoxy side chains play only a subordinate role so that in **2b** the superstructure formation is driven by dimerization of the carboxylic acids. The monolayer of **2a** acts as a template for the armchair-oriented adsorption of alkyl chains, including OA solvent molecules. A complex computational analysis by means of GFN-FF could confirm this rather unusual STM observation and demonstrates the potential of the method to go far beyond the conventional level of STM image description using space-filling models.

## Author contributions

S. H. elaborated the research concept and acquired funding. C. S. and T. L. S. synthesized, isolated and characterized the compounds and studied aggregation of the MSWs. T. J. K. and J. B. performed STM investigations and modelling of MSW nanopatterns and together with S.-S. J. visualized and curated the data. M. B., J. K., and S. G. performed theoretical investigations incl. design of computer programs, code implementation and/or testing as well as data visualization and analysis. T. E. performed optical spectroscopic investigations. J. M. L., S. G., S. H., and S.-S. J. administrated the project incl. supervision and validation of the results. The original draft was written through contributions of all authors. T. J. K., J. B., S. H., and S.-S. J. revised and edited the final manuscript.

## Conflicts of interest

There are no conflicts to declare.

## Supplementary Material

SC-012-D1SC01381E-s001
