# Supplementary material for: Nanopatterns of molecular spoked wheels as giant homologues of benzene tricarboxylic acids
Source: Chem Sci. 2021 Jun 9;12(27):9352–8. doi: 10.1039/d1sc01381e (PMC8278972; doi:10.1039/d1sc01381e)
Supplement: SC-012-D1SC01381E-s001 [file SC-012-D1SC01381E-s001.pdf]

Electronic Supplementary Material (ESI) for Chemical Science.
This journal is © The Royal Society of Chemistry 2021

Supporting Information

Nanopatterns of Molecular Spoked Wheels as Giant Homologues of Benzene Tricarboxylic Acids

Tristan J. Keller,[a,‡] Christopher Sterzenbach,[a,‡] Joshua Bahr,[a] Taria Schneiders,[a] Markus Bursch,[b] Julia Kohn,[b] Theresa Eder,[c] J. M. Lupton,*[,c] Stefan Grimme*[,b], Sigurd Höger*[,a], and Stefan-S. Jester*[,a]

a) Kekulé-Institut für Organische Chemie und Biochemie,

Rheinische Friedrich-Wilhelms-Universität Bonn,

Gerhard-Domagk-Straße 1, 53121 Bonn, Germany

b) Mulliken Center for Theoretical Chemistry,

Rheinische Friedrich-Wilhelms-Universität Bonn,

Beringstraße 4, 53115 Bonn, Germany

c) Institut für experimentelle und angewandte Physik,

Universität Regensburg,

Universitätsstraße 31, 93053 Regensburg, Germany

Corresponding authors. E-mail: stefan.jester@uni-bonn.de; hoeger@uni-bonn.de; grimme@thch.uni-bonn.de; john.lupton@physik.uni-regensburg.de

Contents

**1**	**General information**	**S3**
1.1	Materials and equipment	S3
1.2	GPC experiment	S3
1.3	STM experiment	S3
**2**	**Additional STM images**	**S5**
2.1	Additional STM images of **1a**	S5
2.1.1	Monolayers of **1a** using OA as a solvent	S5
2.1.2	Monolayers of **1a** using PHO as a solvent	S11
2.2	Additional STM images of **1b**	S12
2.2.1	Monolayers of **1b** using OA as a solvent	S12
2.2.2	Monolayers of **1b** using PHO as a solvent	S15
2.3	Additional STM images of **2a** using OA as a solvent	S16
2.4	Additional STM images of **2b** using OA as a solvent	S21
**3**	**Theoretical investigations of a cutout of the nanopattern of 2a**	**S27**
3.1	General aspects	S27
3.2	Investigation of **2a** including solvent molecules on graphene	S27
3.3	Theoretical experiments on *n*-alkane model systems to investigate energetic effects	S28
3.3.1	General aspects	S28
3.3.2	Rotational barrier of *n*-hexane on graphene	S29
3.3.3	Chain length and number-of-chain dependence of rotational barriers	S30
3.3.4	Scanning the graphene grid to evaluate translational barriers	S31
3.4	Computed STM images of MSWs	S36
**4**	**Synthesis and characterization**	S39
4.1	Depiction of the synthesis	S39
4.2	Synthesis of the compounds	S41
4.3	Melting	S56
**5**	**Aggregation of 1a**	S57
**6**	**Photophysical properties**	**S59**
6.1	UV/vis absorption spectra	S59
6.2	Photoluminescence lifetime decay of **1a** and *p*-quaterphenyl	S60
6.3	Streak camera images of **1a** and *p*-quaterphenyl	S61
**7**	**References**	**S62**

1 General information

1.1 Materials and equipment

Reagents were purchased at reagent grade from commercial sources and used without further purification. All air-sensitive reactions were carried out using standard Schlenk techniques under argon. Reaction solvents (THF, piperidine, dichloromethane, pyridine, triethylamine, toluene) were dried, distilled, and stored under argon according to standard methods, workup solvents were either used in "p.a." quality or purified by distillation (dichloromethane, cyclohexane). Prior to characterization and further processing, all solids and oils were dried at r.t. under vacuum. ^{1}H and ^{13}C NMR spectra were recorded on a Bruker Bruker Avance I 300 MHz, Bruker Avance I 400 MHz, Bruker Avance III HD 500 MHz Prodigy and Bruker Avance III HD 700 MHz Cryo (300.1, 400.1, 500.1 and 700.1 MHz for ^{1}H and 75.5, 100.6, 125.8 and 176.0 MHz for ^{13}C). Chemical shifts are given in parts per million (ppm) referenced to residual ^{1}H or ^{13}C signals in deuterated solvents. All NMR spectra were recorded at r.t. unless otherwise described. Mass spectra were measured on a Finnigan ThermoQuest MAT 95 XL (EI-MS), a Sektorfeldgerät MAT 90 (EI-MS), a Bruker Daltonics micrOTOF-Q (ESI-MS, APCI), a Thermo Fisher Scientific Orbitrap XL mass spectrometer (ESI-MS), a Bruker Daltronics autoflex TOF/TOF (MALDI-MS; matrix material: DCTB, no salts added) and an ultrafleXtreme TOF/TOF of the Bruker Daltonik company (MALDI-MS; matrix material: DCTB, no salts added). *m/z* peaks smaller than 10 % (compared to the basis peak) are not reported. Thin layer chromatography was conducted on silica gel coated aluminium plates (Macherey-Nagel, Alugram SIL G/UV254, 0.25 mm coating with fluorescence indicator). Silica gel Kieselgel 60 (Merck, 0.040-0.063 mm) was used as the stationary phase for column chromatography. UV/vis absorption were recorded on a Perkin Elmer Lambda 18 and fluorescence emission spectra on a LS-50B Perkin Elmer spectrophotometer using 10 mm quartz cuvettes. Microwave assisted reactions were performed in a CEM Discover Labmate instrument (maximal power: 300 W). Melting points were measured using an optical microscope equipped with a heating table (Leica DMLB, Leica LMW, Testo 965).

1.2 GPC experiment

Gel permeation chromatography (GPC) was performed in THF (HPLC grade, stabilized with 2.5 ppm BHT) at r.t. GPC analyses were run on an Agilent Technologies system at a flow rate of 1 mL/min using an IsoPump G1310 A, a diode array UV detector (G1315B) and PSS columns (Polymer Standards Service, Mainz, Germany; 10^{2}, 10^{3}, 10^{5} and 10^{6} Å, 5 µ, 8 × 300 mm). All molecular weights were determined versus PS calibration (PS standards from PSS, Mainz, Germany).

For the preparative separation, a Shimadzu Recycling GPC system, equipped with a LC-20 AD pump, a SPD-20 A UV detector and a set of three preparative columns from PSS (either SDV 10^{3} Å, 5 µ, 20 × 300 mm or SDV preparative linear S, 5 µ, 20 × 300 mm) with precolumn (SDV, 5 µ, 20 × 50 mm) was employed. The system operated at a flow rate of 5 mL/min, THF, 35 °C.

1.3 STM experiment

Scanning tunneling microscopy (STM) was performed under ambient conditions (r.t.) at the solution/solid interface, using 1,2,4-trichlorobenzene (TCB), 1-phenyloctane (PHO) or 1-octanoic acid (OA) as solvents and highly oriented pyrolytic graphite (HOPG) as substrate. In a typical experiment, 0.2 µL of a 1×10^{-6} to 3×10^{-6} M solution of the compound of interest was dropped onto a freshly cleaved HOPG substrate at elevated temperature (80 °C to 110 °C), kept at this temperature for 10 s to 20 s, and allowed to cool to r.t. before the STM measurements were performed with the tip immersed into the solution. Bias voltages between –1.9 V and –0.6 V and tunneling current set points in the range of 9 pA to 39 pA were applied to image the supramolecular

adlayers shown here. The experimental setup consists of an Agilent 5500 scanning probe microscope that is placed on a Halcyonics actively isolated microscopy workstation. It is acoustically shielded with a home-built box. Scissors cut Pt/Ir (80/20) tips were used and further modified after approach by applying short voltage pulses until the desired resolution was achieved. HOPG was obtained from TipsNano (*via* Anfatec) in ZYB-SS quality. All STM images (unless otherwise noted) were calibrated by subsequent immediate acquisition of an additional image at reduced bias voltage, therefore the atomic lattice of the HOPG surface is observed which is used as a calibration grid. Data processing, also for image calibration, was performed using the SPIP 5 (Image Metrology) software package. (Supra-) molecular modelling was performed using Wavefunction Spartan ’10, ’16, and ‘18.

2 Additional scanning tunneling microscopy images

2.1 Additional STM images of **1a**

*2.1.1 Monolayers of **1a** using OA as a solvent*

Self-assembled monolayers (SAMs) of **1a** at the solid/liquid interface of highly oriented pyrolytic graphite (HOPG) and a solution using octanoic acid (OA) as solvent are investigated by scanning tunneling microscopy (STM; Figure S1).

In a first experiment, a droplet of **1a** in OA ($c = 1 \times 10^{-5}$ M) is applied to a piece of HOPG at 80 °C, and the sample is kept at this temperature for 20 s before it is allowed to cool to r.t. STM shows a rather small amorphous region of **1a** (arrow 1, Figure S1a) that is located close to a step edge (arrow 2).

When another sample is prepared by using a $c = 1 \times 10^{-6}$ M solution of **1a** in OA, but the sample is (again for 20 s) thermally annealed at an increased temperature of 100 °C, 2D crystalline domains of about 100^2 nm^2 lateral size are observed (Figure S1b). This shows that an elevated temperature is required for the rearrangement of **1a** into 2D crystalline nanopatterns at the solid/liquid interface on HOPG. The dotted line (arrow 3, Figure S1b) indicates an island rim.

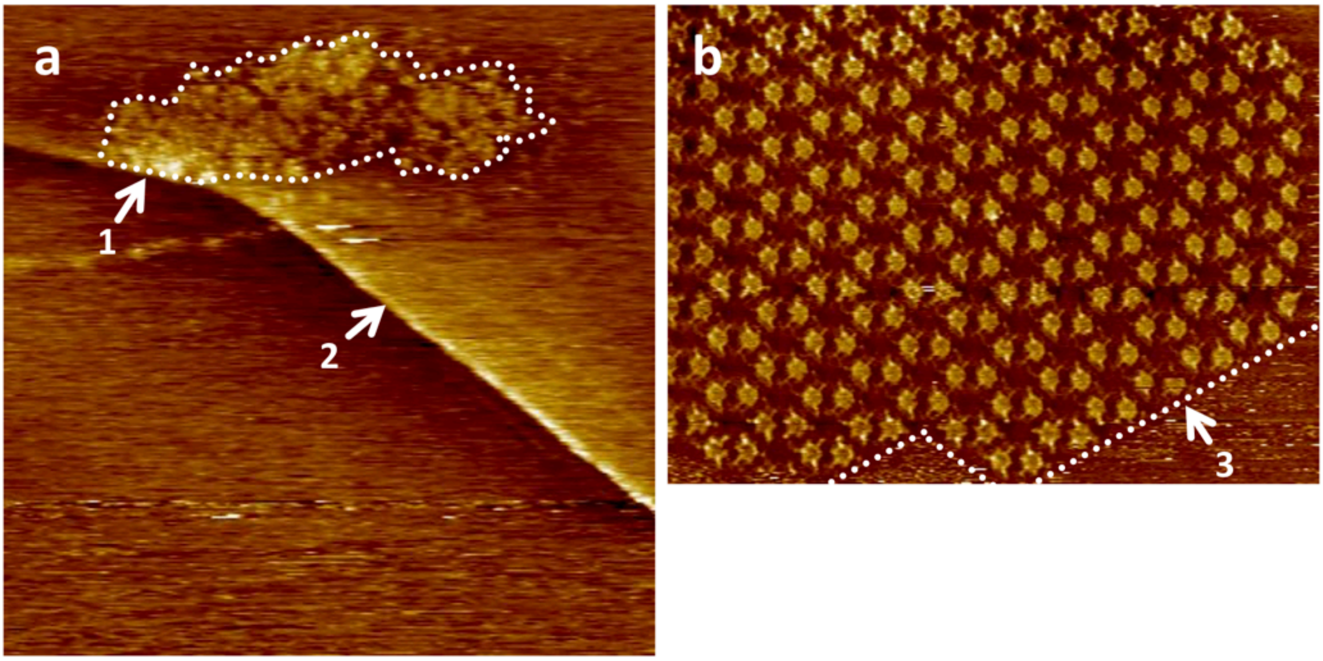

Figure S1. STM images of **1a** at the solid/liquid interface of HOPG and a solution of **1a** in OA. (a) Overview STM image (163×163 nm^2 (internal scanner calibration), $c = 1 \times 10^{-5}$ M, sample thermally annealed for 20 s at 80 °C, $V_S = -1.9$ V, I_t = 19 pA); (b) overview STM image (97 × 118 nm^2 (internal scanner calibration), $c = 10^{-6}$ M, thermally annealed for 20 s at 100 °C, $V_S = -1.6$ V, I_t = 9 pA).

A high-resolution STM image of a self-assembled monolayer of **1a** ($c = 1 \times 10^{-6}$ M, after thermal annealing for 20 s at 100 °C) is shown in Figure S2. The white dotted zig-zagged lines subdivide the image into regions A, B, and C. Regions A and C are covered by the biporous chiral nanopattern of **1a** (that forms large domains, as previously described, cf. Figure S1b). To this nanopattern, a unit cell of $a = b = (8.2 \pm 0.2$ nm), $\gamma(a,b) = (60 \pm 2)°$ is indexed (marked in red color in Figure S2). Two different types of intermolecular nanopores occur in this packing – a hexagonal nanopore surrounded by alkoxy chains (marked by white dashed hexagon 1) and a smaller nanopore confined by alkoxy chains and MSW backbones (marked by white dashed box 2). The methyl ester

units point into this (smaller) nanopore, and appear as dark shadows next to bright dots (attributed to the phenylene units, cf. arrows 3 and 4). Moreover, in region B of Figure S2, two lines of densely packed molecules are observed (*i.e.* the molecules along the dotted white lines). However, this packing is only observed as a line defect, and a 2D periodic non-porous/denser nanopattern similar to the packing of these two lines of **1a** was not observed.

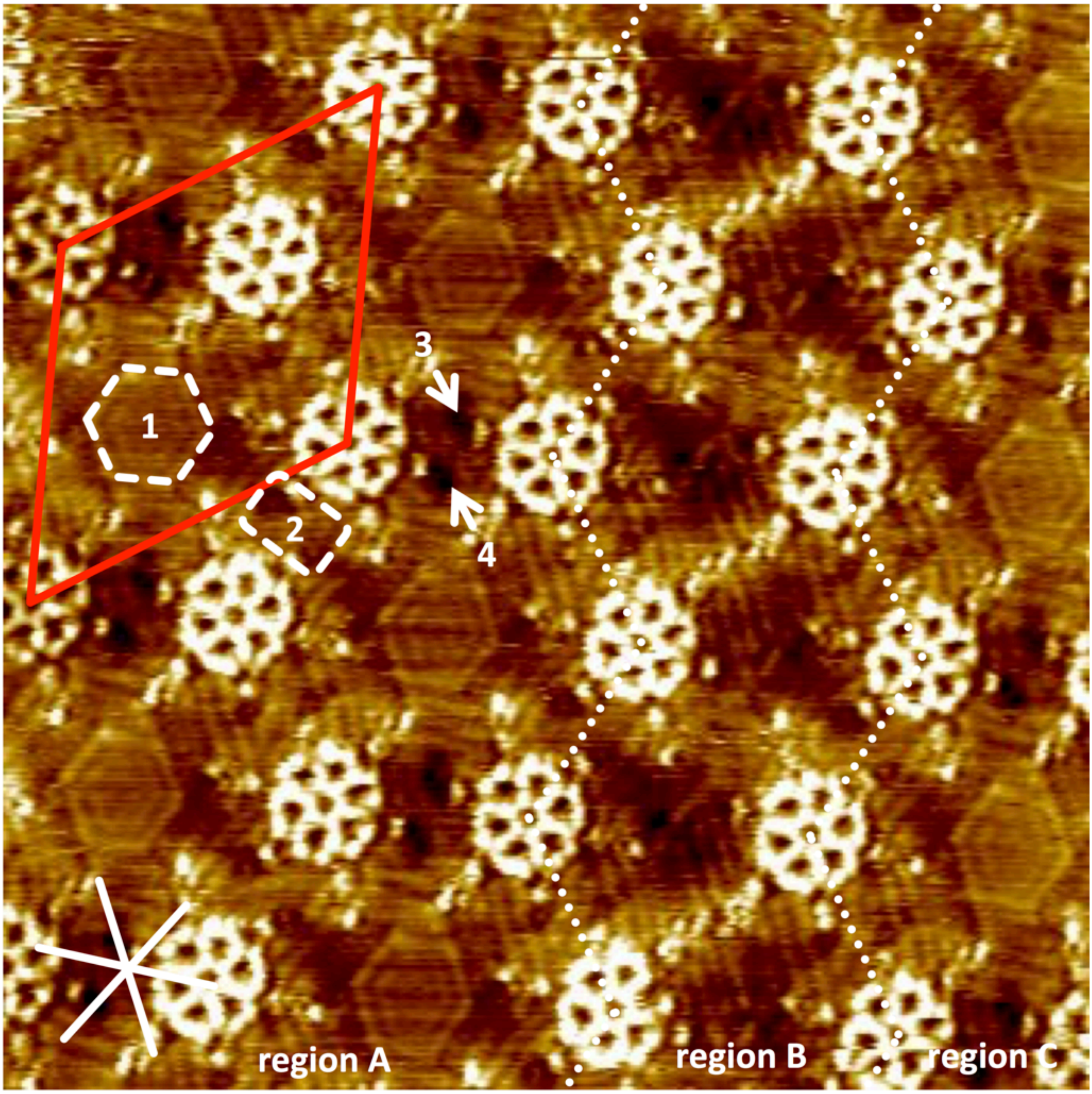

Figure S2. High-resolution STM image of **1a** at the solid/liquid interface of HOPG and **1a** in OA (25.2 × 25.2 nm^2, c = 1 × 10^{-6} M, thermally annealed for 20 s at 100 °C, V_S = –0.6 V, I_t = 10 pA). Red and white lines indicate the unit cell and the HOPG main axis directions, respectively. White dotted lines mark two lines of densely packed **1a**, that form a domain boundary.

Figure S3a shows a supramolecular model of the porous chiral nanopattern of **1a** (cf. Figure S1b and regions A and C in Figure S2), Figures S3b and c show corresponding schematic models, and all of them are enlarged versions of Figures 2e, i, and j (Main Text). The hexagonal intermolecular nanopore is highlighted by area 1 (dashed black lines), whereas the smaller intermolecular nanopore (dashed black lines) is indicated by arrow 2. Two different backbone orientations c_A and c_B are observed and highlighted by light and dark blue colors.

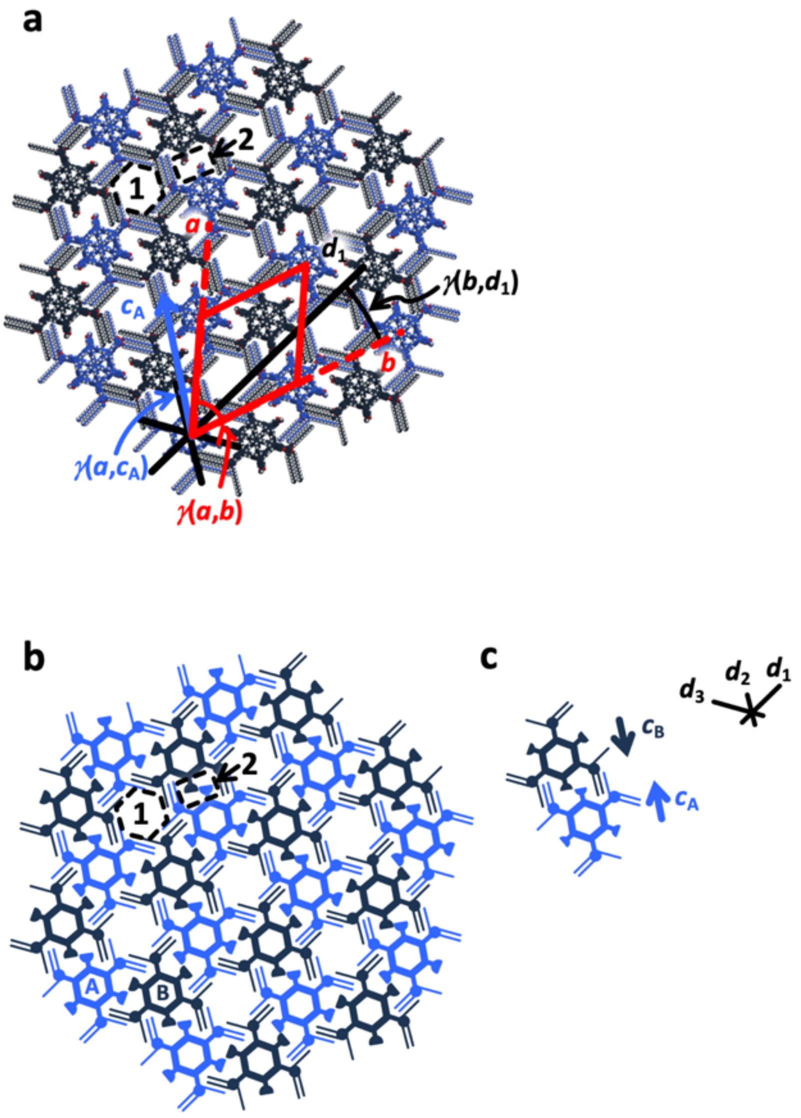

Figure S3. (a) Supramolecular and (b)-(c) schematic models of the nanopatterns of **1a**. The red lines indicate the indexed unit cell $a = b = (8.2 \pm 0.2\ \text{nm})$, $\gamma(a,b) = (60 \pm 2)°$. The light and dark blue lines indicate the backbone orientations c_A and c_B which are oriented in antiparallel direction, with an angle of $\gamma(a,c_A) = (16 \pm 2)°$. The black lines indicate the HOPG main axis directions d_1, d_2, and d_3 (along which all hexadecyloxy side chains are oriented). The indexed unit cell is oriented with $\gamma(b,d_1) = (18 \pm 2)°$ relative to one of the HOPG main axis directions, d_1. 1 and 2 indicate two different types of intermolecular nanopores.

Figures S4a-c show high-resolution STM images of the same enantiomer of the porous chiral nanopattern of **1a**, as it was observed in Figure S2 and shown in the supramolecular and schematic models in Figure S3.

In particular, Figure S4a shows an STM image of one molecule of **1a** that allows to distinguish the three benzoic acid ester units (marked with dashed white circles) from the three aromatic moieties of the

tri(hexadecyloxy)benzyloxy groups (highlighted by solid white circles). These structural features confirm the threefold (D_{3h}) symmetry of the MSW backbone and allow for a precise differentiation of **1a** from its C_s symmetric isomer **2a** (cf. Figure 1 (Main Text)) which will be described in chapters 2.3 and 2.4.

The C–C distance of the carboxylate groups of two adjacent molecules of **1a** (Figure S4a) is D_2 = 1.5 nm.

Figures S4b and c show high-resolution images of type 2 nanopores (cf. Figure S2 as well as S3a and b). In addition, parts of two type 1 (hexagonal) nanopores are seen on the left and right image sides and are indicated by transparent dashed white lines. Each of these hexagonal nanopores is surrounded by six triples of hexadecyloxy side chains, two of which are marked by arrows 1 to 3 and 4 to 6 in Figure S4b, which also indicate the directions from the ether units to the terminal methyl groups. All three chains of each triple originate from four different tri(hexadecyloxy)benzyloxy groups of two adjacent molecules. The chains marked by arrows 1 and 6 are oriented in a clockwise fashion with respect to the adjacent hexagonal nanopores (cf. "–" sign and circular arrow, for definition see also Figure 2i (Main Text)). The remaining four alkoxy chains (cf. arrows 2 to 5) originate from the two opposing groups, respectively. The side chains of each tri(hexadecyloxy)benzyloxy group are oriented (with an angle of 120° relative to each other) along two different main axis directions, d_1 and d_3, of the graphite substrate (see Figure S3c).

Another more detailed insight is possible for the type 2 nanopores in Figure S4c, which is acquired with different bias voltage settings as compared to Figure S4b. As clearly seen in Figure S4c, this pore type is densely filled with eight OA (solvent) molecules. These are marked by arrows 7 to 10, and are highlighted in green and pale purple colors in the supramolecular model shown in Figure S4d. These solvent molecules form hydrogen-bonded dimers, where two of these dimers (arrows 7 and 10, green) align in parallel with the adjacent hexadecyloxy chains and are oriented along the main axis directions of graphite (white asterisk), *e.g.* direction d_2 in Figure S4c. The two other dimers (arrows 8 and 9, pale purple) densely fill the remaining space of the type 2 nanopore and are therefore not oriented along one of the three HOPG main axis directions d_1-d_3, but along direction d_4 with $\gamma(d_2,d_4)$ = (30 ± 3)° (cf. model in Figure S4d). As a result of the intercalation, the solvent molecules adsorbed in the type 2 nanopore are highly constrained, and therefore are visible by STM. In addition to the matching size of the type 2 nanopore with the OA dimers marked by 8 and 9 (Figure S4c) and pale purple color (Figure S4d), as well as the van der Waals interaction with the substrate and the pore walls, weak secondary hydrogen bond interactions of the carboxylic acid groups of the intercalated solvent molecules with the carboxylic acid methyl ester units of the MSWs seem conceivable.

Moreover, depending on the exact STM imaging conditions, certain regions of the aromatic backbone differ in contrast. Some phenylene rings appear darker or remain invisible (*e.g.* red arrows 11, 12, 14, and 15 in Figures S4c and d) as compared to the other phenylene rings (*e.g.* green arrows 13 and 16 in Figure S4c and d) in their proximity. Figures S4e and f aim at a possible explanation for this contrast phenomenon. Two different orientations of the phenylene rings of the MSW backbone are distinguishable. One (marked in red color) is oriented in parallel with respect to the surface, while the other one (marked in green color) is tilted as compared to the red phenylene ring. For the latter, the distance between the substrate and ring is shortened, and the ring protrudes higher from the surface. In the STM experiment, this may (aside from the topography change) correspond with a lower tunneling resistivity resulting in a brighter image region (marked *e.g.* by arrow 13) in the STM image. Due to the tilt of the surrounding phenylene rings, the phenylene rings marked in red (Figures S4e and f as well as red arrows 12 and 15 in Figures S4c and d respectively) is slightly lifted from the underlying substrate and therefore most probably is electronically decoupled from the conducting surface. This may (together with the topography change) correspond with a higher tunneling resistivity, resulting in a darker image region. A similar contrast phenomenon is observed for the tetraaryl-substituted phenylene rings located at the

corners of the rim segment which are also oriented parallel with respect to the surface (see arrows 11 and 14 in Figures S4c and d, respectively).

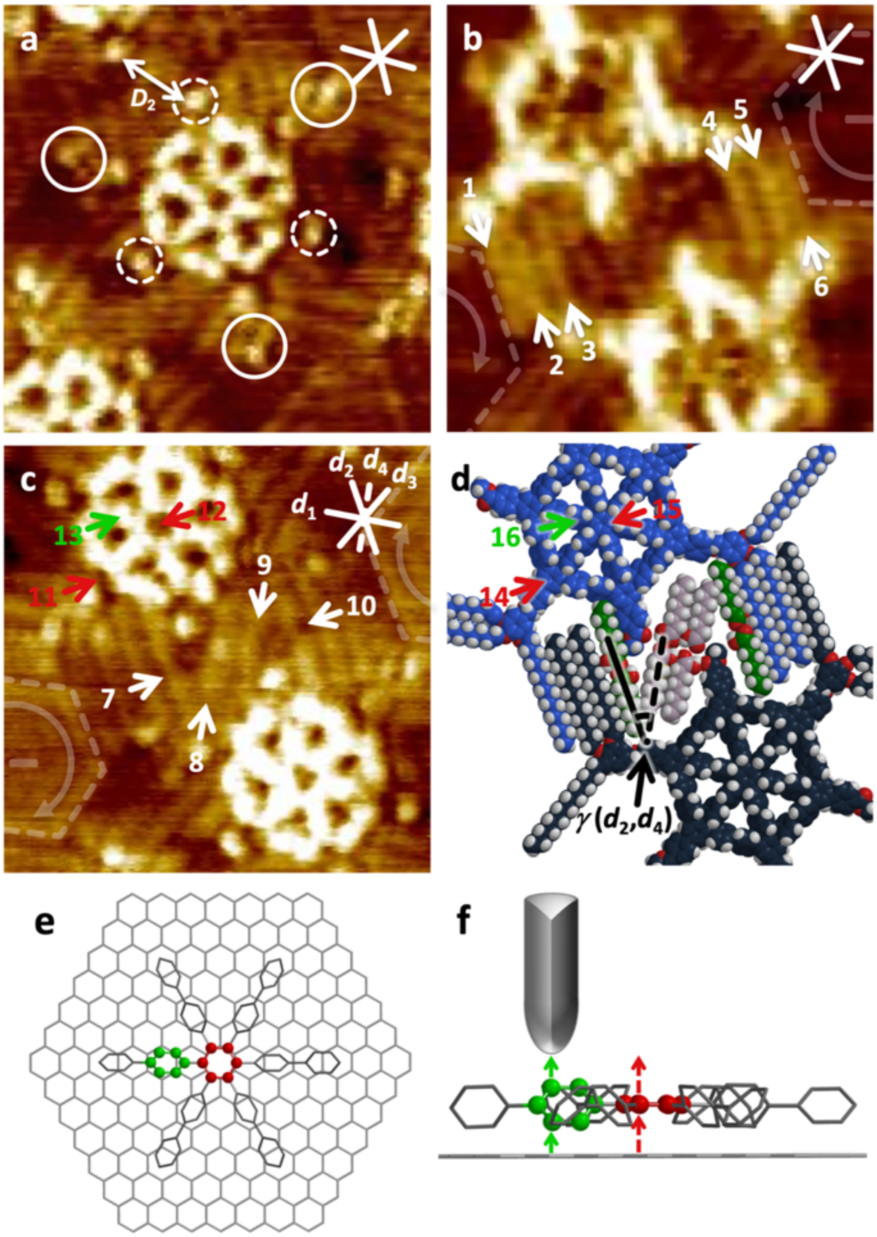

Figure S4. (a)-(c) High-resolution STM images and (d) supramolecular model of **1a** at the solid/liquid interface of HOPG and **1a** dissolved in OA (a: reprint of Figure 2p (Main Text) with additional assignments, 7.3 × 7.3 nm^2, c = 1 × 10^{-6} M, V_S = –0.6 V, I_t = 10 pA; b: 7.0 × 7.0 nm^2, c = 1 × 10^{-6} M, V_S = –1.6 V, I_t = 29 pA; c: 7.0 × 7.0 nm^2, c = 1 × 10^{-6} M, V_S = –0.7 V, I_t = 12 pA, all samples were thermally annealed for 20 s at 100 °C prior to imaging). Solid and dashed circles in (a) indicate aromatics of the tri(hexadecyloxy)benzyloxy groups and benzoic acid ester groups, respectively. Arrows 1 to 6 in (b) indicate hexadecyloxy side chains that are oriented along the HOPG main axis directions d_1 to d_3 (white solid lines). Arrows 7/10 and 8/9 in (c) indicate intercalated OA solvent molecules aligned along d_2 and d_4 (white dashed line, one of the HOPG armchair directions), $\gamma(d_2,d_4)$ = (30 ± 3)°, respectively. (e) shows the central fragment of the MSW backbone (top view) on a graphene cutout; (f) shows a similar fragment (side view) and a schematic representation of the imaging process (tip not to scale). Hydrogen atoms in (e) and (f) are omitted for the sake of visual clarity. Two different phenylene units are highlighted in green and red colors, respectively. Continuous green and dashed red arrows in (f) represent regions of low and high tunneling resistivities, that appear as bright (*e.g.* arrows 13 and 16) and dark (*e.g.* arrows 11, 12, 14, and 15) regions in the STM image in (c), respectively.

Recently, we were able to image TCB molecules in different sized supramolecular nanopores of alkoxy-substituted arylene alkynylene macrocycles.[S1] Nevertheless, despite a variation of the STM image contrast within the *hexagonal* nanopores (cf. region 1 in Figures S2 and S3), we cannot attribute a specific orientation of OA solvent molecules. This can be most probably attributed to a 2D diffusion of solvent molecules, and/or a mismatch of a solvent cluster of specific size relative to the pore size and geometry. Although we refrain from an overinterpretation, and point out that we are unable to assign solvent molecules to the intrapore contrast variation, we provide a hypothetical model for 12 intercalated OA molecules in a hexagonal nanopore of **1a**. The central region of Figure S5a shows that the ordered intercalation of 6 OA dimers (marked in green) is nominally possible, when the C1–C2 bond of every other OA molecule is rotated by 180°. However, there is no experimental indication on the formation of this exact ordered structure.

Another aspect deals with the chirality of the nanopatterns. In the previous text, we have discussed patterns in which the innermost hexadecyloxy side chains point into clockwise direction as seen from the hexagonal nanopores (cf. region 1 in Figures S2 and S3 as well as Figures S4a-d and S5a). Figure S5b shows a high resolution STM image of **1a** in the same packing as previously described, however in its enantiomeric counterpart. Here, the innermost chains with respect to the hexagonal nanopore are oriented in a counterclockwise fashion. This shows the (obvious) fact, that both enantiomeric packings are observed.

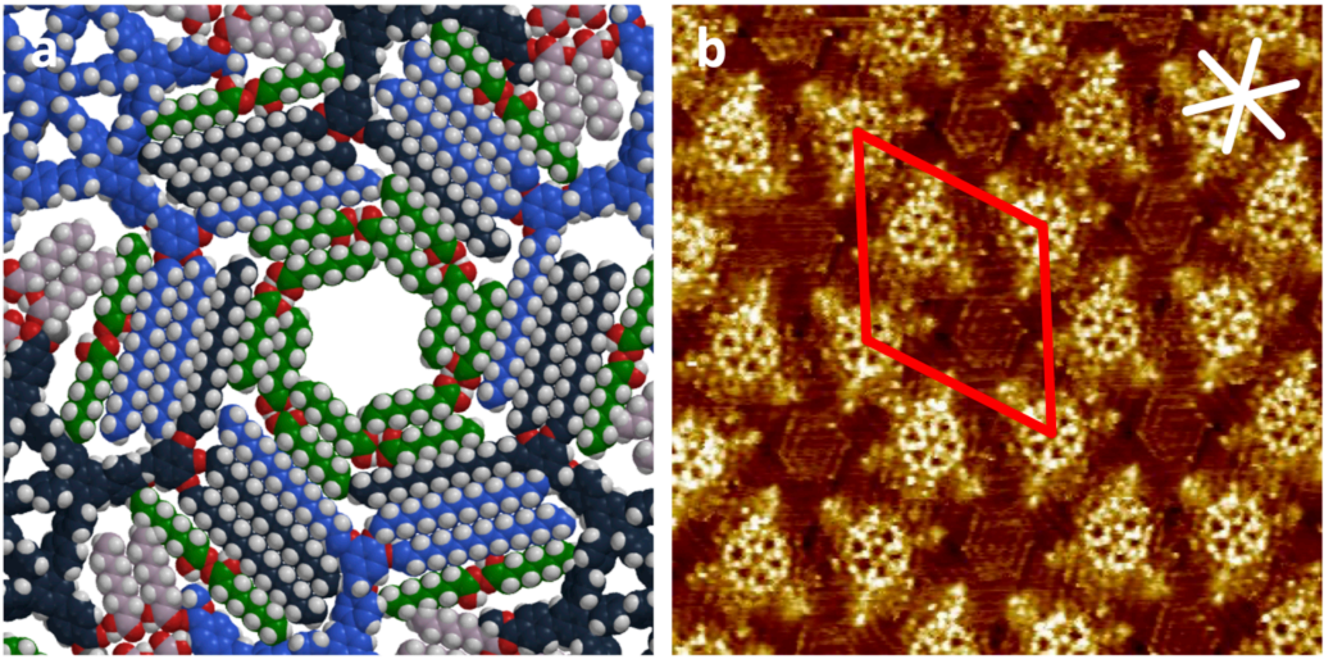

Figure S5. (a) Hypothetical supramolecular packing model of **1a** with additional OA molecules in the central type 1 (hexagonal) nanopore oriented along the HOPG main axis directions, highlighted in green color. (Note, that the model also includes OA molecules in type 2 nanopores, shown in green and pale purple color, which are visible by STM and were previously discussed in Figure S4c and d). (b) High-resolution STM image of **1a** at the solid/liquid interface of HOPG and a solution of **1a** in OA (25.5 × 25.5 nm^2, c = 3 × 10^{-6} M, thermally annealed for 20 s at 100 °C, V_S = –1.6 V, I_t = 20 pA). Red and white lines indicate the unit cell and the HOPG main axis directions, respectively.

2.1.2 *Monolayers of **1a** using PHO as a solvent*

As described previously, **1a** forms SAMs at the solid/liquid interface of HOPG and a solution using OA as a solvent (Figures 2a, e, i, and p (Main Text) as well as Figures S1-S5). In addition, we were interested to what extent nanopatterns could be observed in less polar solvents. This information will be particularly relevant for a control experiment that will be performed using carboxylic acids **1b** (cf. chapter 2.2, Figure S10).

Figure S6 shows STM images of **1a** acquired at the solid/liquid interface of the compound dissolved in 1-phenyloctane (PHO), a nonpolar aprotic solvent. A nanopattern with alike packing ($a = b = (8.2 \pm 0.2$ nm), $\gamma(a,b) = (60 \pm 2)°$) as observed at the interface of **1a** in OA is found. Note that despite several STM tips and samples were used, the image resolution was a bit lower as compared to the STM images obtained for **1a** using OA as solvent.

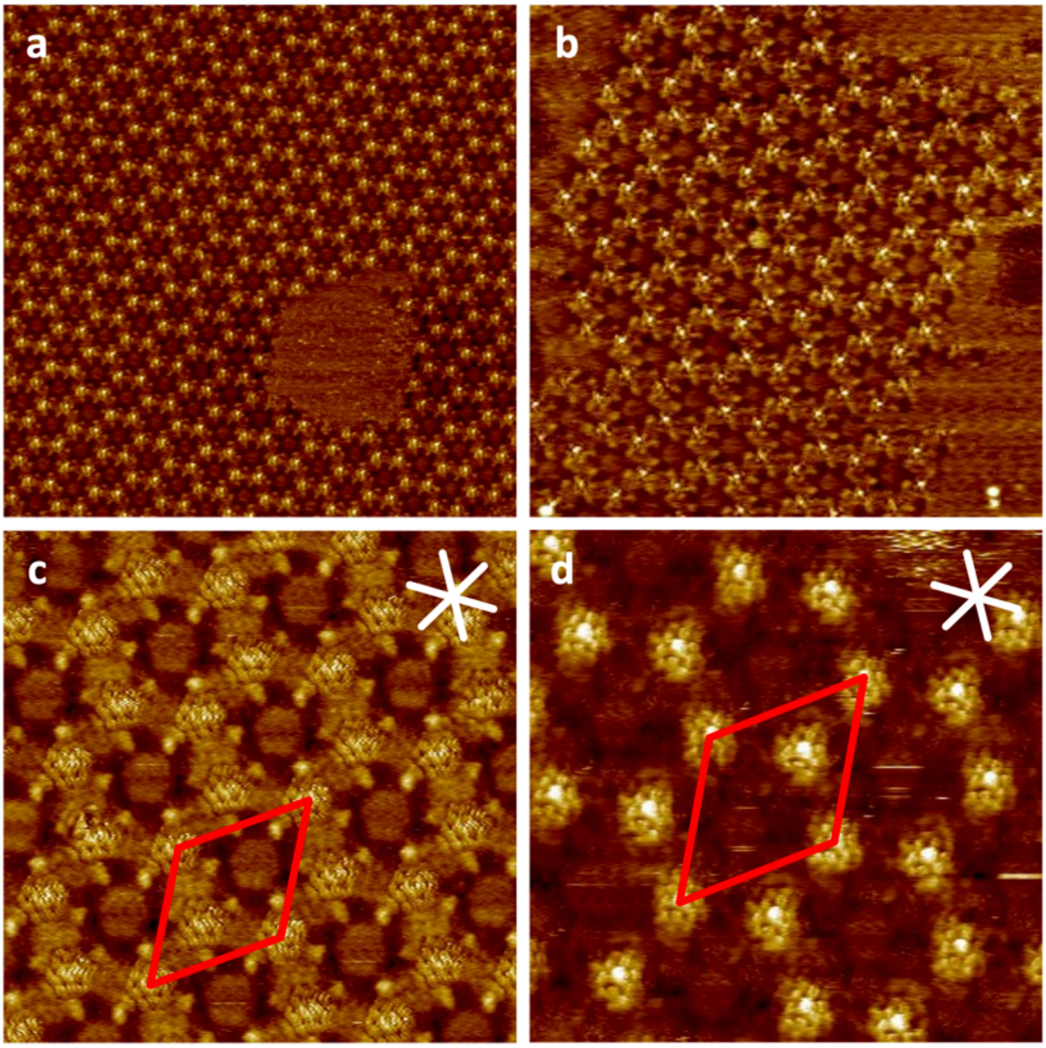

Figure S6. STM images of **1a** at the solid/liquid interface of HOPG and **1a** in PHO (a: 125 × 125 nm^2 (internal scanner calibration), V_S = -0.8 V, I_t = 30 pA; b: 80.7 × 80.7 nm^2 (internal scanner calibration), V_S = –0.8 V, I_t = 35 pA; c: 30.0 × 30.0 nm^2, V_S = –0.65 V, I_t = 2 pA; d: 25.5 × 25.5 nm^2, V_S = –0.8 V, I_t = 35 pA; all images: $c = 1 \times 10^{-6}$ M, thermally annealed for 20 s at 80 °C). Red and white lines indicate the unit cell and the HOPG main axis directions, respectively.

2.2 Additional STM images of **1b**

*2.2.1 Monolayers of **1b** using OA as a solvent*

SAMs of **1b** at the solid/liquid interface of HOPG and a solution of **1b** in OA are investigated by STM. Figure S7 is an enlarged version of Figure S2 (Main Text), showing a monolayer of **1b** with additional assignments. The exact same packing (and the symmetry of the contrast features resembling the molecular structures) as for **1a** is observed for **1b** ($a = b = (8.2 \pm 0.2$ nm), $\gamma(a,b) = (60 \pm 2)°$). Again, the surface areas 1 and 2, which are marked by white dashed lines in Figure S7, indicate the intermolecular nanopores of different sizes and shapes, as already described for **1a**.

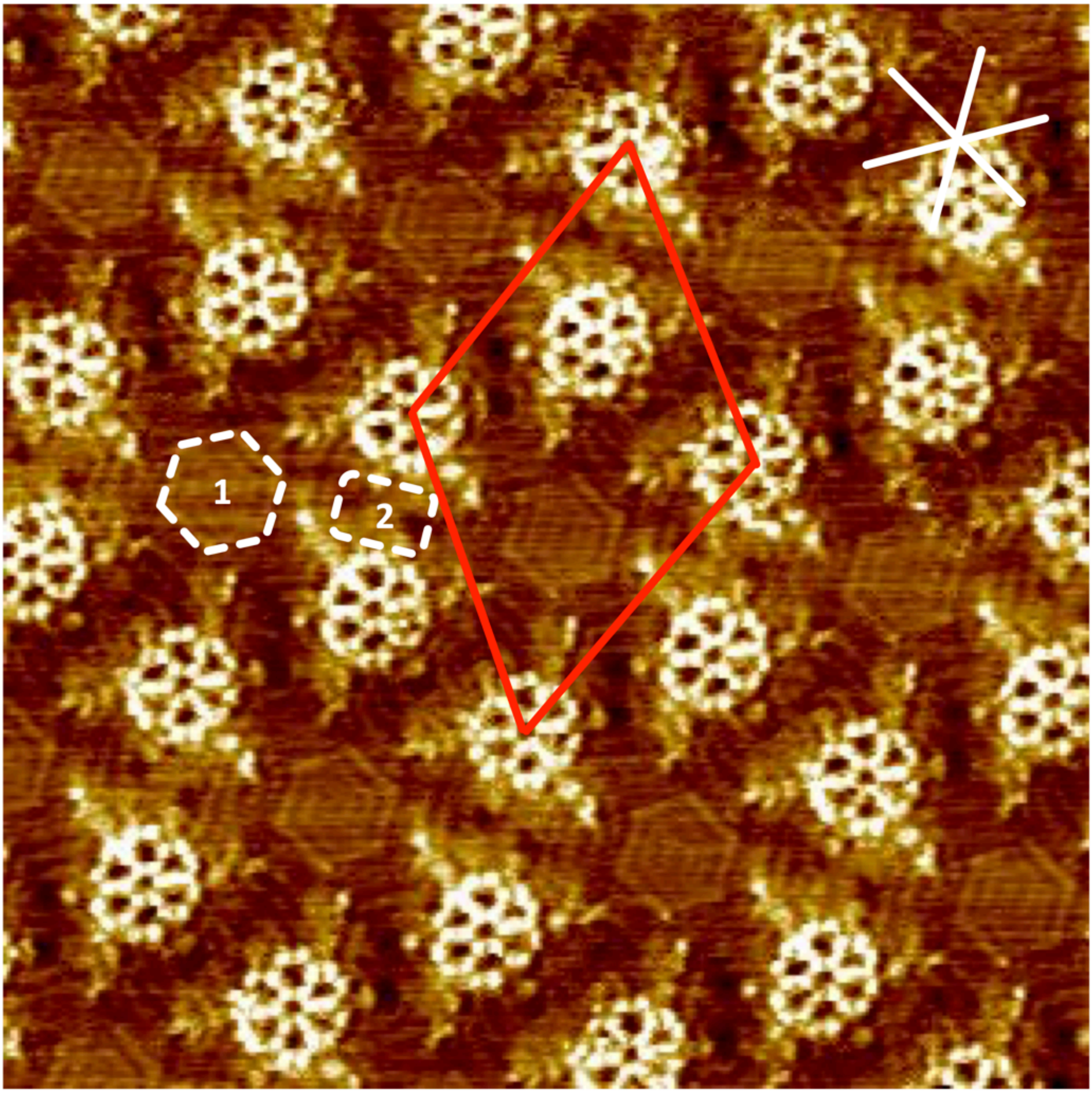

Figure S7. High-resolution STM image of **1b** at the solid/liquid interface of HOPG and a solution of **1b** in OA (25.2×25.2 nm^2, $c = 1 \times 10^{-6}$ M, thermally annealed for 20 s at 100 °C, $V_S = -1.6$ V, $I_t = 30$ pA). Red and white lines indicate the unit cell and the HOPG main axis directions, respectively.

Figures S8a and b are enlarged versions of Figures 2f and k (Main Text), showing supramolecular and schematic models of the nanopattern of **1b**. c_A and c_B in Figure S7c highlight different backbone orientations marked in light and dark blue color, respectively.

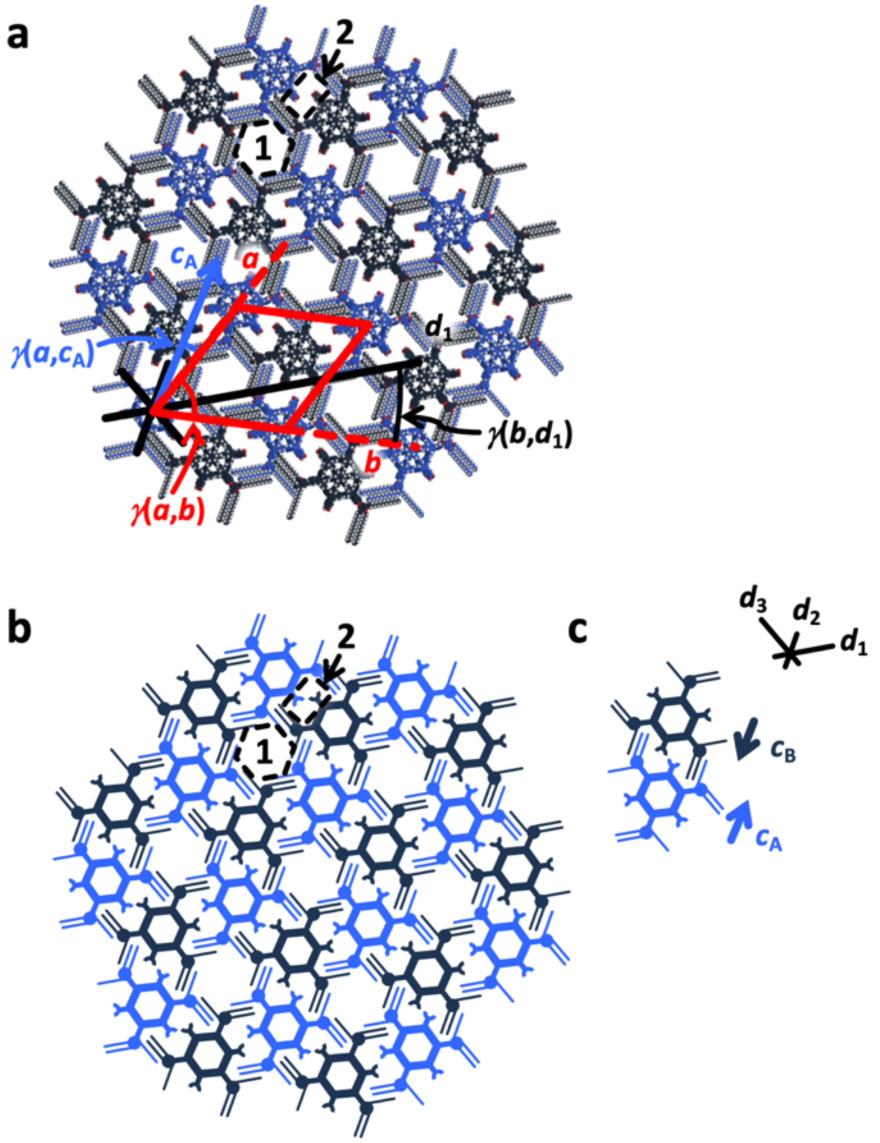

Figure S8. (a) Supramolecular and (b)-(c) schematic models of the nanopatterns of **1b**. The red lines indicate the indexed unit cell, $a = b = (8.2 \pm 0.2\ \text{nm})$, $\gamma(a,b) = (60 \pm 2)°$. Molecules with two different orientations, c_A and c_B, are highlighted in light and dark blue color. The backbones are oriented with $\gamma(a,c_A) = (16 \pm 3)°$. All hexadecyloxy side chains are oriented along the HOPG main axis directions d_1 to d_3. The nanopattern is oriented relative to d_1 such that $\gamma(b,d_1) = (18 \pm 3)°$. Again, 1 and 2 indicate two different types of intermolecular nanopores.

As seen in the high resolution STM image in Figure S9, the acid groups of two different MSWs **1b** point past each other and do not interact with each other *via* hydrogen bonding. Arrow D_3 marks the distance between the carbon atoms of two adjacent carboxylic acid groups with D_3 = (1.5 ± 0.1) nm which is the same distance as for the two benzoic acid methyl ester groups of **1a** (Figure S4). However, we highly assume that the benzoic acid groups are paired with OA solvent molecules (see Main Text). In addition, the solvent molecules in the image of **1b** are not clearly resolved by STM as it was the case for **1a** in the smaller (type 2) nanopores (see Figures S2, S3, and S4). This is probably a result of their hydrogen bond interaction with the benzoic acid groups and a therefore different as well as less dense packing, allowing for some mobility. However, as the OA molecules remain invisible, we refrain from including them into the supramolecular model shown in Figure S8b.

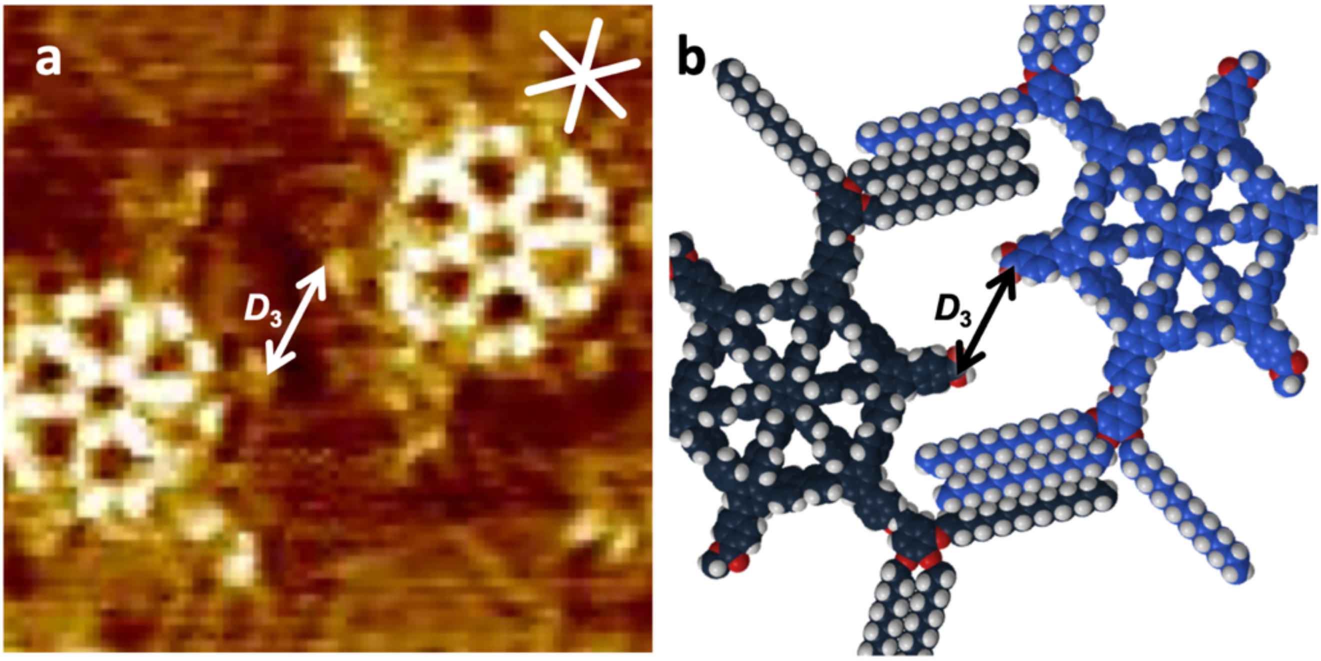

Figure S9. (a) High-resolution STM image and (b) supramolecular model of **1b** at the solid/liquid interface of HOPG and a solution of **1b** in OA (7.3 × 7.3 nm^2, $c = 1 \times 10^{-6}$ M, thermally annealed for 20 s at 100 °C, V_S = –1.6 V, I_t = 30 pA). The white lines indicate the HOPG main axis directions.

*2.2.2 Monolayers of **1b** using PHO as a solvent*

As described previously, **1b** forms SAMs at the solid/liquid interface of HOPG and a solution using OA as a solvent (Figures 2b, f, k, and q (Main Text) as well as Figures S7-S9). In addition, alike for **1a** (cf. Figure S6) we were interested to what extent nanopatterns of **1b** could be observed in less polar solvents. Figures S10a and b show STM images of **1b** acquired at the solid/liquid interface of the compound dissolved in 1-phenyloctane (PHO). In the overview image (Figure S10a), a highly disordered monolayer of **1b** is observed, despite some tendency to form honeycomb assemblies is found (*e.g.* in the lower right image region). In the detail STM image (Figure S10b), a disordered periodic honey-comb like assembly is observed. However, the molecules have irregular distances and alignments. The poor image quality is attributed to the less robust packing of **1b** at the interface of HOPG and **1b** in PHO due to disorder. It remains speculative, whether the dimerization of the carboxylic acid groups of some molecules leads to a breakdown of the alkoxy chain interdigitation motifs, and therefore propagation of the disorder to the residual packing. (Note that despite several STM tips and samples were used, no better resolved images could be acquired.)

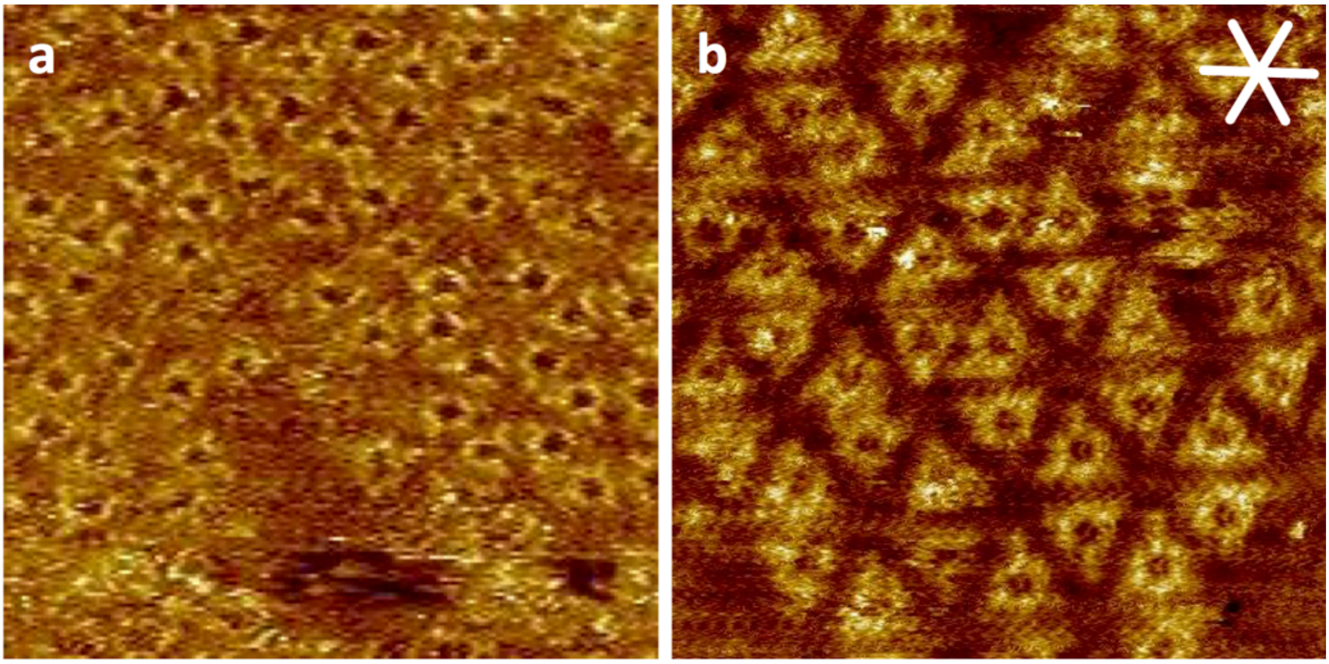

Figure S10. STM images of **1b** at the solid/liquid interface of HOPG and **1b** in PHO (a: 50×50 nm^2 (internal scanner calibration), V_S = –1.1 V, I_t = 26 pA; b: 35.8 × 35.8 nm^2, V_S = –1.4 V, I_t = 22 pA; all images: c = 1 × 10^{-6} M, thermally annealed for 20 s at 80 °C). White lines indicate the HOPG main axis directions.

2.3 Additional STM images of **2a** using OA as solvent

Figure S11 shows an overview STM image of **2a**. The area is covered by different domains (A-F). The molecules in domains A-D adopt highly ordered 2D crystalline packings consistent with the model proposed in the Main Text (see Figure S13 for an enlarged copy), and unit cells are marked in red color. Domains A and B share the same orientation of the packing, but are slightly shifted along the dotted white line between A and B (which marks a line defect of the 2D crystal). With respect to the chiral packing motif of the crystalline domains A and B, the domains C and D represent a mirrored packing orientation (or enantiomer), and no shared domain boundary is formed between B and C, or A and D, respectively. However, we interpret the regions E and F, that are formed between these pairs of domains of different orientations and in which the molecules adopt a reduced order, as an energetic compromise. In addition, a large solvent covered region in the center of the image is observed.

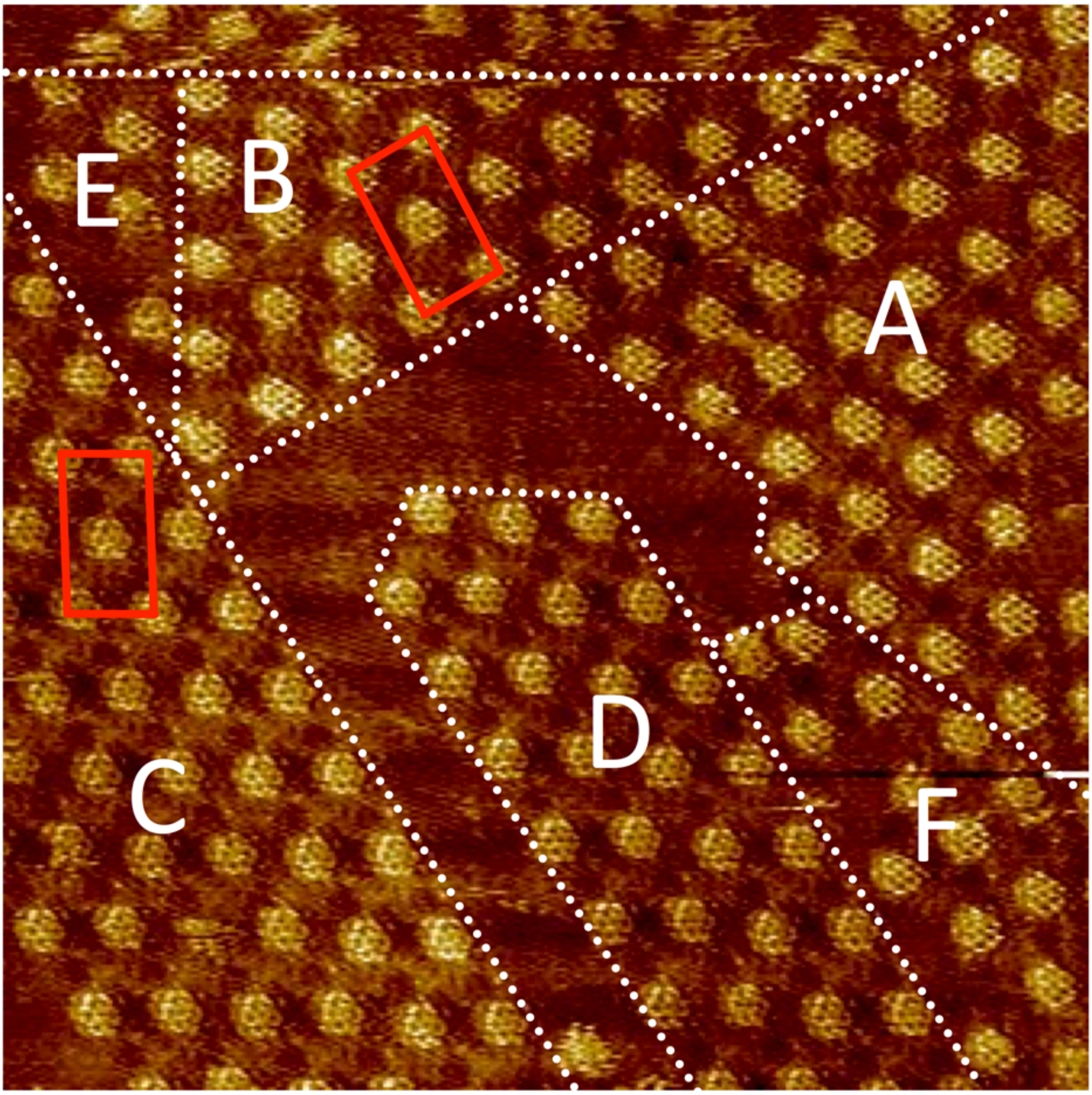

Figure S11. Overview STM image of **2a** at the solid/liquid interface of HOPG and a solution of **2a** in OA (85.5×85.5 nm^2, internal scanner calibration, $c = 1 \times 10^{-6}$ M, thermally annealed for 20 s at 100 °C, $V_S = -0.8$ V, $I_t = 18$ pA). Domain boundaries are indicated by white dotted lines, indexed unit cells for domains B and C with enantiomeric packings are indicated by red lines.

Figure S12 shows an enlarged copy of Figure 2c (Main Text), which is a high-resolution STM image of **2a** at the solid/liquid interface of HOPG and a solution of **2a** in OA. Additionally, enlarged versions of the corresponding supramolecular and schematic models in Figures 2g, l, and m (Main Text) are shown in Figure S13. At first glance, being misguided by the brightly appearing MSW backbones, one might erroneously assume a hexagonal packing of the MSWs. However, we must first consider the symmetry of **2a** before discussing the symmetry of the nanopattern. While the three trihexadecyloxy substituted benzyloxy groups per molecule of **2a** appear rather dark here, we can clearly identify the positions of their aliphatic and aromatic moieties in the high-resolution STM image. The phenyl groups carrying the alkoxylated benzyloxy groups are indicated by white circles in Figure S12. Therefore, the positions of their substitution at the MSW backbones clearly confirm the C_s symmetry of **2a**. To the nanopattern, an oblique (and nearly rectangular) unit cell with parameters a = (9.3 ± 0.2) nm, b = (5.7 ± 0.2) nm, $\gamma(a,b)$ = (88 ± 2)° containing two MSWs is indexed. These two molecules adopt the relative backbone orientations c_A and c_B of $\gamma(c_A, c_B)$ = 180° (Figure S11c), and they are conformers with respect to their alkoxy side chain orientations.

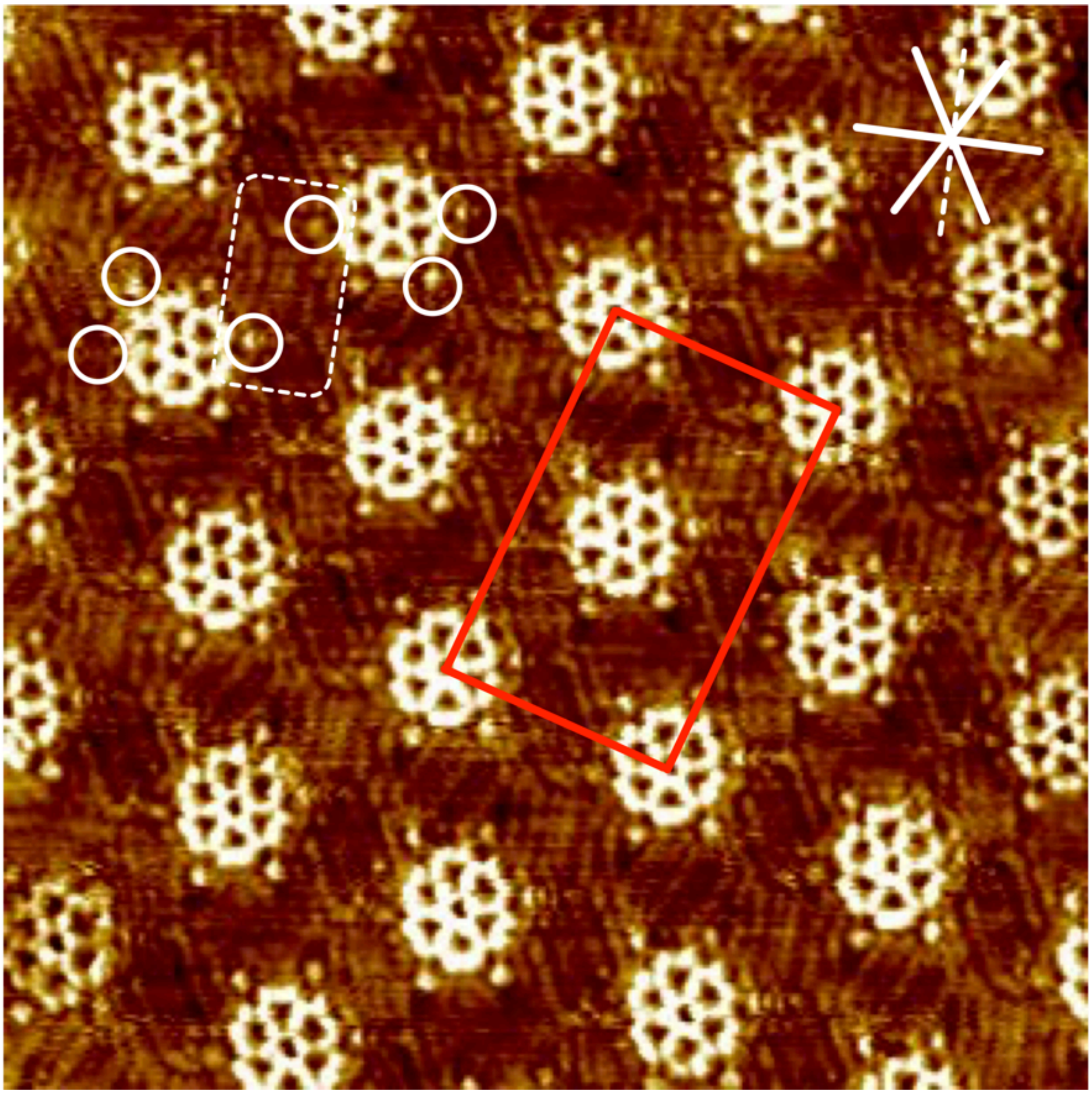

Figure S12. High-resolution STM image of **2a** at the solid/liquid interface of HOPG and a solution of **2a** in OA (25.2 × 25.2 nm^2, c = 1 × 10^{-6} M, thermally annealed for 20 s at 100 °C, V_S = –1.1 V, I_t = 11 pA). Red and white lines indicate the indexed unit cell and HOPG main axis directions respectively.

In addition, we shall now discuss the packing of the alkoxy side chains in detail. The main axis directions d_1, d_2, and d_3 of the graphite substrate are shown as white and black solid lines in Figures S12 and S13, respectively. 14 hexadecyloxy side chains of each two molecules with orientations c_A and c_B adsorb along them and are indicated as bright and dark blue lines in the schematic models in Figures S13b and c. We attribute the overall packing geometry to the energetic contribution of the interaction of these 14 chains with the HOPG substrate along d_1, d_2, and d_3. As a consequence, four additional hexadecyloxy side chains per pair of molecules, *e.g.* in the region marked by the white (black) dashed box in Figures S12 and S13a and shown in purple color in Figure S13, cannot adsorb along one of the preferred HOPG main axis directions, $d_{1\text{-}3}$ due to steric reasons. Instead, they adsorb along one of the three armchair directions, d_4, marked by the dashed black line in Figure S13a.

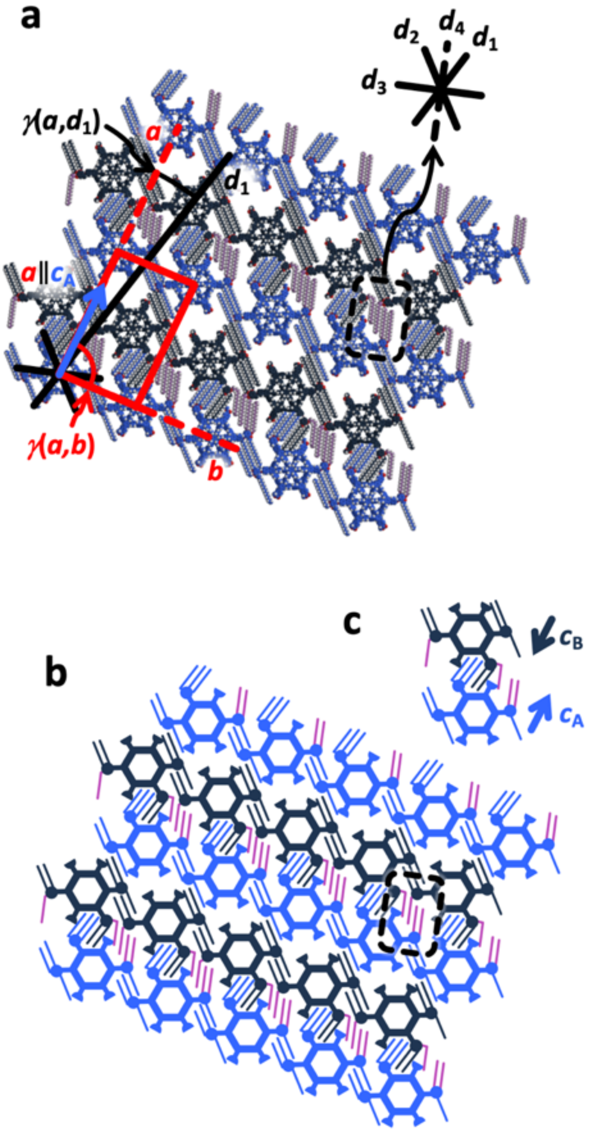

Figure S13. (a) Supramolecular and (b)–(c) schematic models of the nanopatterns of **2a**. The red lines indicate the indexed unit cell, a = (9.3 ± 0.2 nm), b = (5.7 ± 0.2 nm), $\gamma(a,b)$ = (88 ± 2)°. Molecules with two different orientations, c_A and c_B, are highlighted in light and dark blue color. The backbones are oriented with $c_A \parallel a$. 14 of 18 hexadecyloxy side chains in (a), marked in light and dark blue color, are oriented along the HOPG main axis (or zig-zag) directions of the HOPG susbtrate that are indicated by solid black lines in (a). The thin light and dark blue lines in (b) and (c) symbolize these. 4 hexadecyloxy side chains of each pair of molecules in (a), marked in purple color, are oriented along the armchair directions of the HOPG substrate, d_4, indicated by the dashed black line in the upper part of (a). The thin purple lines in (b) and (c) symbolize these. The nanopattern is oriented relative to d_1 such that $\gamma(a,d_1)$ = (12 ± 2)°.

Figure S14 shows another high-resolution STM image of **2a**. The white circles indicate the aromatic units of the trialkoxybenzyloxy building blocks (that appear here more brightly as compared to Figure S12), proving again the 1,2,4-substitution pattern and C_S symmetry of the MSW. Moreover, the adsorption pattern of the hexadecyloxy side chains is well resolved. In the region marked by the dashed rectangular box, arrows 1 to 4 indicate the four alkoxy side chains that that were marked in purple color in Figure S13 and are oriented along one of the armchair directions, d_4, of the substrate. The latter is indicated by the dashed white line in the upper right corner of Figure S14.

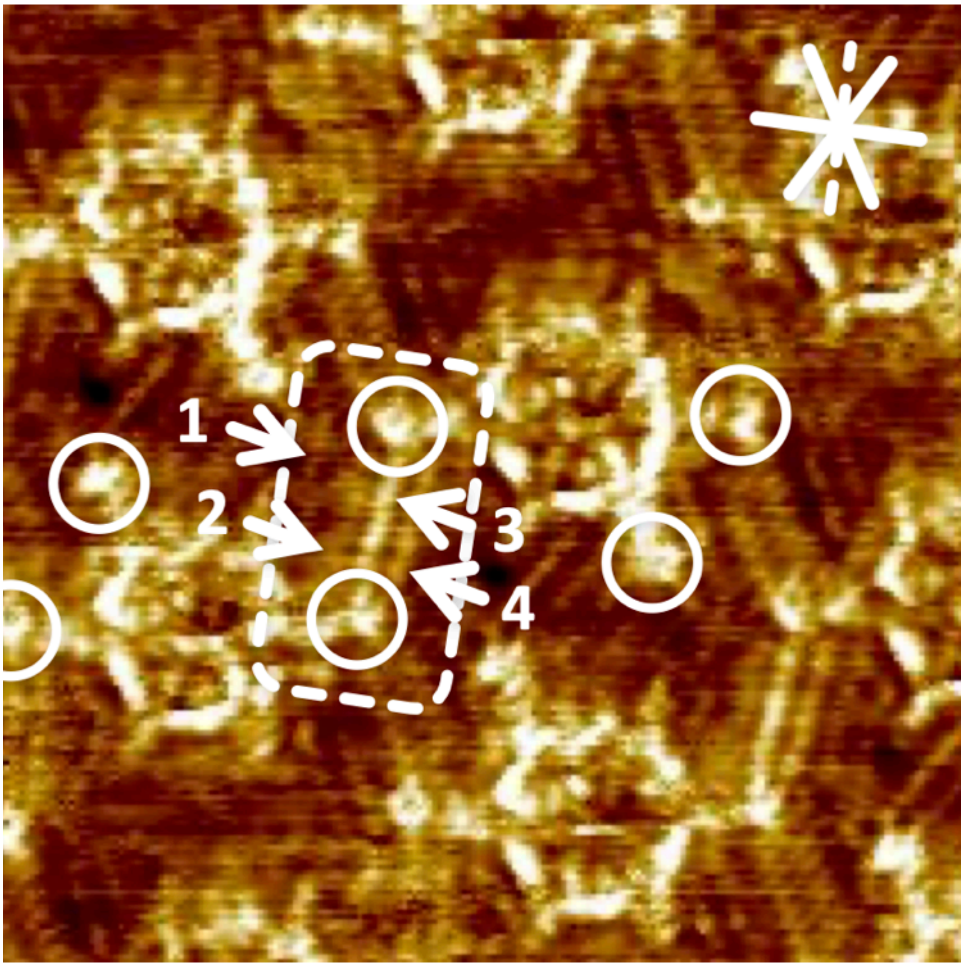

Figure S14. High-resolution STM image of **2a** at the solid/liquid interface of HOPG and a solution of **2a** in OA (10.5×10.5 nm^2, $c = 1 \times 10^{-6}$ M, thermally annealed for 20 s at 100 °C, $V_S = -1.6$ V, $I_t = 20$ pA). The asterisk of solid white lines indicates the HOPG main axis directions, the dashed line in the asterisk indicates the armchair direction along which four hexadecyloxy side chains per pair of molecules adsorb.

As shown in the molecular and schematic models in Figures S13a and b, the backbones and hexadecyloxy side chains of the MSWs cannot pack densely to cover the whole HOPG surface. As a consequence, the periphery of these backbones and side chains is filled with OA (solvent) molecules, and some of them are well resolved by STM. Of particular interest is, whether the unexpected orientation of the four hexadecyloxy side chains per pair of molecules is significantly stabilized by intercalating dimers of solvent molecules.

Figure S15a shows a high-resolution STM image of **2a** (which is a detailed section taken from Figure S12 and Figure 2c (Main Text)), and Figure S15b shows the corresponding transcribed supramolecular model. Each unique solvent dimer that is clearly observed in the STM image (cf. arrows 1 to 7 in Figure S15a) is added to the model (arrows 8 to 14 in Figure S15b). Two of these dimers (arrows 4 and 5 in Figure S15a, corresponding to 11 and 12 and marked in pale purple color in Figure S15b) are oriented along the HOPG armchair direction given by

the dashed line in the upper right corner of Figure S15a, and are aligned next to the four hexadecyloxy side chains that were discussed in Figure S14 and marked in purple color in Figures S13 and S15b and c. The remaining dimers (cf. arrows 1 to 3 as well as 6 and 7 in Figure S15a, corresponding to arrows 8 to 10 as well as 13 and 14 in Figure S15b, and marked in green color in Figures S15b and c), are aligned along one of the HOPG main axis (or zig-zag) directions. All these dimers are in close proximity to neighboring alkoxy chains and are assumed to stabilize the overall packing, as uncovered regions on the HOPG surface are avoided. Moreover, the fact that they can be observed by STM shows that they are immobile on the timescale of measurement. The remaining regions (*e.g.* the region between arrows 1 and 2 or 8 and 9, respectively) are assumed to be covered by solvent molecules as well, as it is shown exemplarily in a hypothetical model involving the three green additional OA dimers in Figure S15c. However, we attribute their invisibility in the STM experiment to some residual degrees of freedom, which allows such a high mobility that they cannot be observed on the timescale of the comparably slow STM experiment.

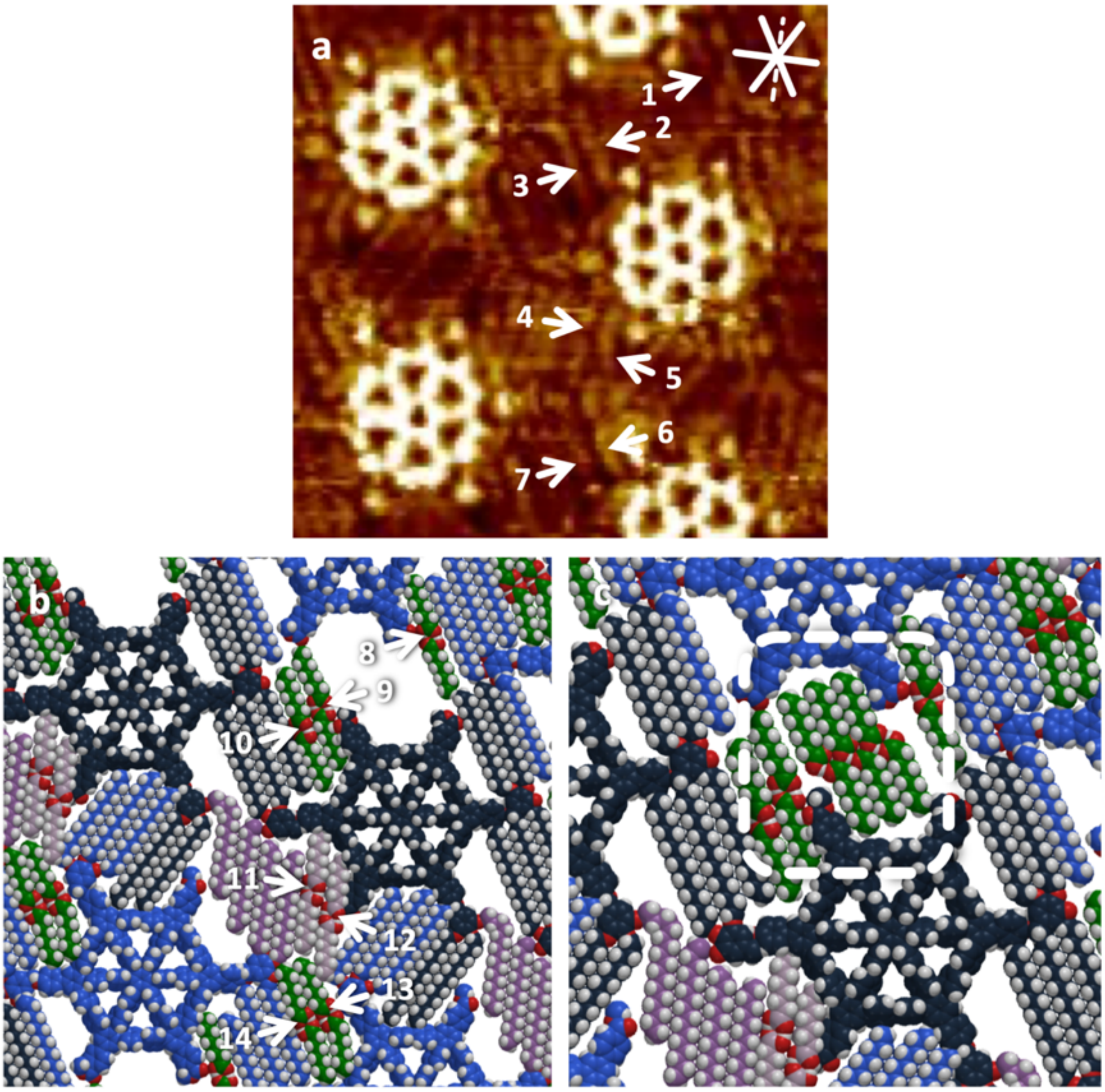

Figure S15. (a) Detailed image section taken from Figure S12 and Figure 2c (Main Text) and (b) supramolecular model of **2a** at the solid/liquid interface of HOPG and a solution of **2a** in OA (10.2 × 10.2 nm^2, $c = 1 \times 10^{-6}$ M, thermally annealed for 20 s at 100 °C, V_S = –1.1 V, I_t = 11 pA). Red and white lines indicate the indexed unit cell and HOPG main axis directions respectively. Solvent molecules that are clearly observed in the STM image are depicted in green color. (c) Additional supramolecular packing model of **2a**, in which three hypothetical (but not by STM observable) additional OA dimers are included in green color. Again, note that we have no indication on the packing the latter from the experiment.

2.4 Additional STM images of **2b** using OA as solvent

Figure S16 is an enlarged version of Figure 2d (Main Text) with additional assignment and shows an STM image of **2b**. Its supramolecular nanopattern could only be less good resolved as compared to the other compounds, and Figure S16 shows the best STM image that could be obtained despite different samples were prepared and several tips, samples and imaging conditions were used. Nevertheless, from the STM data we could clearly interpret the supramolecular packing in the white dashed region, and the corresponding transcript is shown in Figure S17 as a space-filling model. The distances between two different rows of molecules (indicated by dashed yellow lines in Figure S16) are given as d_a = (5.1 ± 0.2) nm and d_b = (5.8 ± 0.2) nm. We attribute these different packing distances to different alkoxy side chain interaction schemes, and the packing will be further discussed in Figure S17.

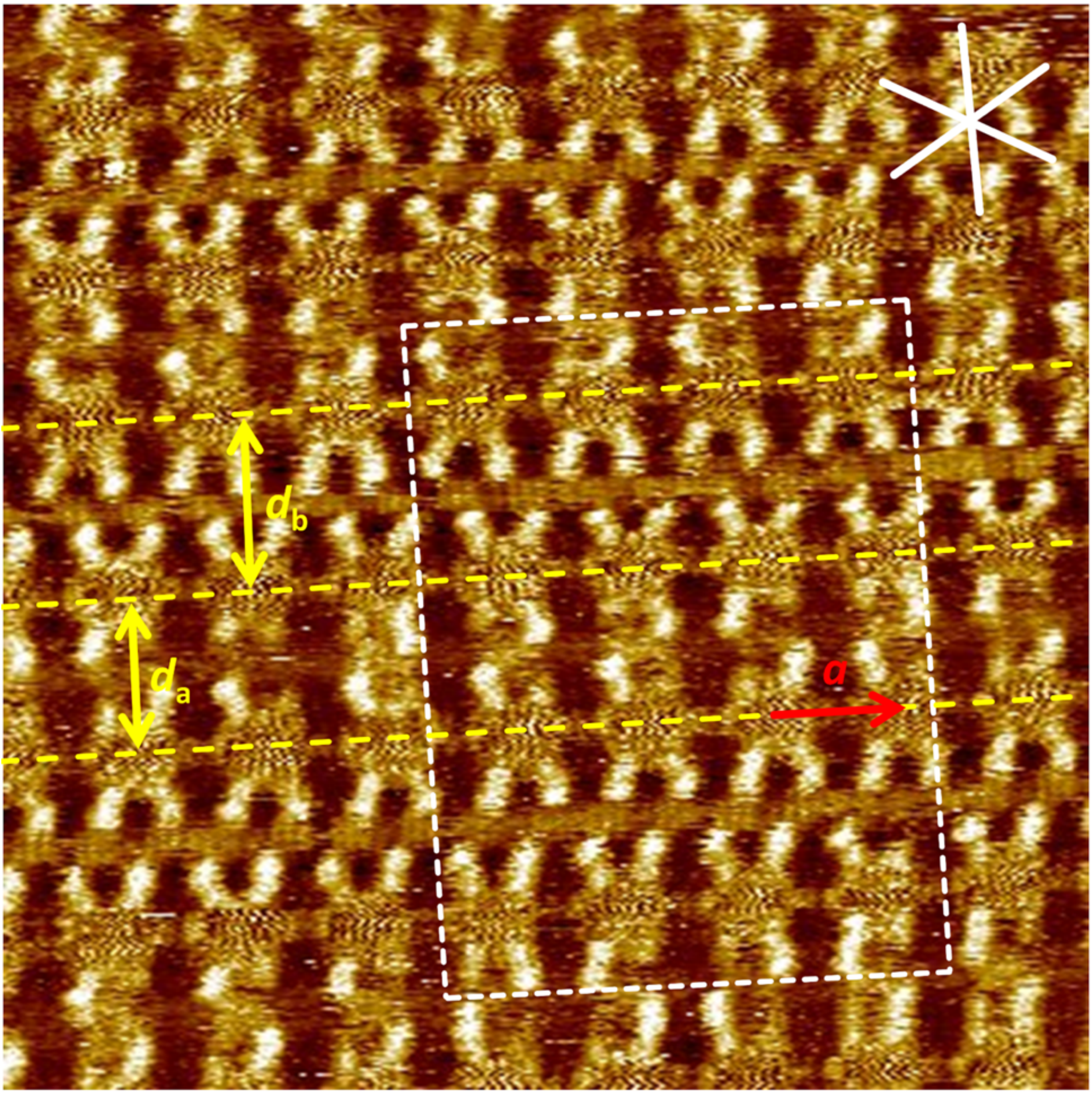

Figure S16. High-resolution STM image of **2b** at the solid/liquid interface of HOPG and a solution of **2b** in OA (37.2 × 37.2 nm^2, c = 10^{-6} M, thermally annealed for 20 s at 110 °C, V_S = –0.9 V, I_t = 22 pA). The white asterisk indicates the HOPG main axis directions. Distances d_a and d_b between two different rows of molecules (cf. yellow dashed lines) are marked by yellow arrows.

Figures S17a, b, and c show supramolecular and schematic models of the nanopattern formed by **2b**. The substitution of the backbone of **2b** by carboxylic acid functions in 1 and 4 positions allows a packing of the molecules in one-dimensional rows (along the dashed yellow lines in Figure S16). In Figures S16 and S17a, the unit vector a = (4.4 ± 0.2) nm indexed to this packing is given as red arrow, however neglecting the reduction of the symmetry of the packing that is induced by the two different orientations of the backbones c_A and c_A' (or c_B and c_B', cf. Figure S17c. The carboxylic acid groups at the 1 and 4 positions of adjacent molecules along a in Figure S17a and b (and again, the yellow dashed lines in Figure S16) interact under formation of cyclic carboxylic acid dimer motifs (Figure S17d). These are well known for carboxylic acids and have also been described on solid surfaces for *e.g.* trimesic acid (benzene 1,3,5-tricarboxylic acid) and its larger homologues (see Main Text). However, the acid groups in the 2-positions of adjacent molecules of **2b** do not interact with each other in this arrangement, which is in contrast to trimellitic acid (benzene 1,2,4-tricarboxylic acid, see Main Text).

Two out of three hexadecyloxy side chains of each trialkoxybenzyloxy unit in the region marked by arrows 1 (Figure S17a) and 3 (Figure S17b) are adsorbed along one of the HOPG main axis directions, whereas the third side chain remains invisible by STM and is either randomly adsorbed along HOPG or points towards the solution phase. The alignment of the (two physisorbed) alkoxy chains (per molecule, as marked by arrows 1 and 3) determines the relative orientation of pairs of MSWs (which are defined by the black boxes in Figure S17a). The four different orientations of the MSWs in the packing are additionally assigned as c_a, c_b, c_a', and c_b' (see Figure S17c) and highlighted in lighter and darker colors (*i.e.* light and dark blue or grey) in Figure S17 a to c. However, the molecules in each row can adopt c_a or c_a' randomly, which determines the orientation of adjacent molecules c_b or c_b' (and *vice versa*). These rows of pairs of molecules along the indexed unit vector a (and defined by the black boxes in Figure S17a) are again connected by interdigitation of alkoxy side chains marked by arrows 2 (Figure S17a) and 4 (Figure S17b). These side chains are orthogonally aligned with respect to the unit vector a and are aligned along one of the main axis directions, d_1, of the underlying HOPG template indicated by the black asterisk. In this arrangement, five out of six alkoxy side chains per molecule are densely adsorbed to the graphite surface, which is observed by STM (arrow 2 in Figure S18h, and Figure S18i) and consistent with the theoretical interchain distance of 4.26 Å (cf. Main Text), although it remains unresolvable to which exact trialkoxybenzyloxy unit they are assigned. The remaining (sixth) chain points due towards the solution phase due to steric reasons and does not contribute to the nanopattern.

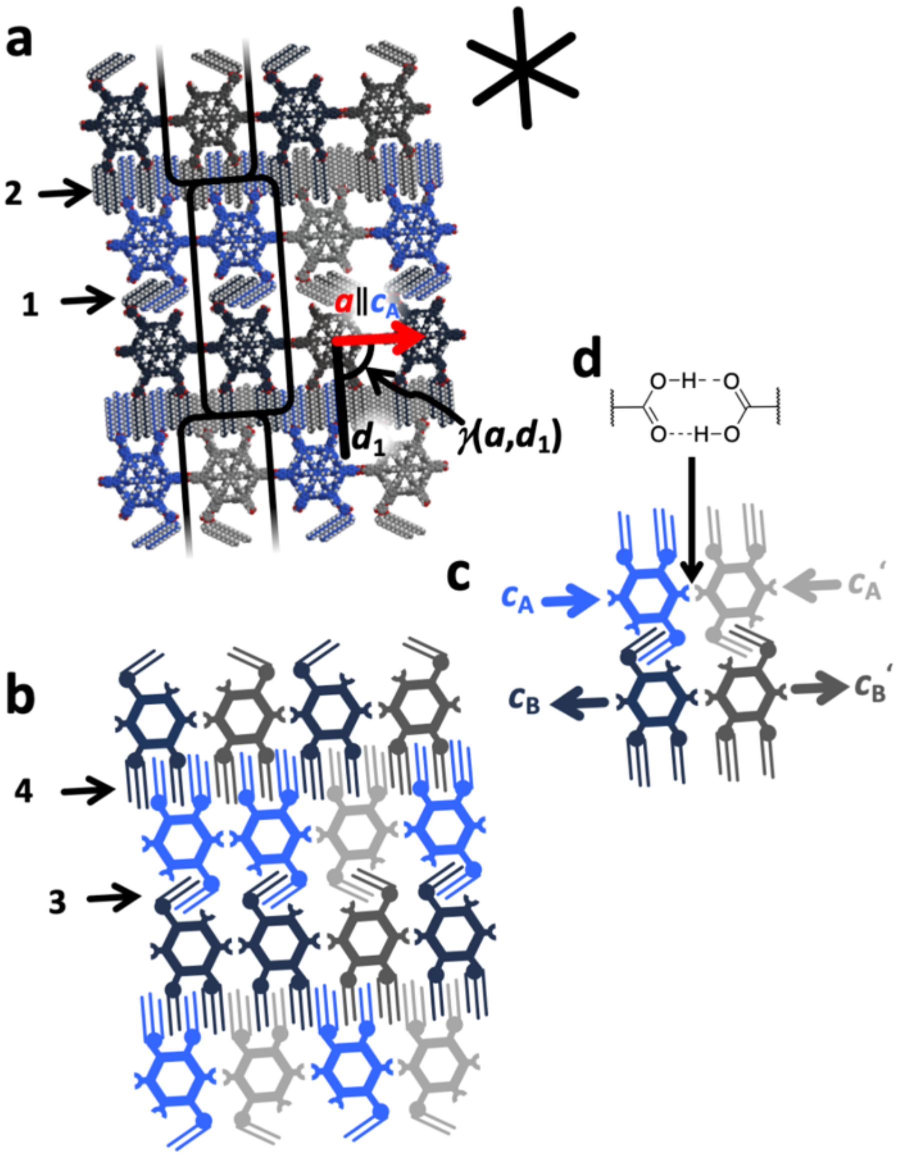

Figure S17. (a) Supramolecular and (b)–(c) schematic models of the nanopatterns of **2b**, and (d) chemical structure of the cyclic carboxylic acid dimers. The red arrow in (a) indicates the indexed unit vector a = (4.4 ± 0.2) nm, neglecting the exact orientation of the MSW backbones c_A, c_A', c_B, and c_B' indicated by light and dark blue and grey colors and arrows in (c). Moreover, the MSW backbone orientation c_A is oriented in parallel to the indexed unit vector a (c_A || a), which is in turn oriented orthogonal ($\gamma(a,d_1)$ = (90 ± 2)°) relative to the HOPG main axis direction d_1 indicated by the black solid line in (a). Alkoxy chain packings marked by arrows 1 in (a) and 3 in (b) determine the relative orientation of pairs of MSWs that are defined by the black boxes in (a). Arrows 2 in (a) and 4 in (b) indicated regions where five out of six hexadecyloxy side chains are densely packed and oriented along d_1 and interdigitate intermolecularly.

Figure S16a shows a reprint of the high resolution STM image of **2b** shown in Figure 2s (Main Text) in which a phenomenon occurring at domain boundaries is observed: Three spoked wheels form a triangular motif due to hydrogen bonding between a total of six carboxylic acid groups, so that the packing motif as shown in Figure S16b and c is formed. Additional STM images of **2b** are given in Figures S16d-j.

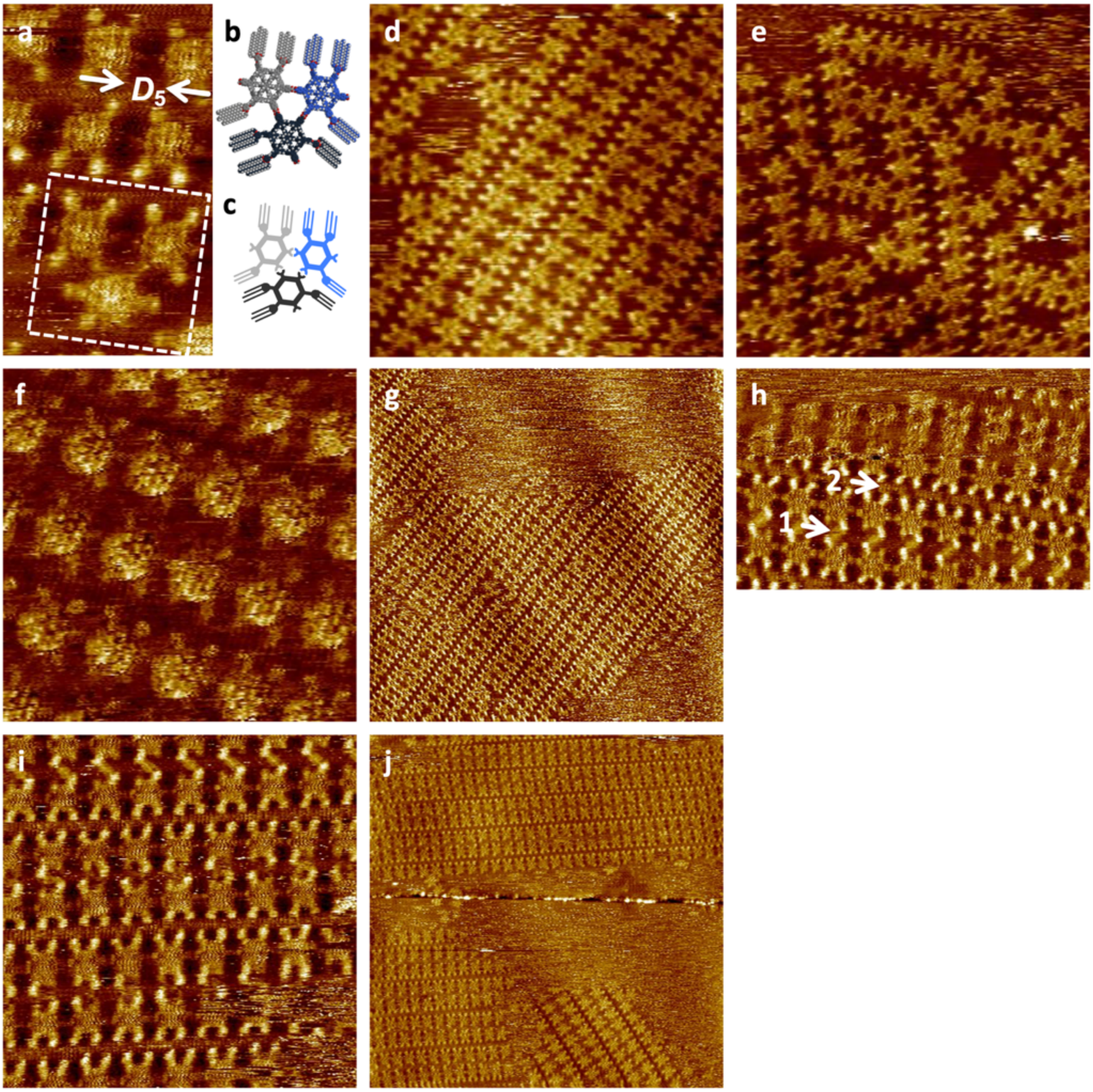

Figure S18. (a) High-resolution STM image of **2b** at the solid/liquid interface of HOPG and a solution of **2b** in OA (10.0×16.9 nm^2, $V_S = -0.8$ V, $I_t = 15$ pA, $c = 1 \times 10^{-6}$ M, thermally annealed for 20 s at 110 °C), (b) supramolecular, and (c) schematic model of the timeric cluster of **2b** marked by the dashed box in (a). (d)–(j) Additional STM images of **2b** at the solution/HOPG interface of **2b** in OA ($c = 1 \times 10^{-6}$ M, (d)–(f) thermally annealed for 20 s at 100 °C, (g)–(j) thermally annealed for 20 s at 110 °C): (d) 61.6×61.6 nm^2, $V_S = -0.9$ V, $I_t = 28$ pA ; (e) 61.1×61.1 nm^2, $V_S = -0.8$ V, $I_t = 39$ pA; (f) 22.1×22.1 nm^2, $V_S = -1.2$ V, $I_t = 32$ pA; (g) 150×150 nm^2, $V_S = -1.0$ V, $I_t = 22$ pA; (h) 41.7×26.0 nm^2, $V_S = -0.8$ V, $I_t = 22$ pA; (i) 39.6×39.6 nm^2, $V_S = -0.8$ V, $I_t = 22$ pA; (j) 150×150 nm^2, $V_S = -0.8$ V, $I_t = 22$ pA. Arrow 1 in (h) highlights the alkoxy chains indicated by arrows 1 and 3 in Figure S17a and b, whereas arrow 2 in (h) highlights the alkoxy chains indicated by arrows 2 and 4 in Figure S17a and b.

Up to now, we have (i) discussed the nanopattern of **2b** in which the carboxylic acids at the 1- and 4-positions of the MSWs interact with those of adjacent molecules to form 1D supramolecular polymers (cf. Figures 2d, h, n, and o (Main Text) and S16 and S17), and (ii) another characteristic structural feature in which the carboxylic acid functions at the 1- and 2-positions of the MSWs interact to form cyclic supramolecular trimers (cf. Figures 2s, t, and u (Main Text) and Figure S18a to c.

Figure S19a shows an additional overview STM image of **2b** with additional features. The image includes binding motifs (i) and (ii) which were previously observed in the aforementioned superstructures. Despite the lack of 2D crystallinity in the given STM image, unexpected structural motifs of increased complexity are found and marked by differently colored polygons superimposed to the STM image, and corresponding supramolecular models of these are shown in Figures S19b, c, and d. Again, all these motifs include intermolecular interactions of carboxylic acid functionalities under formation of cyclic dimers (cf. Figure S17d). However, in all these motifs, different combinations of MSWs interacting *via* carboxylic acids at their 1- and 2- as well as 1- and 4-positions are formed. Another remarkable feature is, that all these are highly symmetric objects (trapezoids, marked in ocher and rosé color) or even are observed as pairs of two enantiomeric objects (rhomboids, marked in bright and dark blue color).

We interpret that behavior as a result of the different angles between the two acid functionalities and the center of each backbone (“opening angles”), which are 60°, 120°, and 180°, based on the symmetry of the backbones (cf. chemical structure in Figure 1 (Main Text)). Moreover, there are (a) aperiodic binding motifs in which either all three or only two carboxylic groups of **2b** can contribute to the intermolecular interactions (*i.e.* the triangular units in Figures S18a to c and S19b to d), and there are (b) periodic binding motifs in which only two carboxylic acid groups interact with those of adjacent molecules (*i.e.* the nanopattern in Figures S16 and S17 as well as the sides of the polygons in Figure S19). The structural properties of the molecules and the allowed binding scenario are reflected in the observed structural motifs, as the corners and sides of the so-formed supramolecular geometric objects coincide with the opening angles of its molecular constituents.

More specifically, the trapezoid shown in Figure S19b, superimposed in rosé color, consists of 11 MSWs whereas the trapezoid shown in Figure S19c, superimposed in ocher color consists of 12 MSWs. The two 2D-enantiomeric rhomboids shown in Figure S19d, superimposed in light and dark blue colors, consist of 10 MSWs each.

We assume, that the STM image shown in Figure S19a shows a metastable packing of **2b**, which has not reached the global thermodynamic minimum. No long-range ordered aggregates are formed, and the observed moieties are dominated by H-bonding between two acid functionalities and less driven by van der Waals interactions.

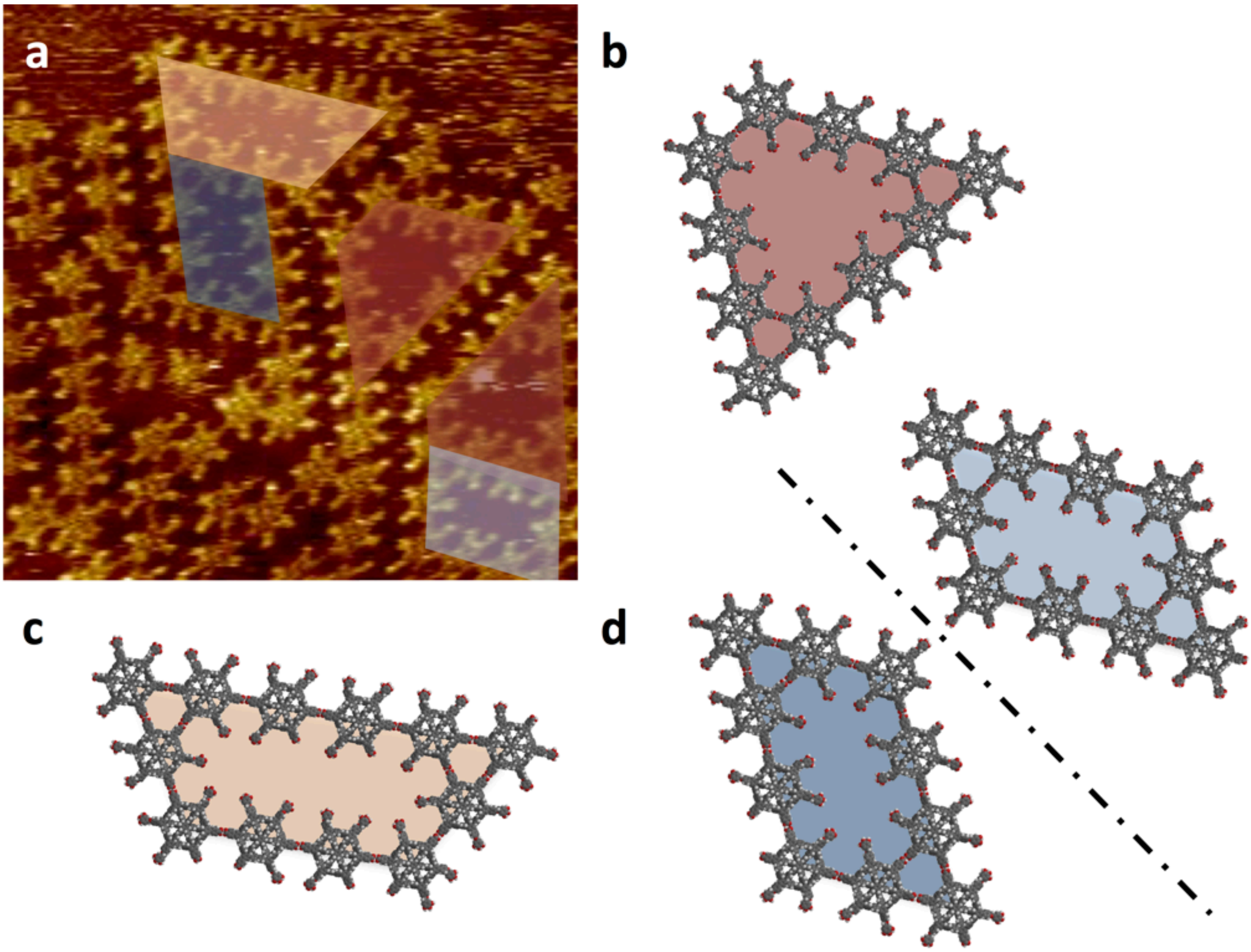

Figure S19. Additional STM image of **2b** at the solid/liquid interface of HOPG and a solution of **2b** in OA. (a) Overview STM image (61.1×61.1 nm^2, V_S = –0.8 V, I_t = 39 pA, c = 1×10^{-6} M, sample thermally annealed for 20 s at 100 °C prior to imaging); (b) supramolecular model of structural trapezoid motif with 11 MSWs; (c) supramolecular model of structural trapezoid motif with 12 MSWs; (d) supramolecular models of chiral structural rhomboid motifs with 10 MSWs each and the mirror plane between them.

3 Theoretical investigations of 2a and *n*-alkanes as a model system on graphene

3.1 General aspects

Geometry optimizations of supramolecular nanopatterns of **2a** including additional adsorbed octaonic acid (OA) dimers were investigated on graphene using GFN2-xTB methods.[S2-S4] The results will be discussed in chapter 3.2. Stimulated by these results, we aimed at a more general investigation regarding the orientation of aliphatic chains with respect to the underlying graphite surface. Therefore, we additionally investigated an *n*-alkanes (*n*-butane, *n*-hexane, *n*-octane) on graphene. The results will be discussed in chapter 3.3. Moreover, scanning tunneling microscopy images of backbones of MSWs were simulated. The results are shown in chapter 3.4. In all these cases, graphene cutouts of different sizes and shapes were utilized, representing a simplified model for the HOPG substrate used in our STM investigations.

3.2 Investigation of **2a** including solvent molecules on graphene

Supramolecular nanopatterns of **1a**, **1b**, **2a**, and **2b** were investigated by means of scanning tunneling microscopy (STM). The STM images contain bright and dark image regions, representing areas of low and high tunneling resistivities convoluted with high and low topographies. Based on these results, as a routine procedure, space-filling models are generated by using a graphical interface, here particularly using the commercial Wavefunction Spartan program package (cf. chapter 1.1). The results yield information about the exact packing of the molecules. In addition, much is known about the orientation of aliphatic chains which are commonly oriented along the main axis (*i.e.* zig-zag) directions of the HOPG substrate (see Main Text). However, in some cases, an STM image may contain imaging artefacts which may *e.g.* originate from tips of reduced symmetry, double tips, noise, etc. In addition, aliphatic chains oriented along the armchair directions of the substrate are highly unusual, and our observations provide a stimulus for more in-depth investigation.

The nanopattern experimentally observed by STM investigations of **2a** (cf. Figures 2c and r (Main Text) and Figures S11, S12, S14, and S15a) is one of these rare cases. Therefore, geometry optimizations of nanopatterns of **2a** were performed using the beta version of the xtb 6.3 program package, applying the force field method GFN-FF.[S3] The starting geometry includes four molecules of **2a** ($4 \times C_{393}H_{450}O_{18}$) and eight intercalated OA solvent molecules ($8 \times C_8H_{16}O_2$, observed as bright features in the STM image shown in Figure S15a, therein marked by arrows 4 to 7, as well as included in the supramolecular model shown in Figure S15b, therein marked by arrows 11 to 14) on the graphene cutout $C_{5644}H_{190}$. The geometry optimized structure is shown in Figure 3 (Main Text), and an enlarged reprint is shown in Figure S20.

As seen in Figure S20, the methoxy unit of one methyl benzoate groups (marked by arrow 1) of one molecule of **2a** is tilted out of the monolayer plane to avoid steric crowding with one of the OA chains. Moreover, another OA chain (marked by arrow 2) is pushed out of the monolayer plane and is mostly too far away from the graphene substrate to interact with it. For this solvent molecule, the hydrogen bond between its carboxylic acid group and an acid group of another OA molecule, embedded in the monolayer, is the only remaining interaction motif without which the OA molecule would desorb into the solution phase. Moreover, no other major changes were observed. The overall structural difference between the STM-based starting geometries and the optimized geometries, *i.e.* the heavy-atom-root-mean-square-deviation (hRMSD), is 1.36 Å.[S5] Therefore, the rather complex data treatment by means of GFN-FF validates the unusual packing observed by STM. It provides a deeper structural insight into the complexity of **2a** on HOPG. Four hexadecyloxy side chains per two MSWs are oriented along the (unfavorable) armchair direction of the HOPG substrate in a constrained system, and this packing is stabilized by intercalated solvent molecules. The insights generated from theory therefore provide a

deeper understanding on the exact geometries formed, and therefore go far beyond the pure experimental observation and classical STM data treatment.

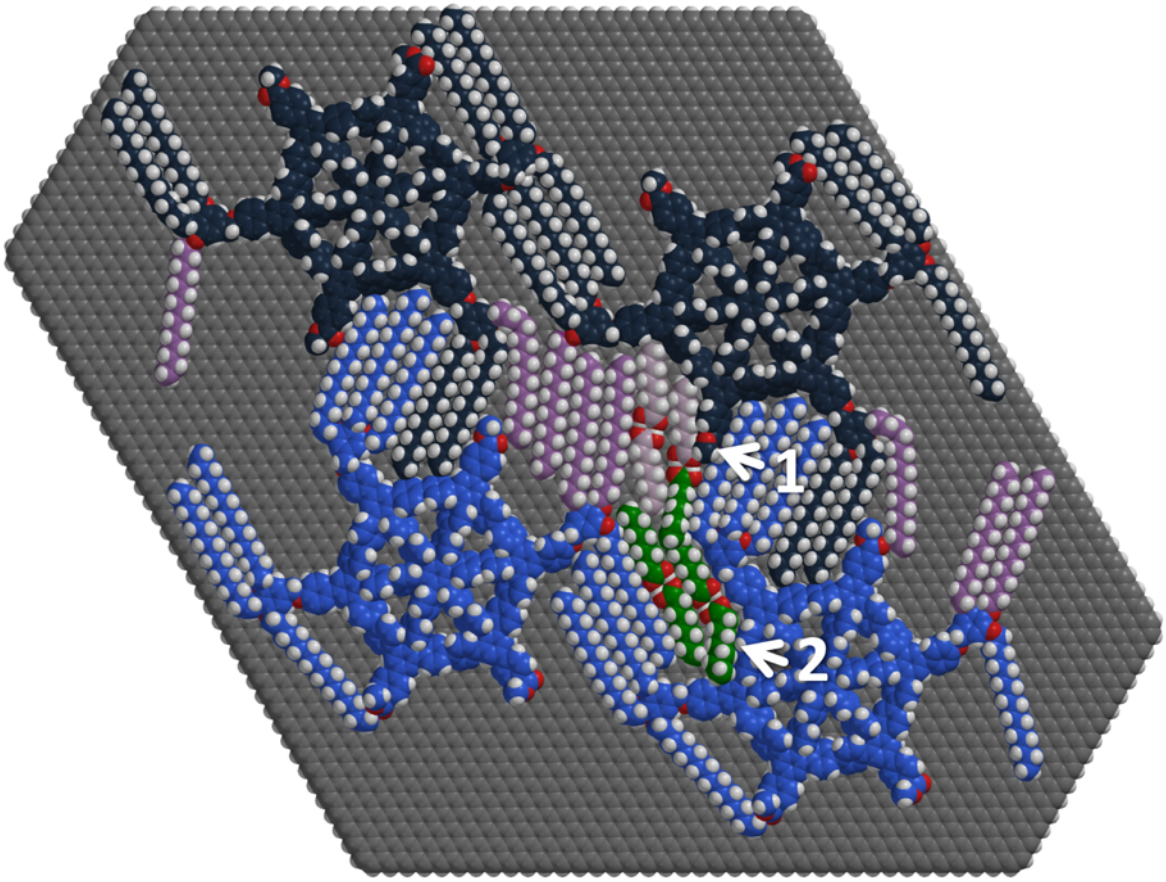

Figure S20: Enlarged reprint of Figure 3 (Main Text). Structure fragment (of 3652 atoms) of four MSWs **2a** (bright/dark blue and purple) with four OA dimers (pale purple and green), optimized at the GFN-FF level of theory on a hydrogen terminated graphene monolayer ($C_{5644}H_{190}$). Arrow 1 depicts a methyl benzoate group, tilted out of plane due to steric crowding. Similarly, arrow 2 indicates an OA molecule oriented out of plane, however still interacting with a surface-bound OA molecule *via* –COOH dimerization.

3.3 Theoretical experiments on *n*-alkane model systems to investigate energetic effects

3.3.1 General aspects

As described in chapter 3.2, the data obtained by the STM experiment (Figures 2c and r (Main Text) and Figures S11, S12, S14 and S15a) and related models obtained by GFN-FF based methods (Figures 3 (Main Text) and S20) imply that the unusual orientation of the four hexadecyloxy side chains per two molecules of **2a** along the HOPG armchair directions relies predominantly on steric reasons. To better understand the related energetic reasons, and to gain further insights into the armchair and zig-zag packaging motif, several model systems (differing in chain orientations) were generated and investigated by means of GFN2-xTB[S2] experiments. All these calculations were performed with the xtb 6.1[S4] program package, using normal convergence criteria. In total, four experiments will be presented here, addressing the following topics: 1) rotational barriers; 2) chain length dependence of barriers; 3) translational barriers; 4) influence of the graphene substrate.

3.3.2 Rotational barrier of n-hexane on graphene

The geometry of *n*-hexane was optimized on a hydrogen-saturated graphene cutout (with fixed coordinates of its C and H atoms) to yield a linear starting geometry of *n*-hexane as a model for *all-trans* alkanes. The resulting local minimum structure is aligned to be oriented in parallel to the graphene. Subsequently, the *n*-hexane chain was laterally shifted, so that its center of mass (*i.e.* the center of the C3–C4 bond) is localized in projection of a C–C bond of the graphene, and oriented in parallel to the latter. The so-obtained structure (blue in Figure S21) was rotated in -5° steps (clockwise) up to -180° (due to the C_2 symmetry of the *n*-hexane chain on graphene), and exemplary structures are shown in orange color in Figure S21. All structures were optimized with fixed dihedral angles, and energies (E_{rel}) are plotted vs. the rotation angle in Figure S21, therefore rotational barriers are obtained. The presumably global minimum at ≈ 65°, referenced to 0 kcal × mol^{-1}, shows the energetically favorable motif of *n*-hexane oriented along the HOPG main axis directions. Contrary, a local maximum is obtained when the *n*-hexane is rotated by 90°, corresponding to the armchair motif. However, barrier heights of 0.4 kcal × mol^{-1} are obtained, which are not sufficiently high to stabilize any specific rotamer at room temperature. However, in larger systems the chains are much longer, steric crowding is increased, and the alkyl/alkoxy chains are connected to a rigid (and more immobile) backbone, and all these factors prevents free chain rotation. The higher stability of the zig-zag motif is attributed to hydrogen atoms pointing into the hexagons of the graphene substrate. However, due to different environments of the carbon atoms in $–CH_2–$ and $–CH_3$ groups, we anticipate an influence of the chain lengths on the exact energies. Moreover, the results have limited relevance, as only the rotation of the *n*-hexane on graphene is discussed, but any possible translational degrees of freedom are neglected here. Therefore, a more detailed investigation including this aspect will be discussed in 3.3.4.

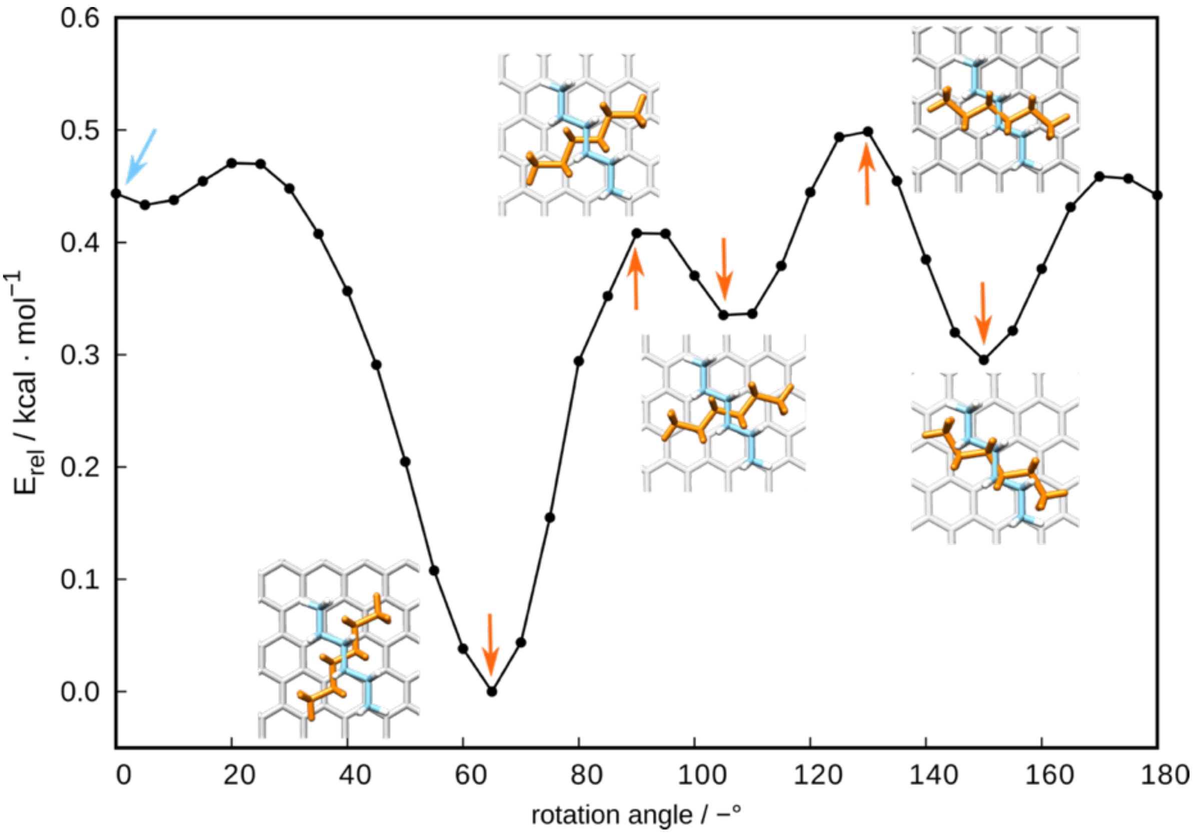

Figure S21: Relative energies of *n*-hexane on graphene *vs.* angle of rotation, obtained by constrained optimization on GFN2-xTB level with normal convergence criteria. Exemplary geometries are given. Relative energies are normalized to the minimum at –65°.

3.3.3 Chain length and number-of-chain dependence of rotational barriers

The dependence of the maximum rotational barrier, calculated between –150° and –60°, with the chain length was investigated. Therefore, constrained geometry optimizations at the respective angles of rotation (of the central alkane parts, cf. Figure S21) were performed. This exact procedure was performed for a single chain (blue in Figure S22) as well as three chains (yellow in Figure S22) lying next to each other. Three alkanes oriented in parallel inhibit each other more strongly from rotating due to repulsive interactions. Theoretically, the slope should be around trice as steep as for one chain. Although an increased barrier of 1.8 kcal × mol^{-1} is obtained for three chains here, and a stabilization is anticipated, the barrier height is still too low to allow for an observation of rotamers at room temperature. Therefore, these results cannot explain the orientation of hexadecyloxy side chains of **2a** along the armchair directions of the HOPG substrate, as observed by STM (Figures 2c and r (Main Text) and Figures S11, S12, S14 and S15a).

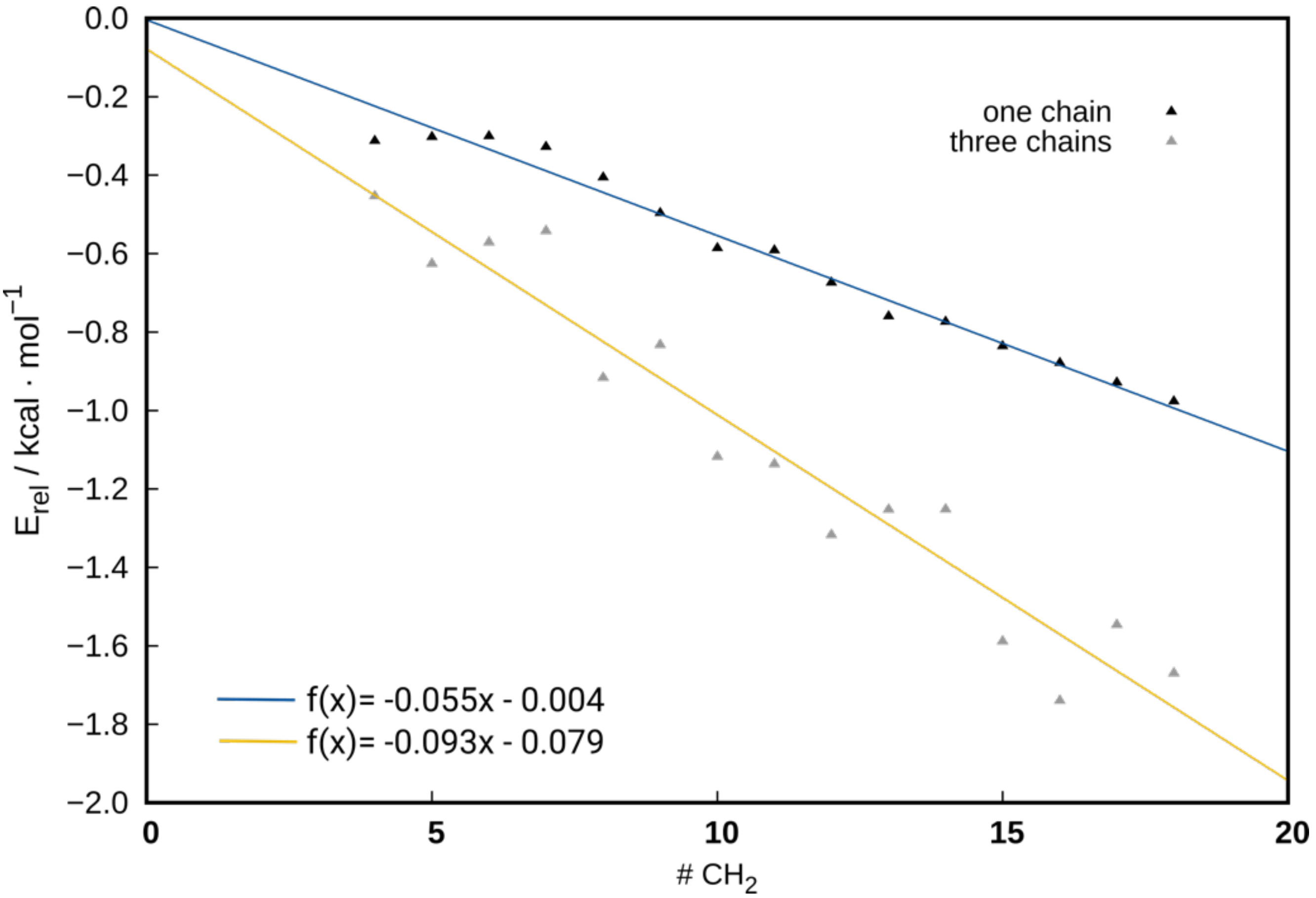

Figure S22: Barrier of rotation heights of single alkanes (blue) and triples of alkanes (yellow) of different chain lengths (#CH_2), calculated in the range of –150° and –60° on GFN2-xTB optimized level. Linear fits should ideally end at (0|0).

3.3.4 Scanning the graphene grid to evaluate translational barriers

For an *n*-alkane on graphene, C_6 symmetry of the substrate will lead to a periodicity regarding rotation. Furthermore, translational degrees of freedom should be considered in addition to the previously discussed rotation of the alkanes (cf. chapter 3.3.2). Therefore, the potential energy surfaces (PESs) of the translations of three alkanes of different lengths (*i.e. n*-butane, *n*-hexane, *n*-octane) with different angles of rotation (as defined in chapter 3.3.2) are calculated. More precisely, 72.000 single point calculations with GFN2-xTB were conducted for each of the three systems. The respective alkane is moved in 0.05 Bohr steps in an area of 10×18 Bohr^2, which corresponds to the size of ≈ eight hexagons of the graphene substrate. We illustrate this procedure in smaller dimensions in Figure S23.

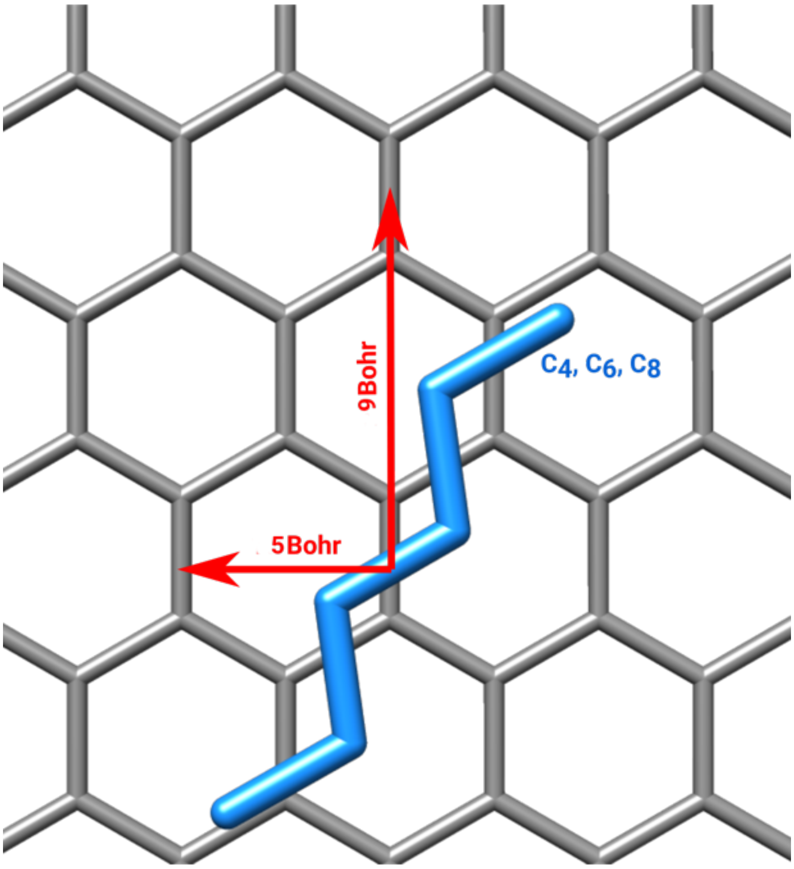

Figure S23: Experimental settings how to scan potential energy surfaces (PES). The red arrows show translation vectors of identical adsorbate geometries.

Exemplary results of the aforementioned procedure are shown for the structures obtained at an angle of rotation of –65° (Figures S24 and S25) and –90° (Figure S26). All graphs are truncated at 10 Bohr in the *y*-direction due to finite size of the graphene substrate. The relative energies are normalized to the minimum. Differences of the potential minima are related to an intrinsic curvature of the alkane backbone. Barriers of translation (of 0.6 kcal × mol^{-1}, cf. Figure S21) and rotation (0.4 kcal × mol^{-1}) are of similar height, as expected. (Note that unrelaxed scans but not optimized structures are considered). The minima are localized along the three zig-zag directions, as shown in Figure S25. C_6 symmetry of the substrate is reflected in scans at different angles (data not shown), and certain angles of rotation and vectors of translation allow the hydrogen atoms to point into the hexagons of the graphene substrate, which is anticipated to be an ideal adsorption geometry.

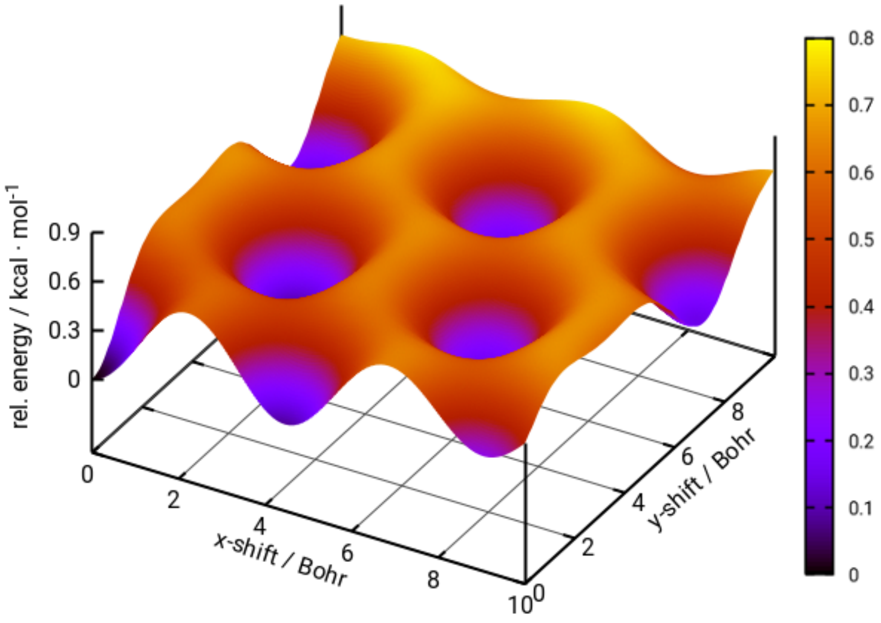

Figure S24: GFN2-xTB single point PES of relative energies in kcal mol^{-1} depending on the position of hexane, rotated by -65°, on a graphene substrate.

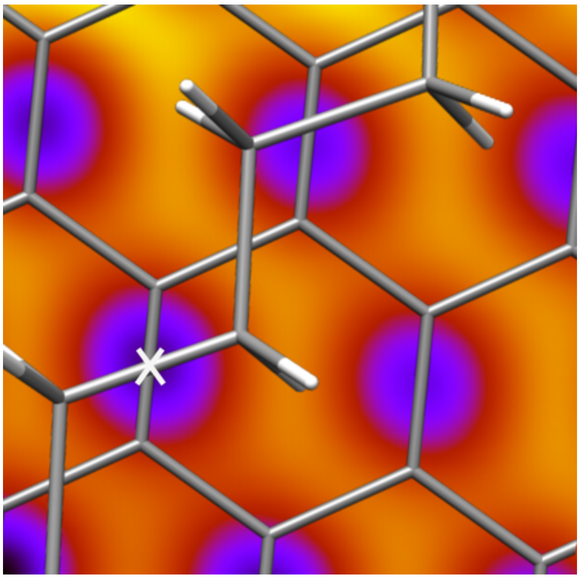

Figure S25: 2D representation of GFN2-xTB single point PES of Figure S22 together with graphene and hexane. The center of mass, which marks the shift-position, is highlighted with a cross.

Other angles of rotation (*e.g.* –90°) are not able to adopt the zig-zag motif and thus show a completely different PES, as shown in Figure S26. In contrast to other PES showing a spot-like distribution of minima, *e.g.* the PES obtained at –65° (cf. Figure S24 and S25), energetically more favorable structures are distributed in a channel-like fashion (cf. Figure S26). In addition, along the unfavorable channels, encircled regions (in turn) highlight energetically most favorable structures therein.

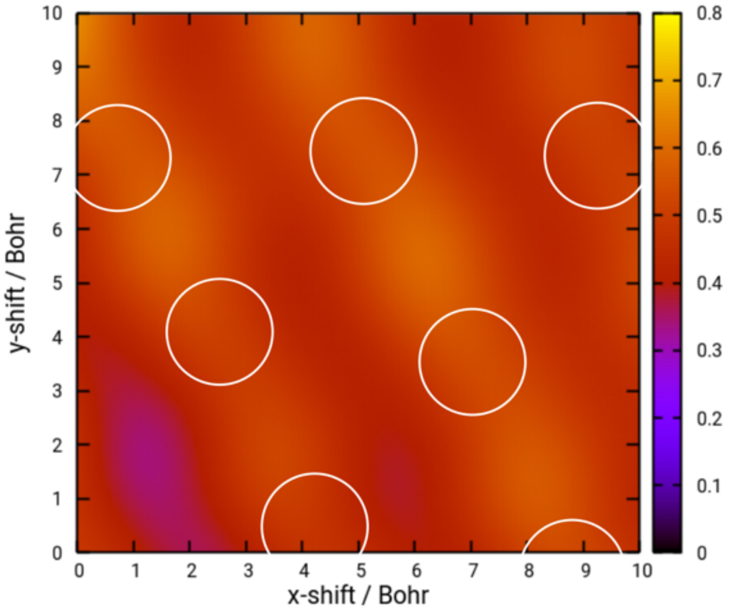

Figure S26: 2D GFN2-xTB single point PES of hexane rotated by –90° on a graphene substrate; relative energy in kcal × mol^{-1} normalized to the global minimum (of the structure obtained at –65°). White circles indicate local minima within the energetically unfavorable channels.

Some structures within the encircled regions (Figure S26) and different angles of rotation are shown in Figure S27, visualizing the expected translational equivalence of every 60°.

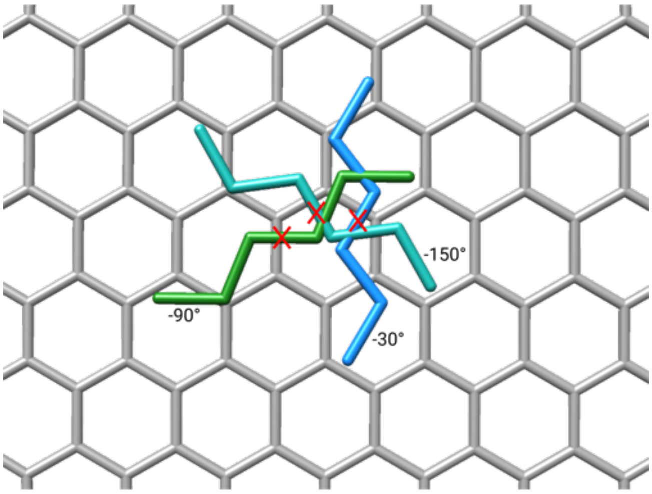

Figure S27: Representative rotamers in which the alkanes are oriented along different armchair directions of the graphene substrate, corresponding to encircled areas of Figure S26. Hydrogen atoms are omitted for clarity. Red crosses indicated the centers of mass of the alkanes.

Conclusively, considering rotation as well as translation, the armchair motif is neither a global energetic maximum nor an energetically favored structure on the PES, but is only ≈ 0.5 kcal × mol^{-1} less stable as compared to the zig-zag motif, which is irrelevant at room temperature.

Finally, the scans were conducted with different angles of rotation (as shown in Figure S21) for *n*-butane, *n*-hexane and *n*-octane. For each angle of rotation, the energetic minimum was obtained by translation (as shown in Figure S23). The resulting energies *vs.* angles of rotation are plotted in Figure S28.

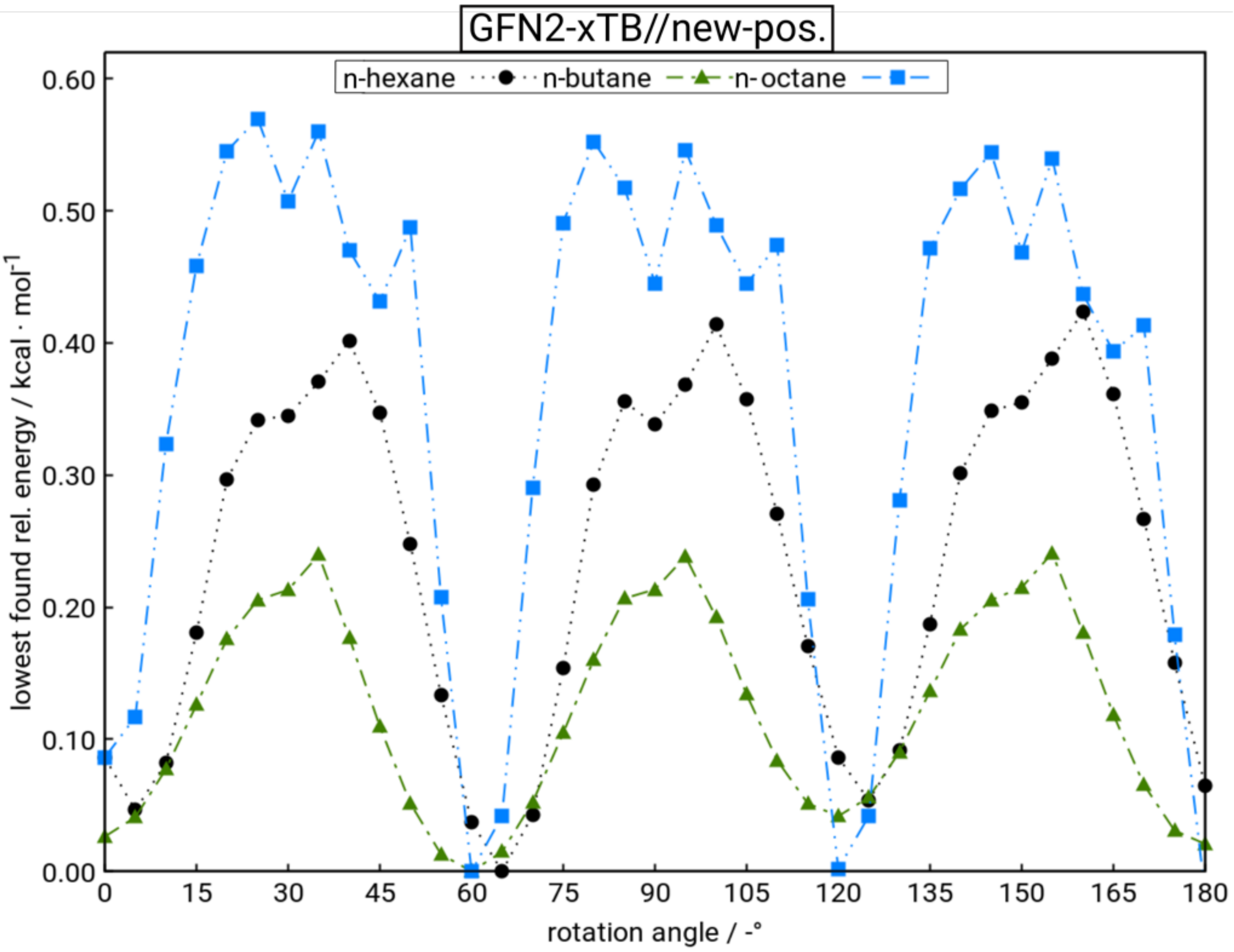

Figure S28: Lowest found relative energies at GFN2-xTB single point level, normalized to the global energetic minimum in dependence of the angle of rotation of three different alkanes (*n*-butane, *n*-hexane, *n*-octane). Lines are added to guide the eye, and energies are not necessarily recorded at the same positions.

Figure S29 shows which conformers (and positions, omitted here) will be adopted by *n*-butane (Figure S29a), *n*-hexane (Figure S29b), and *n*-octane (Figure S29c) as energetic minimum structures after rotation and translation on a graphene sheet, like in the case of diluted deposition from the gas-phase or a solvent. As previously described, the barriers increase with increasing chain length. Remarkable but not astonishing is, that also the number of local minima increases with chain length, which is explained by statistics.

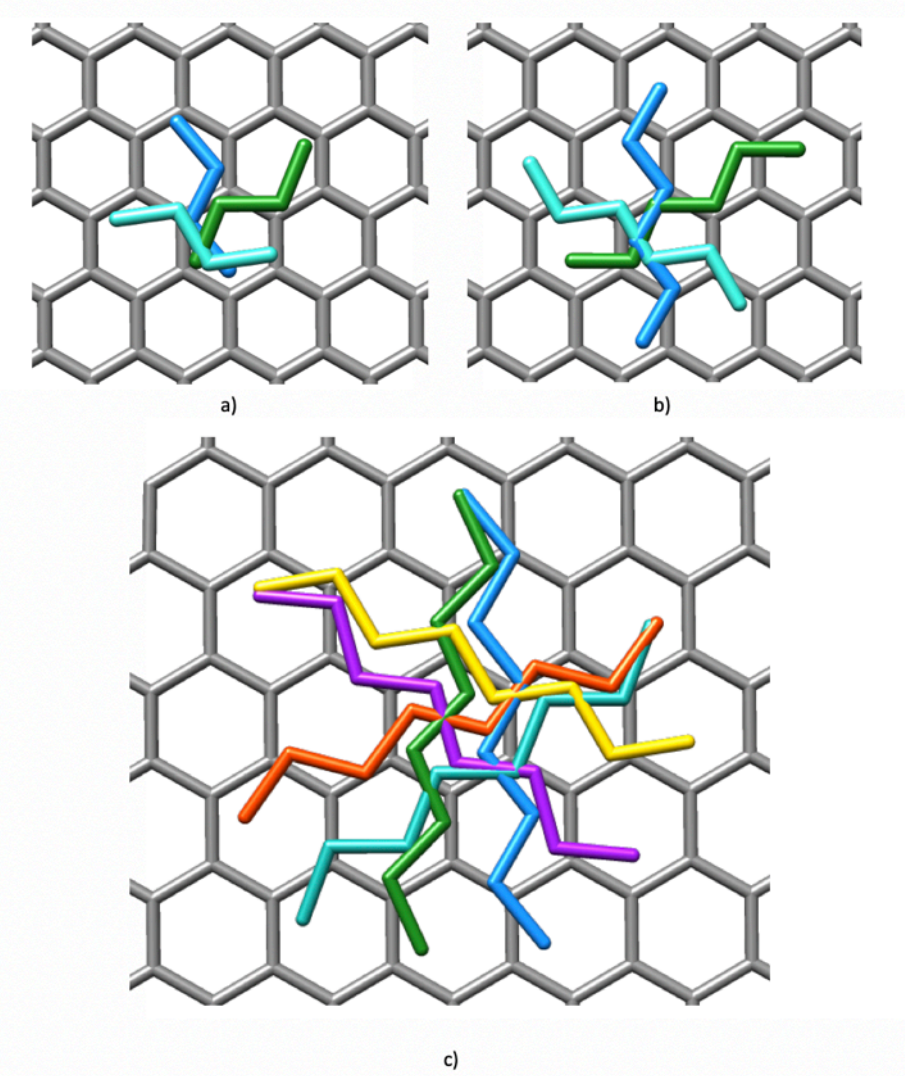

Figure S29: GFN2-xTB single point minimum structures of a) *n*-butane at –30°, –90°, –150° b) *n*-hexane at –30°, –90°, –150° c) *n*-octane at –30°, –45°, –90°, –105°, –150°, –165° taken from Figure S28. Hydrogen atoms are omitted for clarity.

All displayed structures show the armchair motif or a structure close to it, as expected for local minima. The position of the terminal $–CH_3$ groups is relevant. Contributions of the structures in which terminal $–CH_3$ groups adopt energetically unfavorable positions (*i.e.* located close to a C–C bond of the substrate) are reduced with increasing chain length.

Conclusively, the observed packing motif (cf. Figures 2c and r (Main Text) and Figures S12, S14, and S15a) in which four hexadecyloxy side chains per two molecules of **2a** on HOPG are oriented along the armchair directions cannot be solely explained by local energetic minima of single *n*-alkanes on graphene. Therefore, we conclude that confinement effects are crucial. This is consistent with the experimental observations of *n*-alkanes and cycloalkanes on HOPG (see Main Text).

3.4 Computed STM images of MSWs

As STM images are highly dependent on the geometry, the experimental unoptimized cutout was used to stay as close to the reference as possible. The following structure shown in Figure S30 has been investigated.

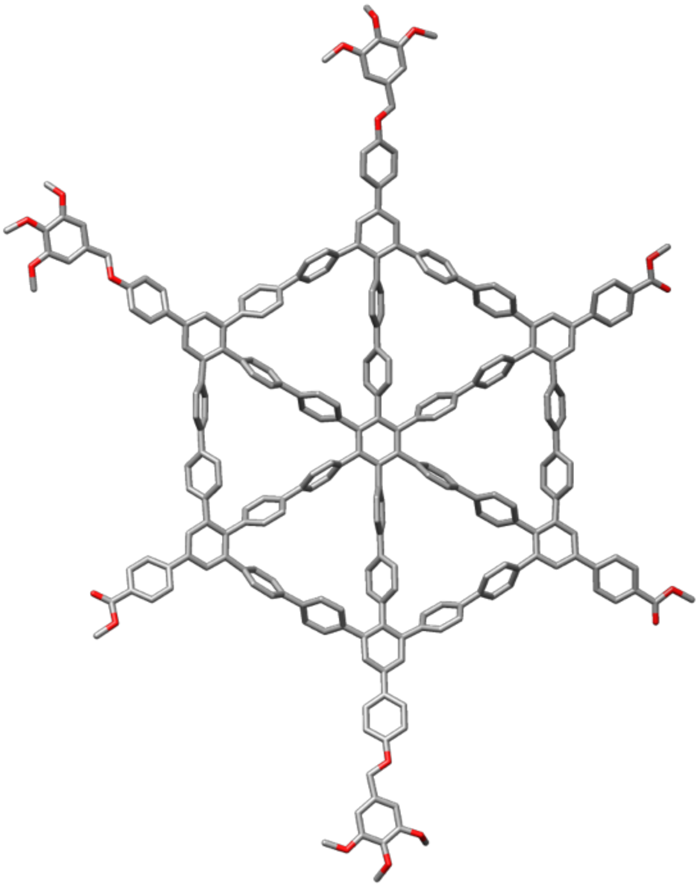

Figure S30: Cutout from experimental structure of MSW **2a**.

A fraction of the STM image, corresponding the MSW backbone, was calculated with GFN2-xTB on a graphene monolayer. The system size was around 3200 atoms. The result is depicted in Figure S31.

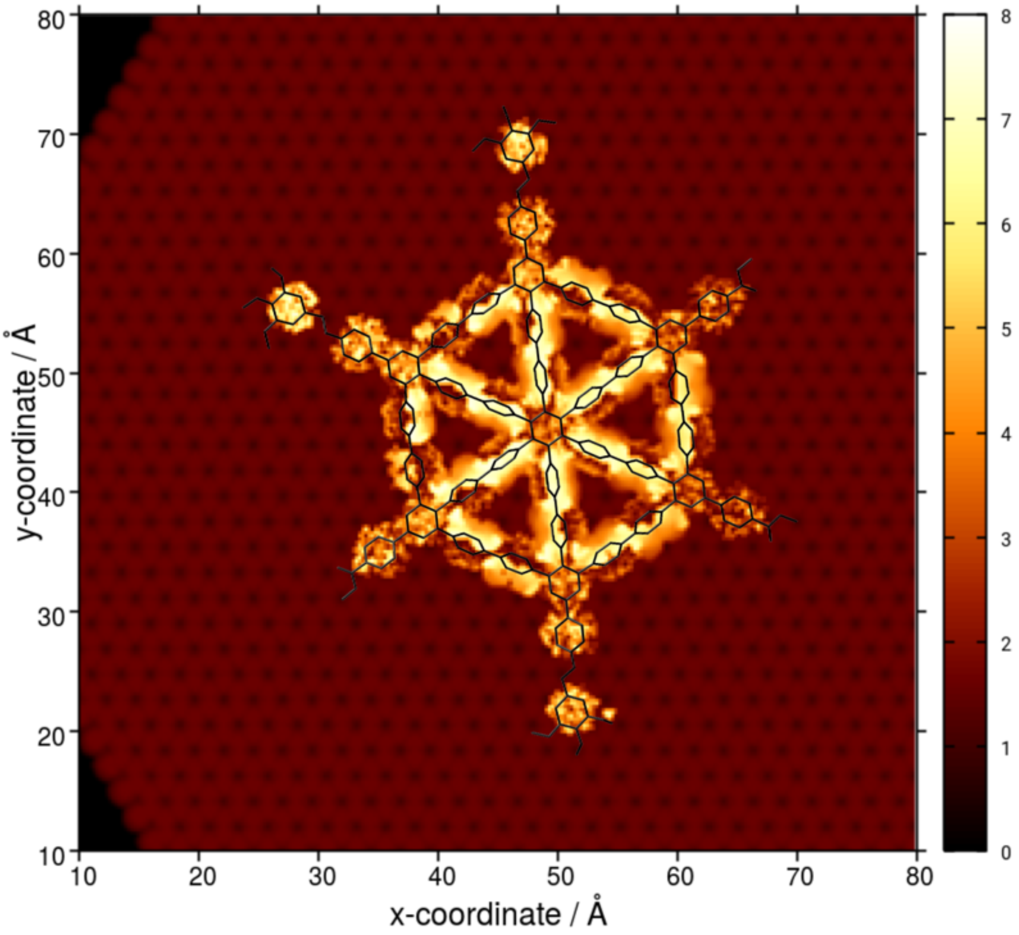

Figure S31: Simulated STM image of an experimental unoptimized MSW cutout with a black overlay of the corresponding structure. High electron densities are represented by lighter colors.

All aromatic rings are clearly visible, even the rotational position compared to the graphene is reproduced. This image is compared to an experimental one in Figure S32. In general, it should be highlighted that STM images for such large systems can be calculated within a week on a normal tabletop computer with good agreement to the experiment.

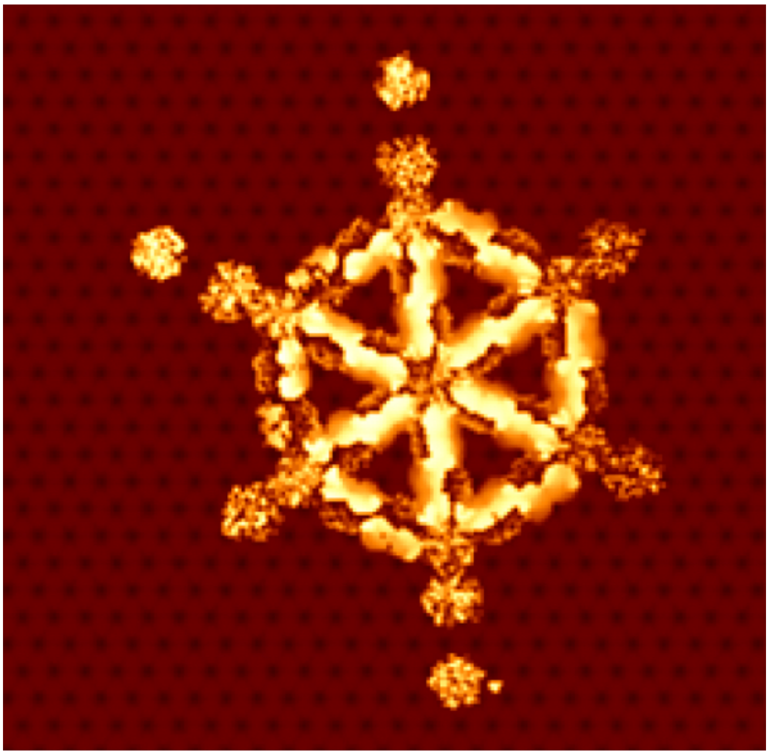

a)

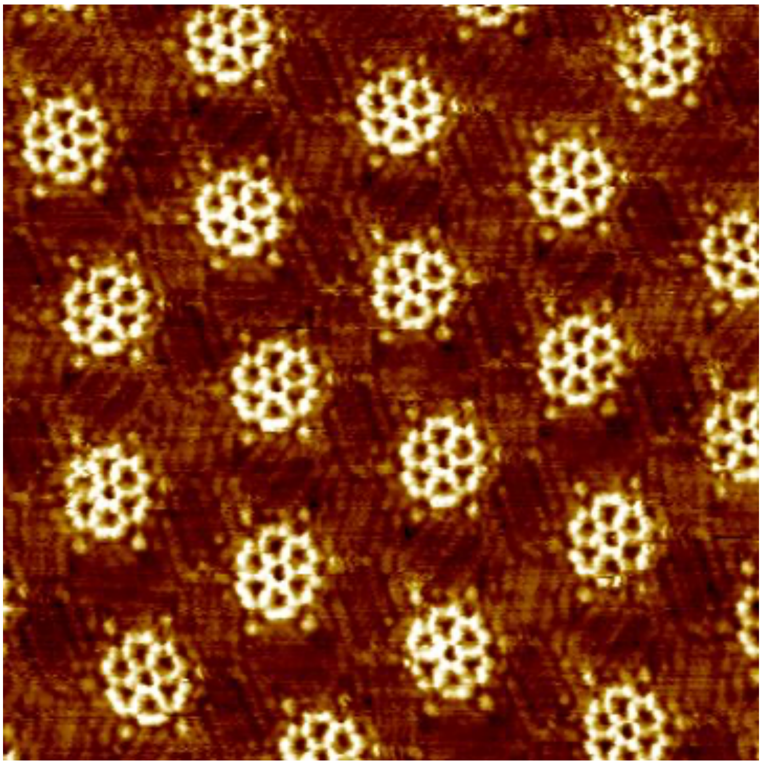

b)

Figure S32: Comparison of a) computed and b) experimental STM images of MSWs calculated on GFN2-xTB level.

4 Synthesis and characterization

4.1 Depiction of the synthesis

The synthesis of MSWs **1a**/**b** and **2a**/**b** in isomerically pure form was performed as shown in Scheme S1. It starts with the Lewis acid-catalyzed condensation of **3** and **4** to form the pyrylium salt **5**. The Zimmermann-Fischer condensation of **5** with **6** leads to **7**,[S6] which is coupled with **8** (obtained in two steps from **7**) to the asymmetric acetylene **9**. **8** provides the required solubility of the subsequent compounds through the trialkoxybenzyl groups.[S7] The wheel precursors **10a** (17 %) and **10b** (38 %) are obtained as a mixture of regioisomers by Vollhardt acetylene trimerization of **9** using $Co_2(CO)_8$ as the catalyst.

The arrangement of the three ester groups in **10b** leads to a net dipole moment of the compound, which makes it more polar than **10a**, in which the different polarities of the alkoxy- and carboxymethyl-groups are canceled out. Therefore, both compounds have a different R_f value on TLC (normal phase), and a chromatographic separation of the isomers by column chromatography on silica gel is possible (cyclohexane/dichloromethane (Cy/DCM): 1/2, R_f(**10a**) = 0.76, R_f(**10b**) = 0.68). The final ring closure by a nickel mediated Yamamoto coupling of **10a** and **10b**, respectively, yields the MSWs **1a** (88 %) and **2a** (90 %) as slightly beige, nearly white solids in high yields.

Scheme S1: [a]Reagents and conditions: a) $BF_3 \times OEt_2$, 1,2-dichlorethane, 5 h, reflux, 22 %; b) benzoic anhydride, 150 °C, 4 h, 40 %; c) $PdCl_2(PPh_3)_2$, **6**, CuI, PPh_3, THF, piperidine, r.t., 18 h; 65 %; d) $Co_2(CO)_8$, toluene, reflux; 55 % (**10a**, 17 %, **10b**, 38 %); e/g) $Ni(COD)_2$, bipyridine, THF, COD, microwave (120 °C, 12 min); **1a**: 88 %; **2a**: 90 %; f/h) LiOH, THF, H_2O, 18 h, **1b**: 88 %; **2b**: 87 %.

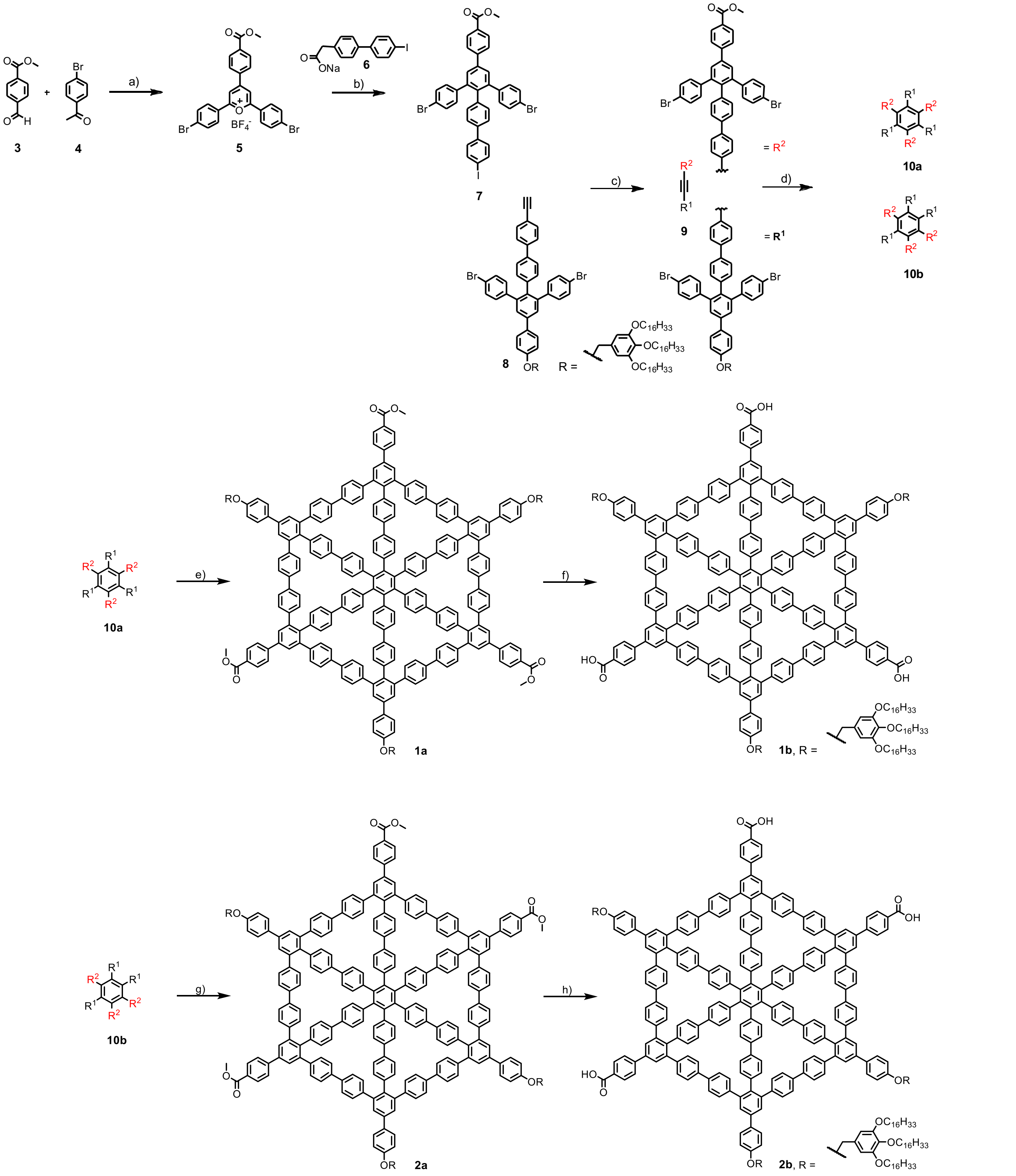

4.2 Synthesis of the compounds

5

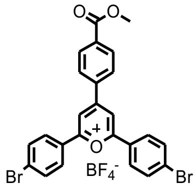

4′-Bromoacetophenone **4** (27.2 g, 136 mmol), methyl 4-formylbenzoate **3** (11.2 g, 68.2 mmol) and boron trifluoride diethyl etherate (25.0 mL, 197 mmol) were placed in dry Schlenk tube, that was purged with Ar. The mixture was stirred in 1,2-dichlorethane (50 mL) for 5 h at 80 °C. The reaction mixture was diluted with DCM (5 mL) and added to ethyl acetate (200 mL). The precipitate was filtered off and washed with diethyl ether. Removal the solvent led to **5** (9.00 g, 14.7 mmol, 22 %) as a yellow solid.

Chemical formula: $C_{25}H_{17}BBr_2F_4O_3$. **^{1}H NMR** (400 MHz, DMSO-d$_6$, r.t.): δ [ppm] = 9.27 (s, 2H), 8.69 (d, $^3J_{HH}$ = 8.2 Hz, 2H), 8.54 (d, $^3J_{HH}$ = 8.8 Hz, 4H), 8.28 (d, $^3J_{HH}$ = 8.5 Hz, 2H), 8.04 (d, $^3J_{HH}$ = 8.7 Hz, 4H), 3.96 (s, 3H). **^{13}C NMR** (176 MHz, DMSO-d$_6$, r.t.): δ [ppm] = 169.82, 169.70, 165.79, 131.80, 130.73, 130.14, 129.33, 128.19, 127.48, 127.38, 123.99, 116.48, 52.77. **MS** (ESI+), *m/z* (%): 524.95 (100) [M]$^+$.

7

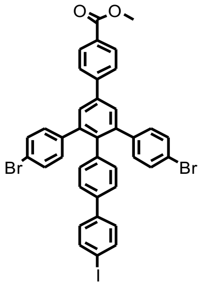

6 (3.00 g, 8.33 mmol), **5** (5.61 g, 9.17 mmol) and benzoic anhydride (28.3 g, 125 mmol) were purged with Ar three times and stirred for 15 min at 70 °C. Then the solution was stirred for 4 h at 150 °C under Ar. After the mixture was cooled at r.t., the residue was dissolved in DCM and Cy (1:1) and purified by column chromatography (Cy:DCM = 1:1). The brown solid was recrystallized from DCM and filtered. The residue was washed with cold DCM, and the solvent was removed *in vacuo*. **7** (2.84 g, 3.55 mmol, 43 %) was obtained as a white solid.

Chemical formula: $C_{38}H_{25}Br_2IO_2$. **^{1}H NMR** (700 MHz, CDCl$_3$, r.t.): δ [ppm] = 8.18 – 8.14 (m, 2H), 7.79 – 7.73 (m, 4H), 7.69 (s, 2H), 7.35 (d, $^3J_{HH}$ = 8.5 Hz, 4H), 7.32 – 7.29 (m, 4H), 7.04 (d, $^3J_{HH}$ = 8.5 Hz, 4H), 6.92 (s, 2H), 3.98 (s, 3H). **^{13}C NMR** (176 MHz, CDCl$_3$, r.t.): δ [ppm] = 167.01, 144.53, 141.80, 140.45, 139.92, 137.95, 132.13, 131.59, 131.18, 130.41, 129.56, 128.85, 128.73, 128.25, 127.20, 126.13, 121.18, 93.19, 52.36. **MS** (MALDI-pos, DCTB), *m/z* (%): 798.0 (100) [M]$^+$.

9

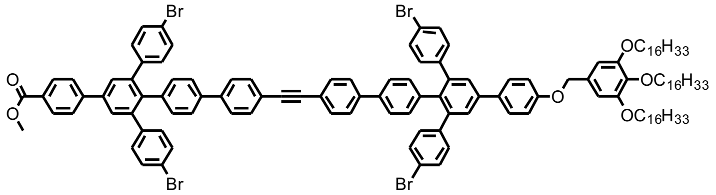

In a dry Schlenk tube **8** (1.28 g, 0.870 mmol) and **7** (0.700 g, 0.870 mmol) were dissolved in THF (10 mL) and triethylamine (20 mL) and purged with Ar for 1 h. $PdCl_2(PPh_3)_2$ (12.3 mg, 17.5 µmol), PPh_3 (13.8 mg, 52.5 µmol) and CuI (4.99 mg, 26.2 µmol) were added. The solution was stirred for 18 h at r.t. The reaction mixture was diluted with HCl (10 %) and DCM. After extraction of the aqueous layer with DCM, the combined organic phases were washed with water and brine and dried over $MgSO_4$. Removal of the solvent and purification by column chromatography (Cy:DCM = 1:1, R_f = 0.5) yielded pure **9** (1.20 g, 0.56 mmol, 65 %) as a colorless solid.

Chemical formula: $C_{131}H_{150}Br_4O_6$. **^{1}H NMR** (400 MHz, CD_2Cl_2, r.t.): δ [ppm] = 8.13 (d, $^3J_{HH}$ = 8.4 Hz, 2H), 7.80 (d, $^3J_{HH}$ = 8.4 Hz, 2H), 7.71 (s, 2H), 7.67 – 7.62 (m, 4H), 7.58 (s, 8H), 7.40 – 7.31 (m, 12H), 7.10 – 7.04 (m, 10H), 7.00 – 6.94 (m, 4H), 6.65 (s, 2H), 5.01 (s, 2H), 4.01 – 3.88 (m, 9H), 1.85 – 1.76 (m, 4 H), 1.74 – 1.67 (m, 2H), 1.50 – 1.42 (m, 6H), 1.38 – 1.23 (m, 72H), 0.88 (t, $^3J_{HH}$ = 6.8 Hz, 9H). **^{13}C NMR** (126 MHz, CD_2Cl_2, r.t.): δ [ppm] = 167.20, 159.40, 153.86, 144.93, 142.24, 141.98, 141.44, 141.12, 140.67, 140.59, 140.52, 139.79, 138.89, 138.73, 138.52, 138.32, 137.41, 133.20, 132.75, 132.64, 132.49, 132.21, 132.18, 131.47, 131.39, 130.68, 130.10, 129.08, 128.67, 128.47, 127.61, 127.27, 127.25, 126.51, 126.45, 122.73, 122.66, 121.39, 121.22, 115.86, 106.47, 90.54, 73.91, 71.02, 69.62, 52.61, 32.52, 32.52, 30.94, 30.36, 30.34, 30.32, 30.30, 30.27, 30.25, 30.24, 30.21, 30.02, 29.96, 29.95, 26.74, 26.71, 23.28, 14.46. **MS** (MALDI-pos, DCTB), *m/z* (%): 2947.8 (90) $[M+C_{55}H_{103}O_3]^+$, 2134.7 (40) $[M]^+$, 1324.2 (100) $[M-C_{55}H_{102}O_3]^+$.

10a and 10b

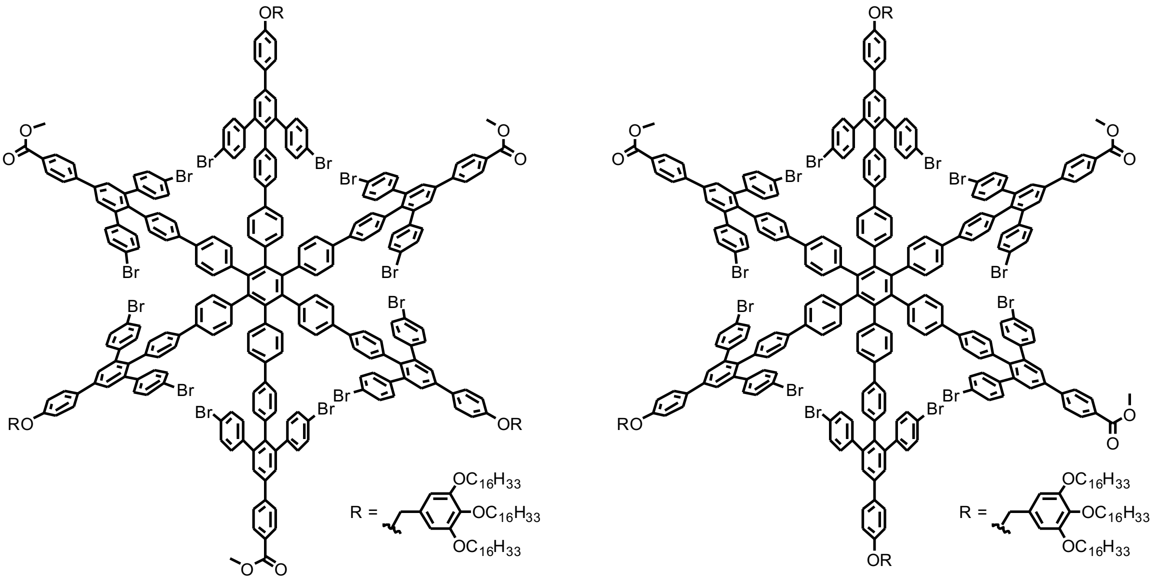

In a dry Schlenk tube **9** (200 mg, 93.5 µmol) was dissolved in toluene (10 mL) and purged with Ar for 1 h. Then $Co_2(CO)_8$ (8.1 mg, 23.4 µmol) was added and the reaction was stirred for 4 h at 125 °C. The reaction was diluted with water and DCM. After extraction of the aqueous layer with DCM, the combined organic phases were washed with water and brine and dried over $MgSO_4$. Removal of the solvent and purification by recycling GPC and column chromatography (Cy/DCM = 1:2, R_f = 0.76) yielded pure **10a** (33.6 mg, 5.23 µmol, 17 %) and **10b** (74.9 mg, 11.7 µmol, 38 %) as colorless solids.

Chemical formula: $C_{393}H_{450}Br_{12}O_{18}$. **^{1}H NMR** (500 MHz, , CD_2Cl_2, r.t.): δ [ppm] = 8.11 (d, $^3J_{HH}$ = 8.0 Hz, 6H), 7.78 (d, $^3J_{HH}$ = 8.1 Hz, 6H), 7.68 (s, 6H), 7.63 (d, $^3J_{HH}$ = 8.3 Hz, 6H), 7.59 (s, 6H), 7.29 (t, $^3J_{HH}$ = 6.3 Hz, 24H), 7.23 – 7.10 (m, 24H), 7.10 – 6.98 (m, 36H), 6.95 (s, 6H), 6.90 – 6.81 (m, 12H), 6.64 (s, 6H), 4.99 (s, 6H), 3.97 (t, $^3J_{HH}$ = 6.5 Hz, 12H), 3.94 – 3.88 (m, 15H), 1.87 – 1.64 (m, 18H), 1.52 – 1.39 (m, 18H), 1.40 – 1.19 (m, 216H), 0.93 – 0.75 (m, 27H). **^{13}C NMR** (125 MHz, CD_2Cl_2, r.t.): δ [ppm] = 166.61, 158.79, 153.26, 144.35, 144.34, 141.65, 141.64, 141.63, 141.62, 141.61, 141.40, 141.39, 141.38, 141.37, 140.92, 140.62, 140.60, 140.56, 140.19, 139.81, 139.79, 139.76, 139.05, 138.36, 137.72, 136.87, 136.86, 132.63, 132.00, 131.62, 131.59, 130.90, 130.81, 130.07, 129.45, 128.54, 128.07, 127.92, 126.99, 125.73, 124.94, 120.80, 120.61, 115.22, 105.89, 73.32, 70.44, 69.02, 31.93, 30.35, 29.77, 29.76, 29.76, 29.74, 29.71, 29.68, 29.66, 29.65, 29.62, 29.44, 29.43, 29.38, 29.36, 26.15, 26.12. **MS** (MALDI-pos, DCTB), *m/z* (%):6464.6 (100) $[M+K]^+$. GPC (PS calibration): M_P = 8451 g mol^{-1}.

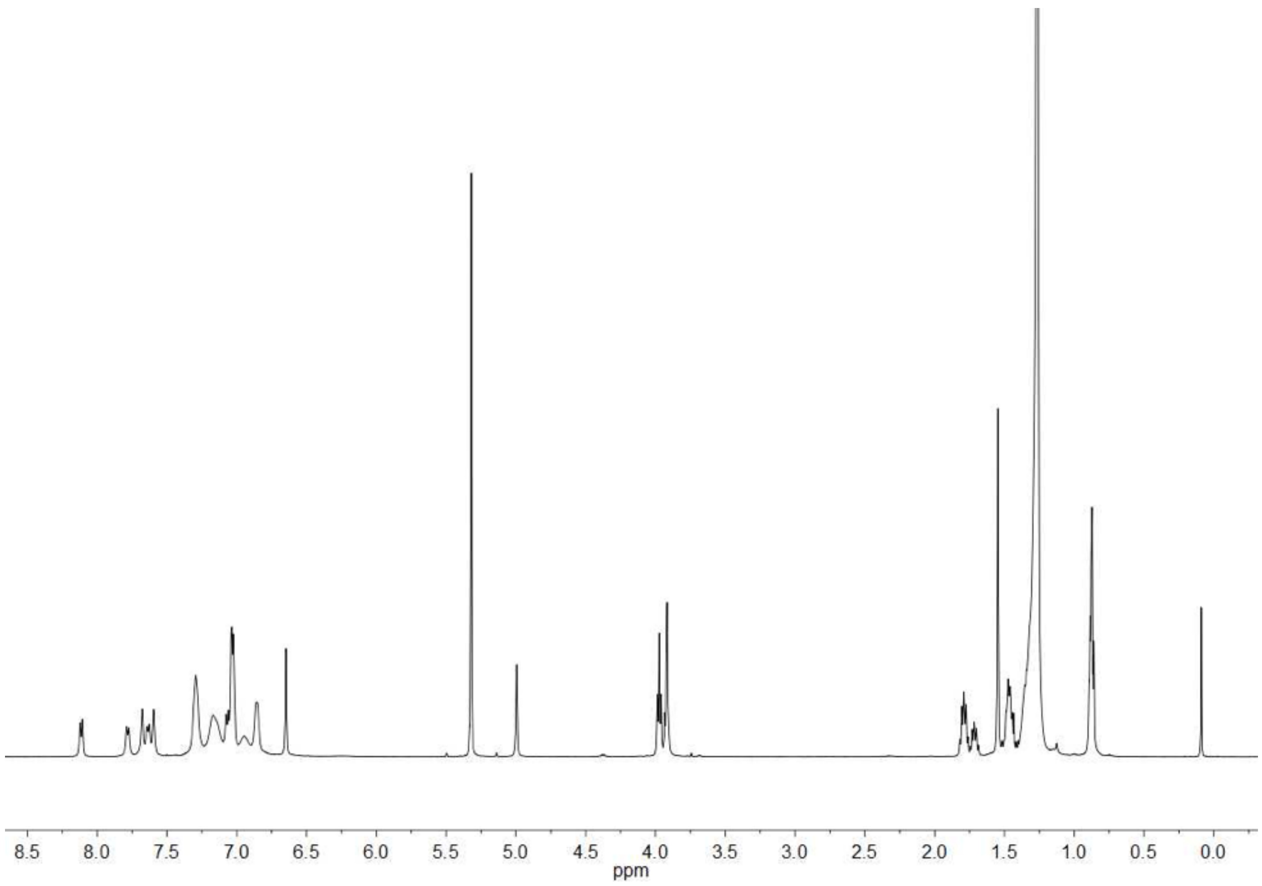

Figure S33: ^{1}H NMR spectrum of **10a** in CD_2Cl_2.

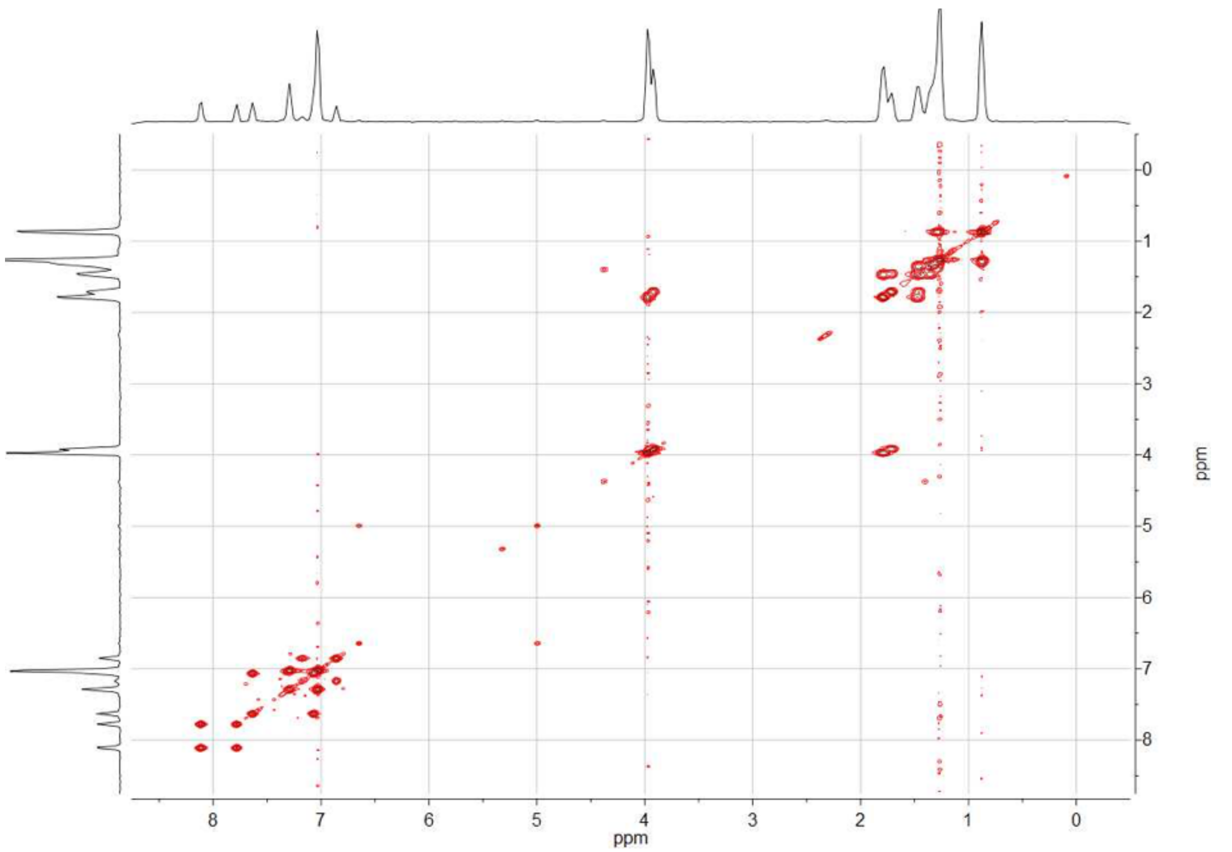

Figure S34: ^{1}H-^{1}H COSY NMR spectrum of **10a** in CD_2Cl_2.

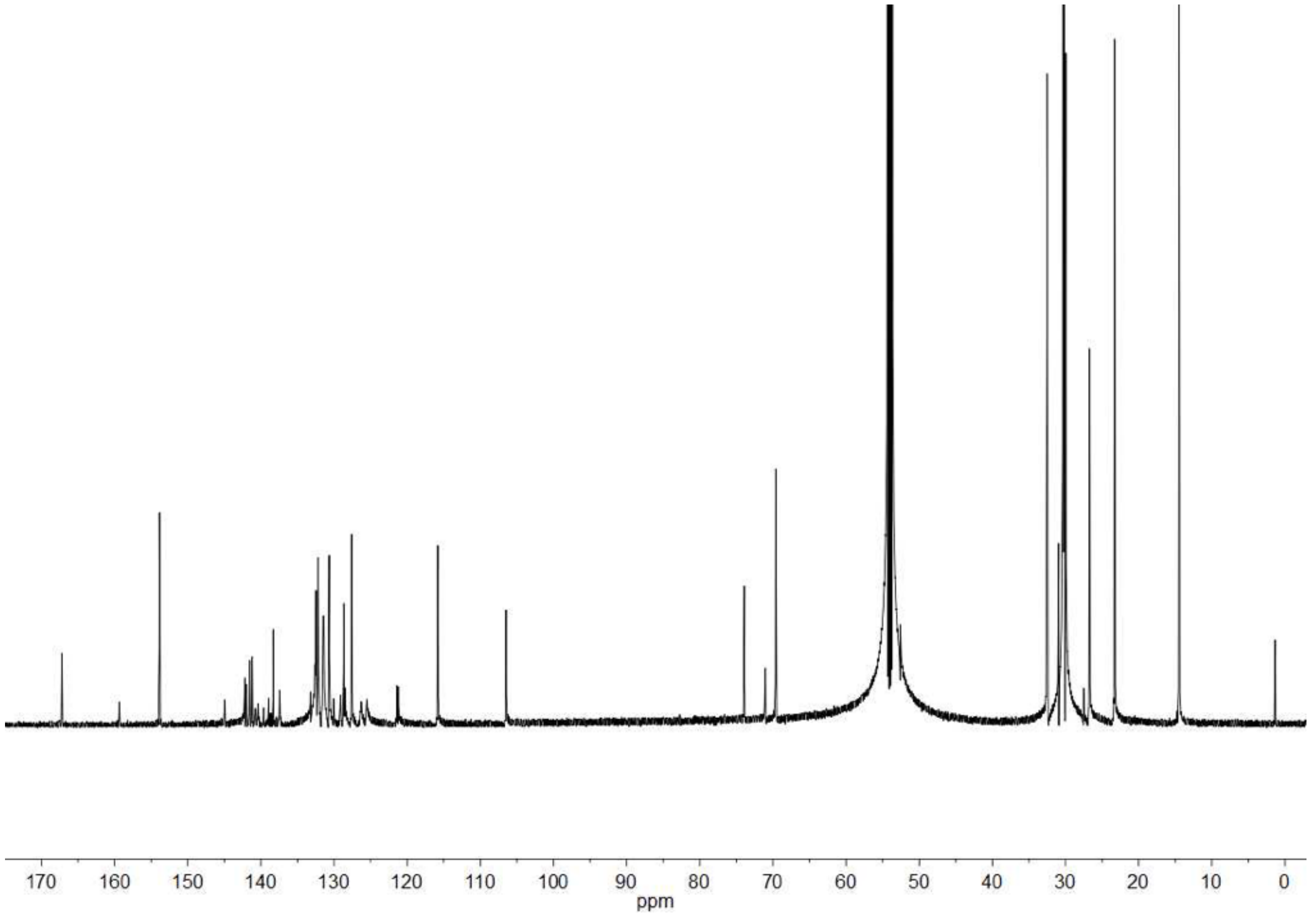

Figure S35: ^{13}C NMR spectrum of **10a** in CD_2Cl_2.

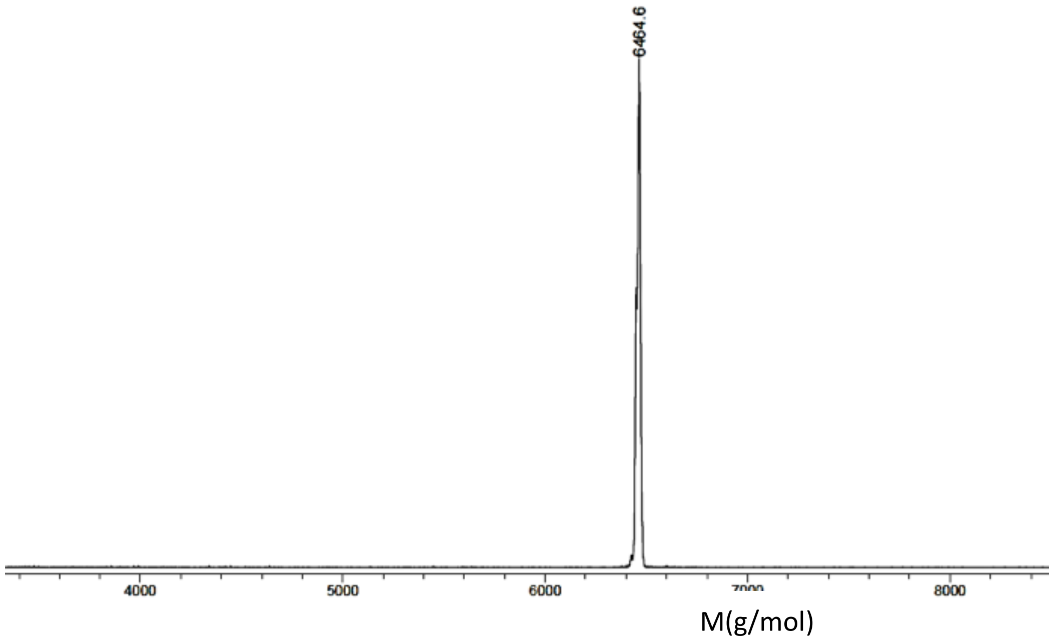

Figure S36: MALDI-TOF mass spectrum of **10a** (matrix: DCTB).

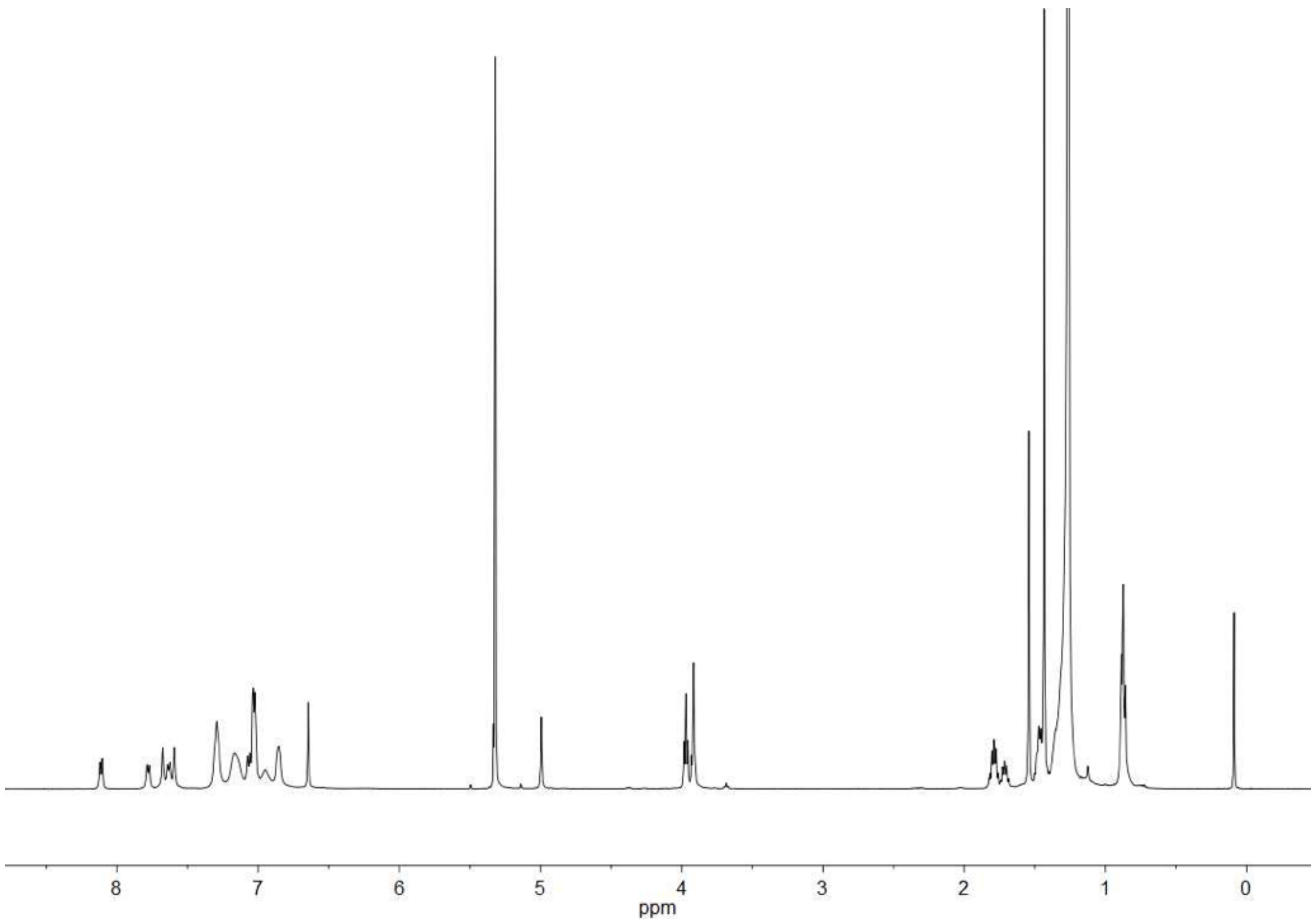

Figure S37: ^{1}H NMR spectrum of **10b** in CD_2Cl_2.

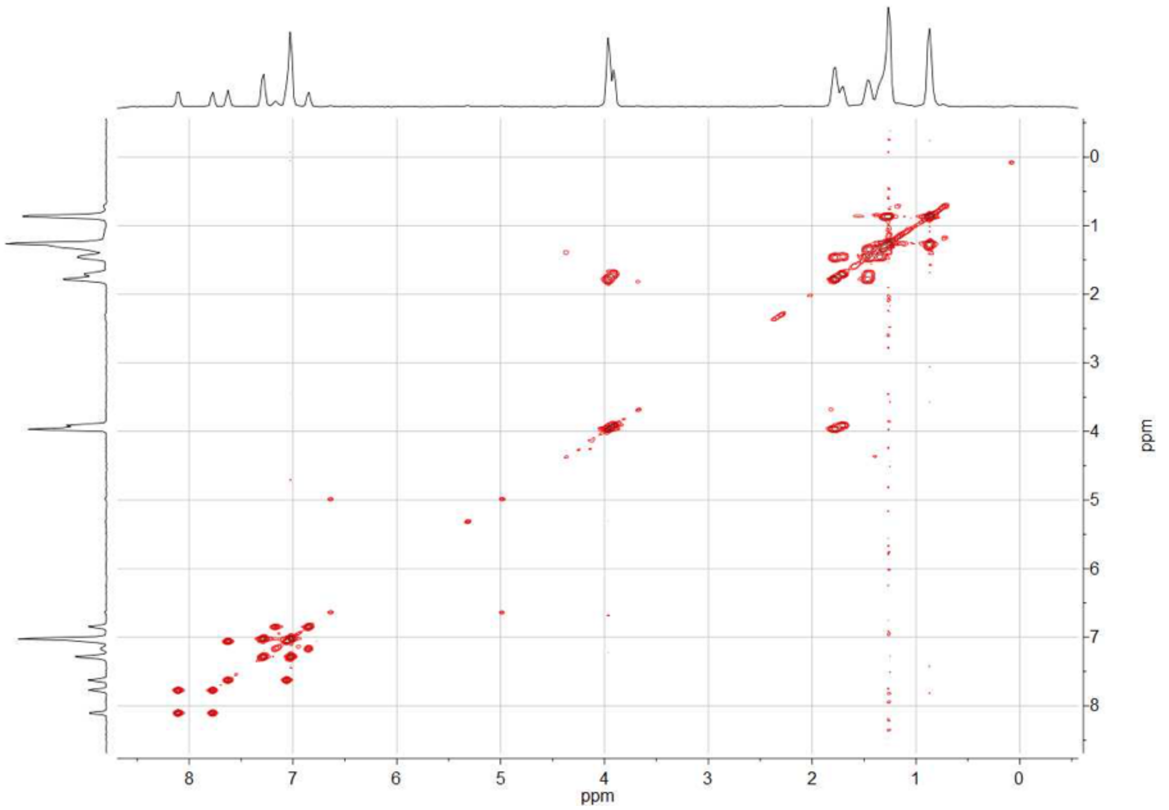

Figure S38: ^{1}H-^{1}H COSY NMR spectrum of **10b** in CD_2Cl_2.

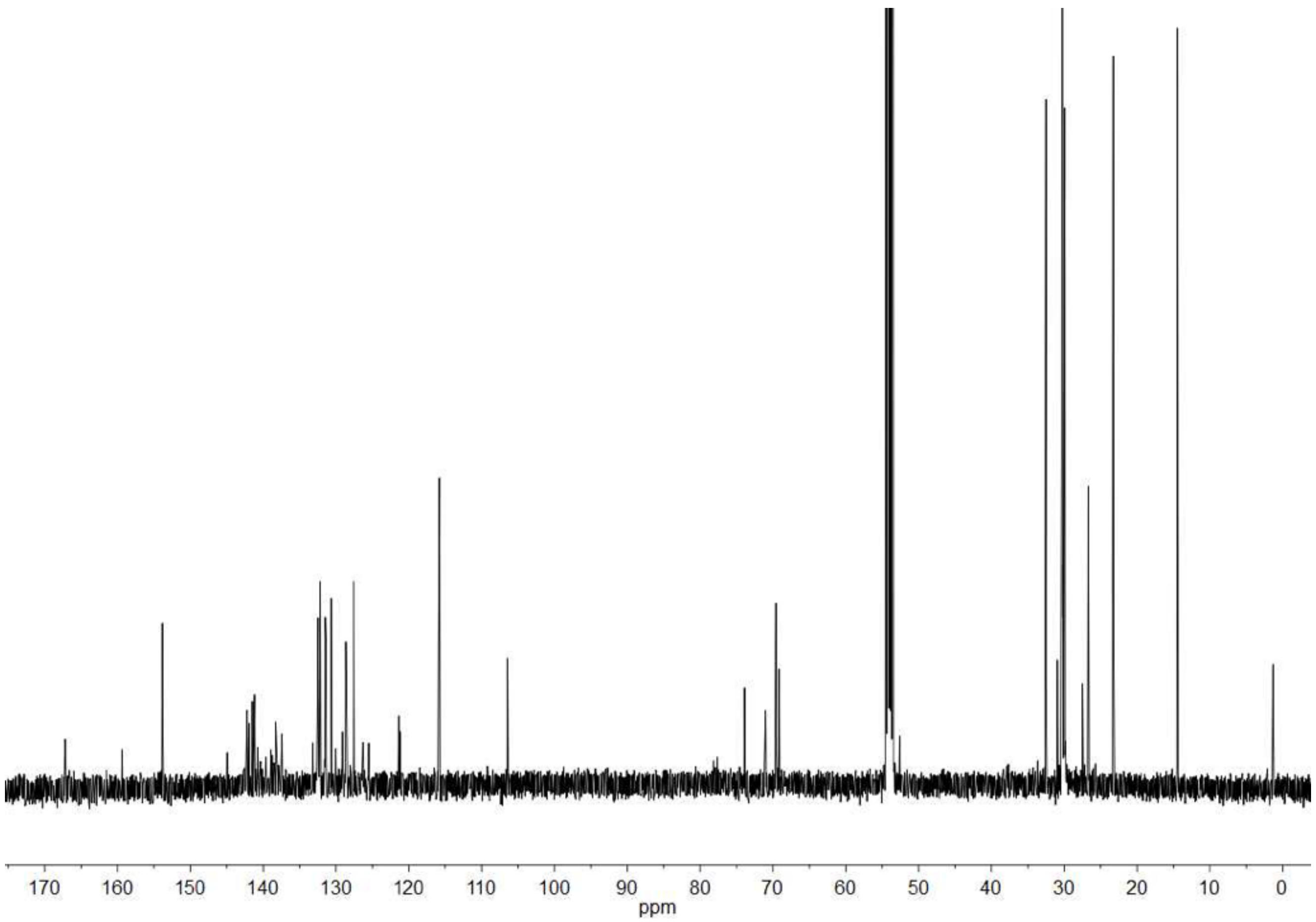

Figure S39: ^{13}C NMR spectrum of **10b** in CD_2Cl_2.

1a

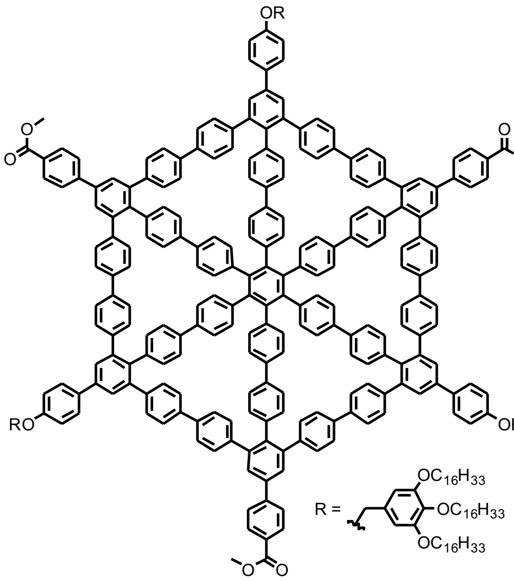

10a (7.2 mg, 1.1 µmol) was dissolved in THF (8 mL). Under Ar atmosphere Ni(COD)$_2$ (12 mg, 43 µmol) and bipy (6.0 mg, 38 µmol) were added in microwave tube in a glove box. The tube was sealed with a septum and *via* a syringe the solution of **10a** in THF and COD (0.2 mL) were added to the tube. The reaction mixture was heated in the microwave to 120 °C for 12 min. Then the solvent was removed under reduced pressure. The crude product was purified by column chromatography (DCM) and recycling GPC. **1a** (5.4 mg, 0.99 µmol, 88 %) was obtained as a colorless solid.

Chemical formula: $C_{393}H_{450}O_{18}$. **^{1}H NMR** (700 MHz, CD_2Cl_2, r.t.): δ [ppm] = 8.12 (d, $^3J_{HH}$ = 8.1 Hz, 6H), 7.83 (d, $^3J_{HH}$ = 8.4 Hz, 6H), 7.77 (s, 6H), 7.70 – 7.65 (m, 12H), 7.49 – 7.44 (m, 24H), 7.22 – 7.15 (m, 36H), 7.13 (d, $^3J_{HH}$ = 7.8 Hz, 12H), 7.08 (d, $^3J_{HH}$ = 8.3 Hz, 6H), 6.95 (d, $^3J_{HH}$ = 7.7 Hz, 12H), 6.86 – 6.79 (m, 12H), 6.65 (s, 6H), 5.00 (s, 6H), 3.97 (t, $^3J_{HH}$ = 6.5 Hz, 12H), 3.94 – 3.90 (m, 15H), 1.83 – 1.75 (m, 12H), 1.74 – 1.67 (m, 6H), 1.50 – 1.42 (m, 18H), 1.38 – 1.21 (m, 216H), 0.91 – 0.85 (m, 27H). **MS** (MALDI-pos, DCTB), *m/z* (%): 5763.1 (80) [3M+($C_{55}H_{103}O_3$ = R)+Na+2K]$^{3+}$, 5643.5 (100) [3M+2DCTB+2Na+H]$^{3+}$, 5513.9 (100) [M+K; Overlapping with the adjacent distribution]$^+$, 3052.2 (25) [M+Na-3×($C_{55}H_{103}O_3$ = R)]$^{3+}$, 2644.4 (67) [2M+DCTB-2×($C_{55}H_{103}O_3$ = R)+3H]$^{3+}$. GPC (PS calibration): M_P = 7880 g mol^{-1}.

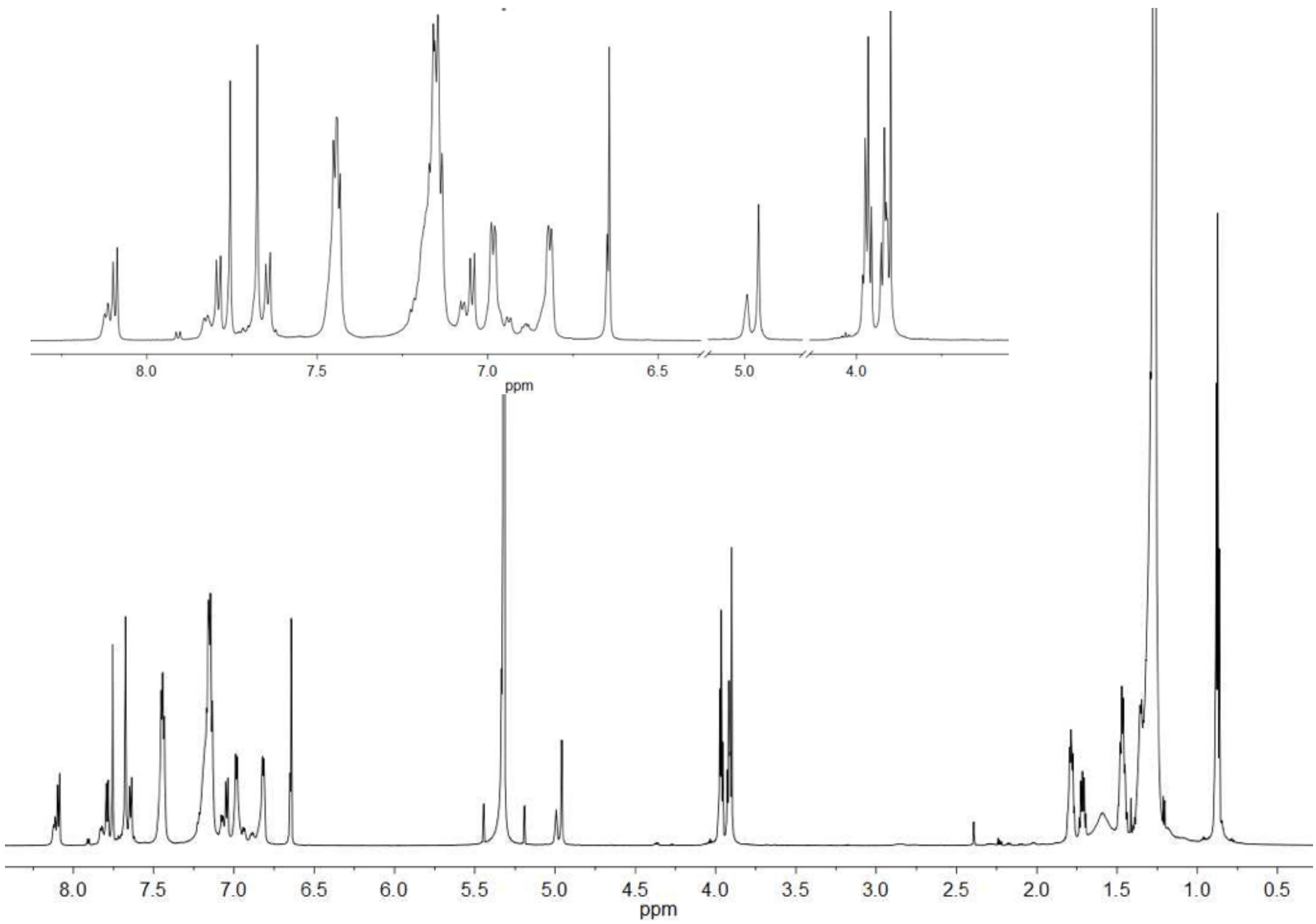

Figure S40: ^{1}H NMR spectrum of **1a** in CD_2Cl_2 (1.57×10^{-3} mol $\times$ L^{-1}).

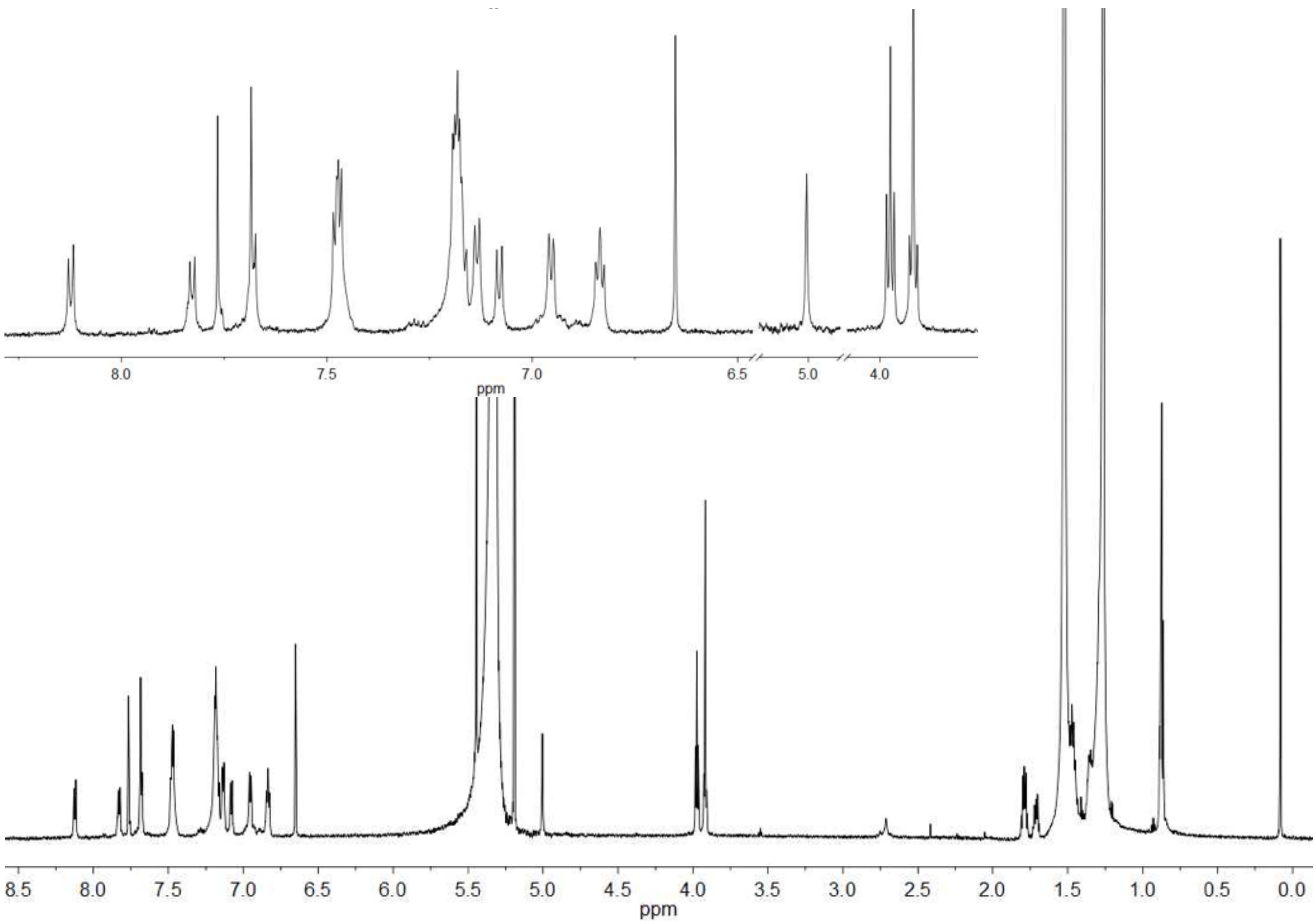

Figure S41: ^{1}H NMR spectrum of **1a** in CD_2Cl_2 (4.92×10^{-5} mol $\times$ L^{-1}).

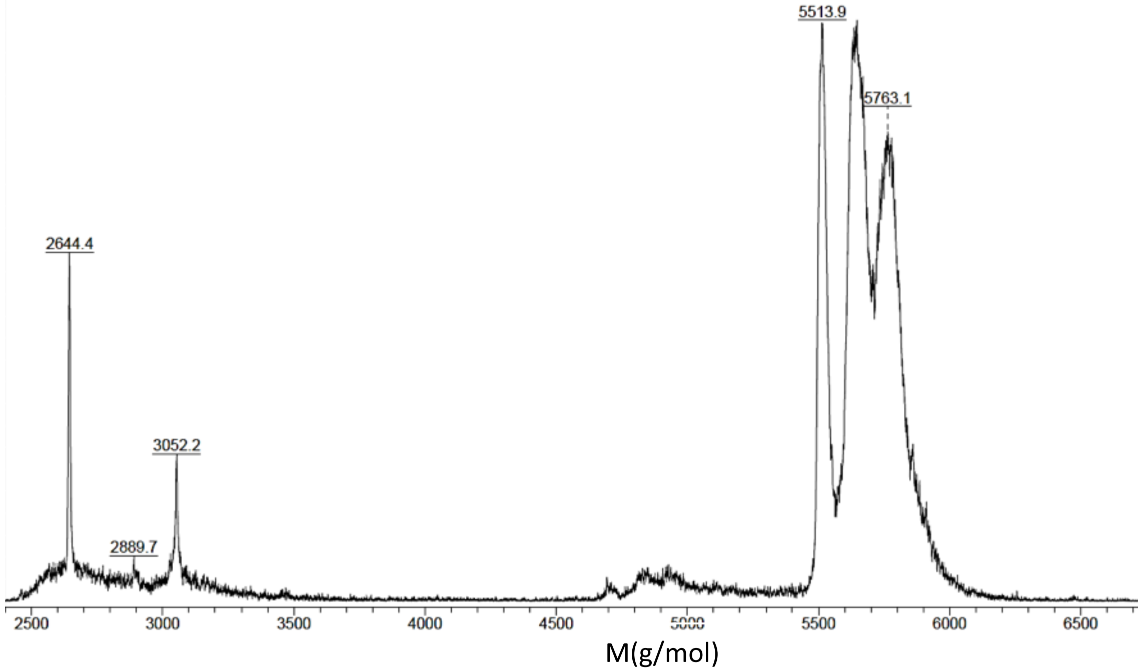

Figure S42: MALDI-TOF mass spectrum of **1a** (matrix: DCTB).

2a

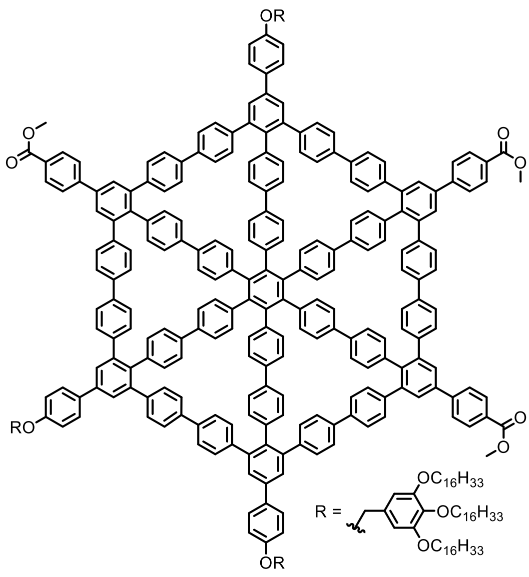

10b (6.8 mg, 1.1 µmol) was dissolved in THF (7.7 mL). Under Ar atmosphere, Ni(COD)$_2$ (14 mg, 49 µmol) and bipy (7.0 mg, 45 µmol) were added in microwave tube in a glove box. The tube was sealed with a septum, and *via* a syringe the solution of **10b** in THF and COD (0.3 mL) were added to the tube. The reaction mixture was heated in the microwave to 120 °C for 12 min. Then the solvent was removed under reduced pressure. The crude product was purified by column chromatography (DCM) and recycling GPC. **2a** (5.2 mg, 0.96 µmol, 90 %) was obtained as a colorless solid.

Chemical formula: $C_{393}H_{450}O_{18}$. **^{1}H NMR** (700 MHz, CD_2Cl_2, r.t.): δ [ppm] = 8.12 (d, $^3J_{HH}$ = 8.1 Hz, 6H), 7.83 (d, $^3J_{HH}$ = 8.4 Hz, 6H), 7.77 (s, 6H), 7.70 – 7.65 (m, 12H), 7.49 – 7.44 (m, 24H), 7.22 – 7.15 (m, 36H), 7.13 (d, $^3J_{HH}$ = 7.8 Hz, 12H), 7.08 (d, $^3J_{HH}$ = 8.3 Hz, 6H), 6.95 (d, $^3J_{HH}$ = 7.7 Hz, 12H), 6.86 – 6.79 (m, 12H), 6.65 (s, 6H), 5.00 (s, 6H), 3.97 (t, $^3J_{HH}$ = 6.5 Hz, 12H), 3.94 – 3.90 (m, 15H), 1.83 – 1.75 (m, 12H), 1.74 – 1.67 (m, 6H), 1.50 – 1.42 (m, 18H), 1.38 – 1.21 (m, 216H), 0.91 – 0.85 (m, 27H). **MS** (MALDI-pos, DCTB), *m/z* (%): 3028.4 (100) [M-3×($C_{55}H_{103}O_3$ = R)]$^+$, 5503.5 (80) [M+K]$^+$, 5607.5 (15) [2M+2Na+DCTB]$^{2+}$. GPC (PS calibration): M_P = 7484 g mol^{-1}.

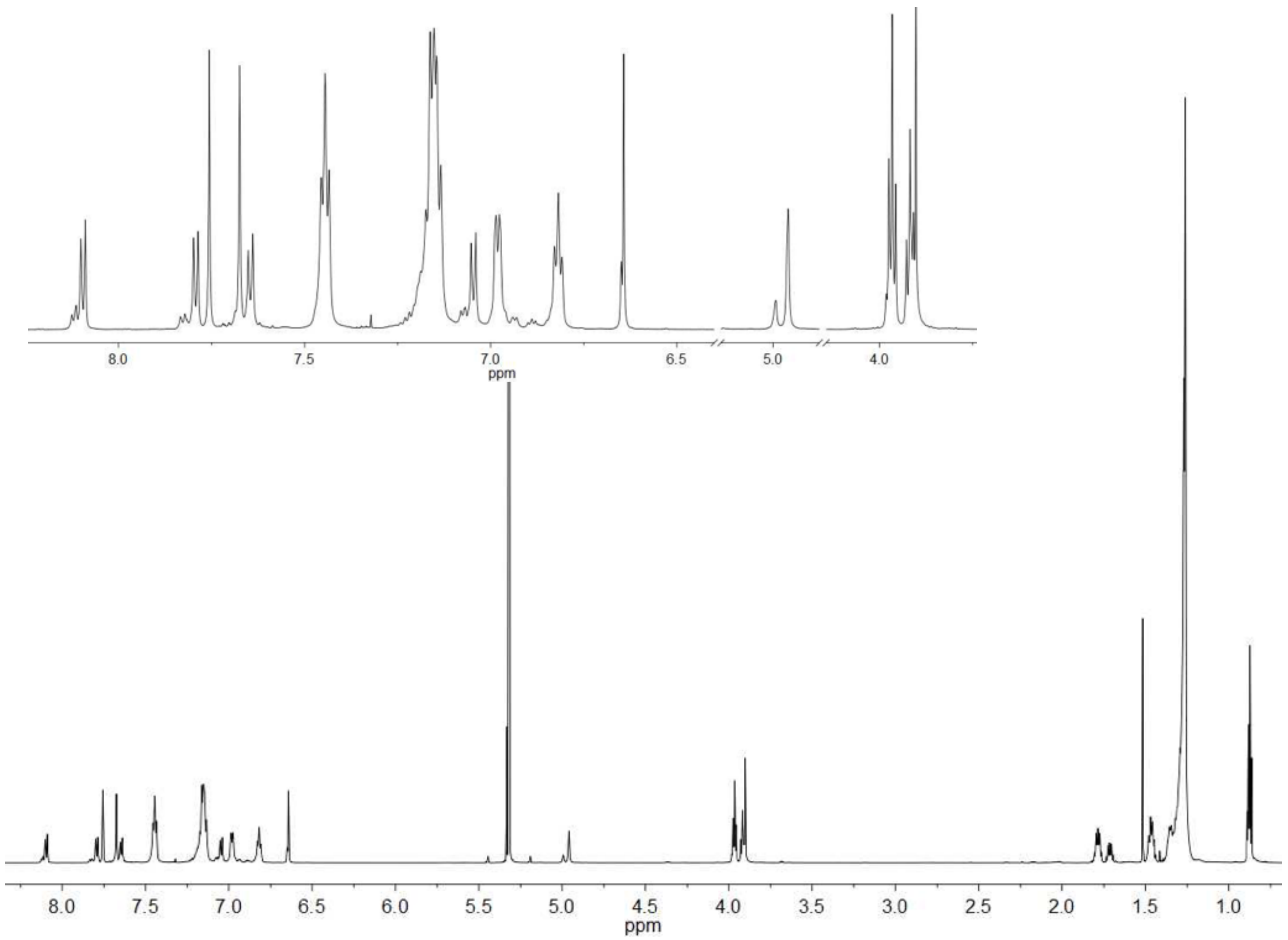

Figure S43: ^{1}H NMR spectrum of **2a** in CD_2Cl_2 (1.57×10^{-3} mol $\times$ L^{-1})

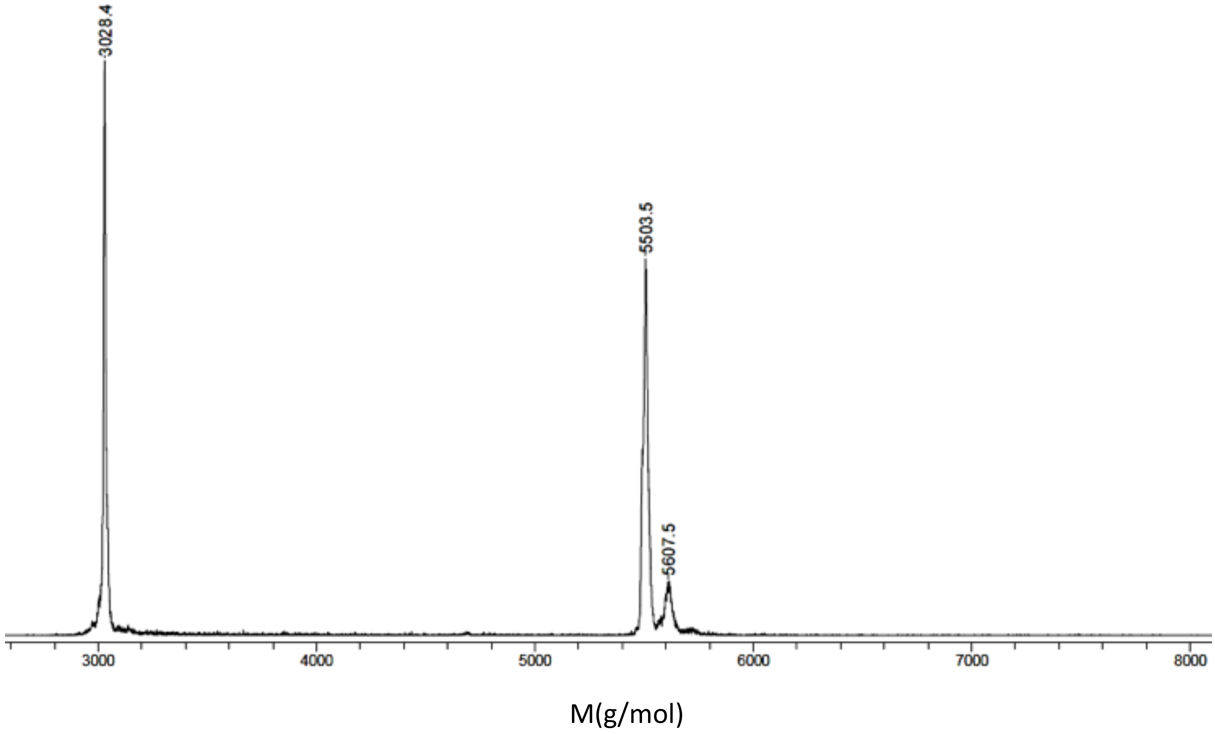

Figure S44: MALDI-TOF mass spectrum of **2a** (matrix: DCTB).

1b

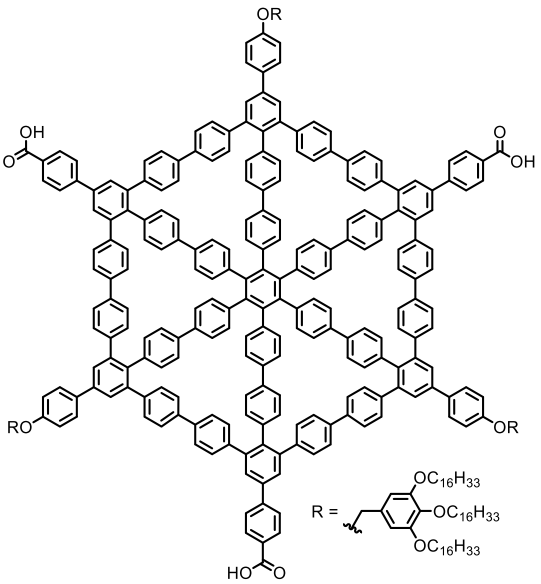

1a (4.0 mg, 0.73 µmol) was dissolved in THF (2 mL). LiOH (5.3 mg, 0.22 mmol) and water (50 µL) were added to the tube. The resulting mixture was stirred for 18 h at 45 °C. The reaction mixture was diluted with HCl (10 %) and DCM. After extraction of the aqueous layer with DCM, the combined organic phases were washed with water and brine and dried over $MgSO_4$. After removal of the solvent, the residue was dissolved in THF (5 mL) and precipitated in acetonitrile (20 mL). The precipitate was filtered off and was washed with acetonitrile. **1b** (3.5 mg, 0.64 µmol, 88 %) was obtained as a white solid.

Chemical formula: $C_{390}H_{444}O_{18}$. **^{1}H NMR** (400 MHz, THF-d_8, r.t.): δ [ppm] = 8.12 (d, $^{3}J_{HH}$ = 8.1 Hz, 6H), 7.90 (d, $^{3}J_{HH}$ = 8.0 Hz, 6H), 7.84 (d, $^{3}J_{HH}$ = 3.2 Hz, 6H), 7.73 – 7.70 (m, 12H), 7.56 – 7.47 (m, 24H), 7.24 – 7.12 (m, 48H), 7.08 (d, $^{3}J_{HH}$ = 9.0 Hz, 6H), 6.94 (d, $^{3}J_{HH}$ = 7.9 Hz, 12H), 6.85 – 6.78 (m, 12H), 6.71 (s, 6H), 5.02 (s, 6H), 3.98 (t, $^{3}J_{HH}$ = 6.3 Hz, 12H), 3.92 (t, $^{3}J_{HH}$ = 6.3 Hz, 6H), 1.81 – 1.73 (m, 18H), 1.58 – 1.46 (m, 18H), 1.41 – 1.23 (m, 216H), 0.93 – 0.85 (m, 27H). **MS** (MALDI-pos, DCTB), *m/z* (%): 6442.5 (11) [M+DCTB+($C_{55}H_{103}O_3$ = R)+H]$^{+}$, 5635.1 (14) [2M+2DCTB-2CO_2+Na+H]$^{2+}$, 5445.8 (M+Na), 4821.8 (7) [2M+2DCTB-2x($C_{55}H_{103}O_3$ = R)-2CO_2+Na+H]$^{2+}$, 3986.5 (28) [3M+2DCTB-6×($C_{55}H_{103}O_3$ = R)+3Na]$^{3+}$, 2986.1 (16) [M-3×($C_{55}H_{103}O_3$ = R)]$^{+}$, 2616.0 (100) [2M+DCTB-2x($C_{55}H_{103}O_3$ = R)+3H]$^{3+}$. GPC (PS calibration): M_P = 7235 g mol^{-1}.

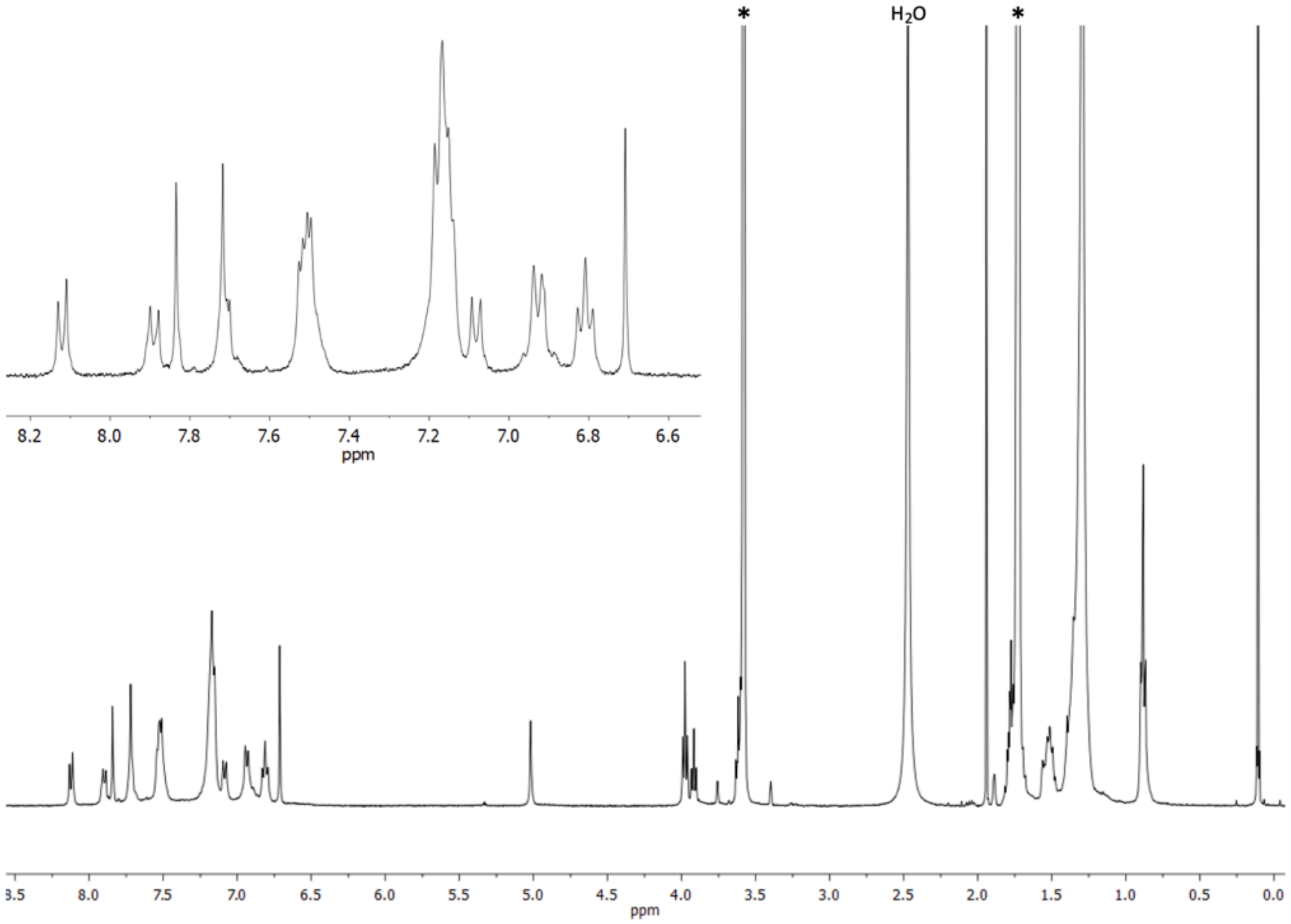

Figure S45: ^{1}H NMR spectrum of **1b** in THF-d_8*.

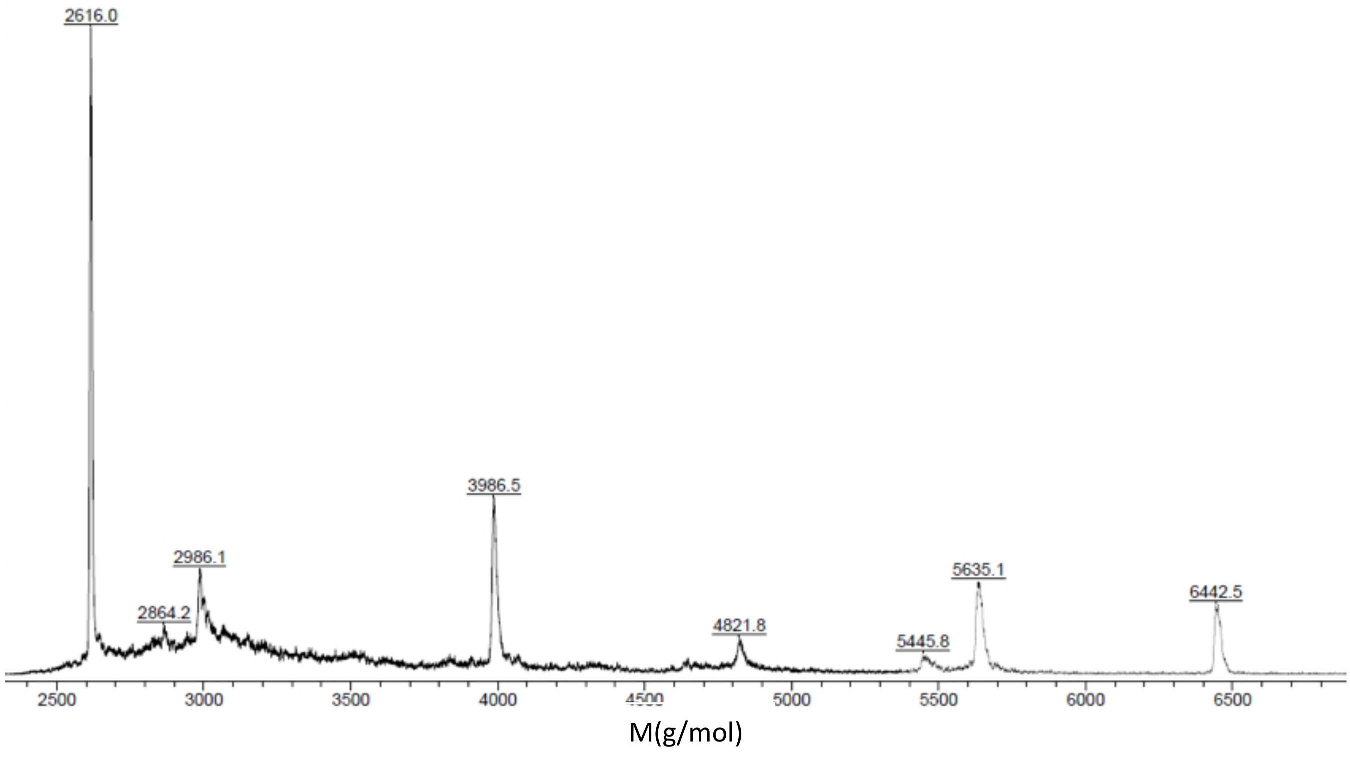

Figure S46: MALDI-TOF mass spectrum of **1b** (matrix: DCTB).

2b

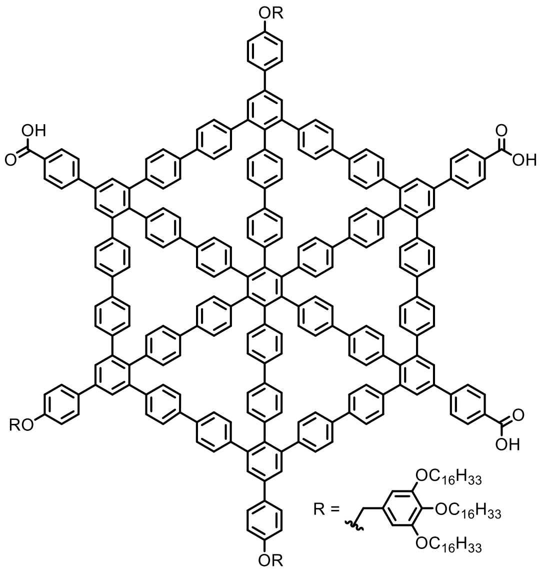

2a (15 mg, 2.7 µmol) was dissolved in THF (3 mL). LiOH (19 mg, 807 µmol) and water (50 µL) were added to the tube. The resulting mixture was stirred for 18 h at 45 °C. The reaction mixture was diluted with HCl (10 %) and DCM. After extraction of the aqueous layer with DCM, the combined organic phases were washed with water and brine and dried over $MgSO_4$. After removal of the solvent, the residue was dissolved in THF (5 mL) and precipitated in acetonitrile (20 mL). The precipitate was filtered off and was washed with acetonitrile. **2b** (12.8 mg, 2.34 µmol, 87 %) was obtained as a white solid.

Chemical formula: $C_{390}H_{444}O_{18}$. **^{1}H NMR** (400 MHz, THF-d_8, r.t.): δ [ppm] = 8.12 (d, $^3J_{HH}$ = 8.1 Hz, 6H), 7.90 (d, $^3J_{HH}$ = 8.0 Hz, 6H), 7.84 (d, $^3J_{HH}$ = 3.2 Hz, 6H), 7.73 – 7.70 (m, 12H), 7.56 – 7.47 (m, 24H), 7.24 – 7.12 (m, 48H), 7.08 (d, $^3J_{HH}$ = 9.0 Hz, 6H), 6.94 (d, $^3J_{HH}$ = 7.9 Hz, 12H), 6.85 – 6.78 (m, 12H), 6.71 (s, 6H), 5.02 (s, 6H), 3.98 (t, $^3J_{HH}$ = 6.3 Hz, 12H), 3.92 (t, $^3J_{HH}$ = 6.3 Hz, 6H), 1.81 – 1.73 (m, 18H), 1.58 – 1.46 (m, 18H), 1.41 – 1.23 (m, 216H), 0.93 – 0.85 (m, 27H). **MS** (MALDI-pos, DCTB), *m/z* (%): 3092.6 (32) [M-3×($C_{55}H_{103}O_3$ = R)+Ag]$^+$, 2986.0 (100) [M-3×($C_{55}H_{103}O_3$ = R)]$^+$, 2941.7 (55) [M-3x($C_{55}H_{103}O_3$ = R)-CO_2-H]$^+$, 2957.1 (55) [M-3x($C_{55}H_{103}O_3$ = R)- 2×(OH)]$^+$, 2970.5 (46) [M-3×($C_{55}H_{103}O_3$ = R)-OH]$^+$, 2895.1 (29) [M-3×($C_{55}H_{103}O_3$ = R)-2×(CO_2-H)]$^+$. GPC (PS calibration): M_P = 7906 g mol^{-1}.

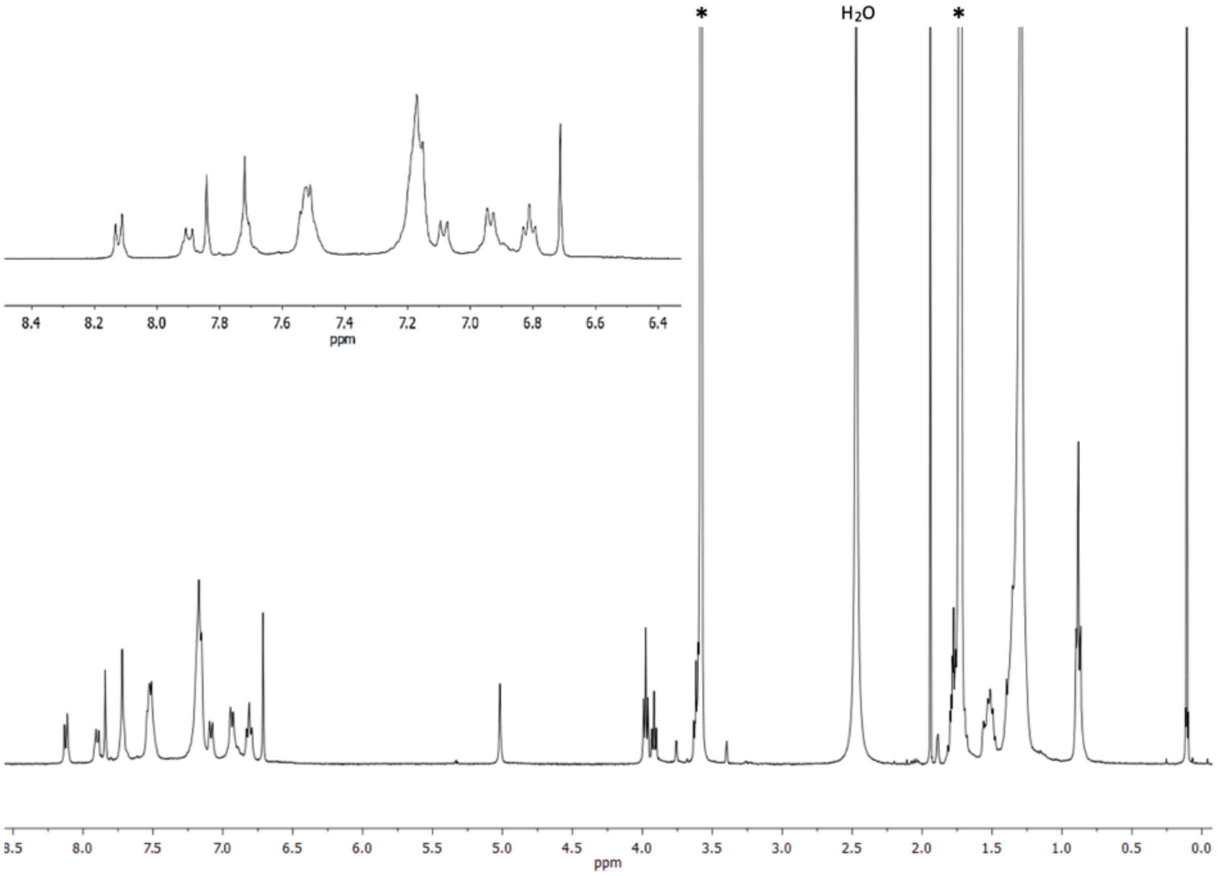

Figure S47: ^{1}H NMR spectrum of **2b** in THF-d_8*.

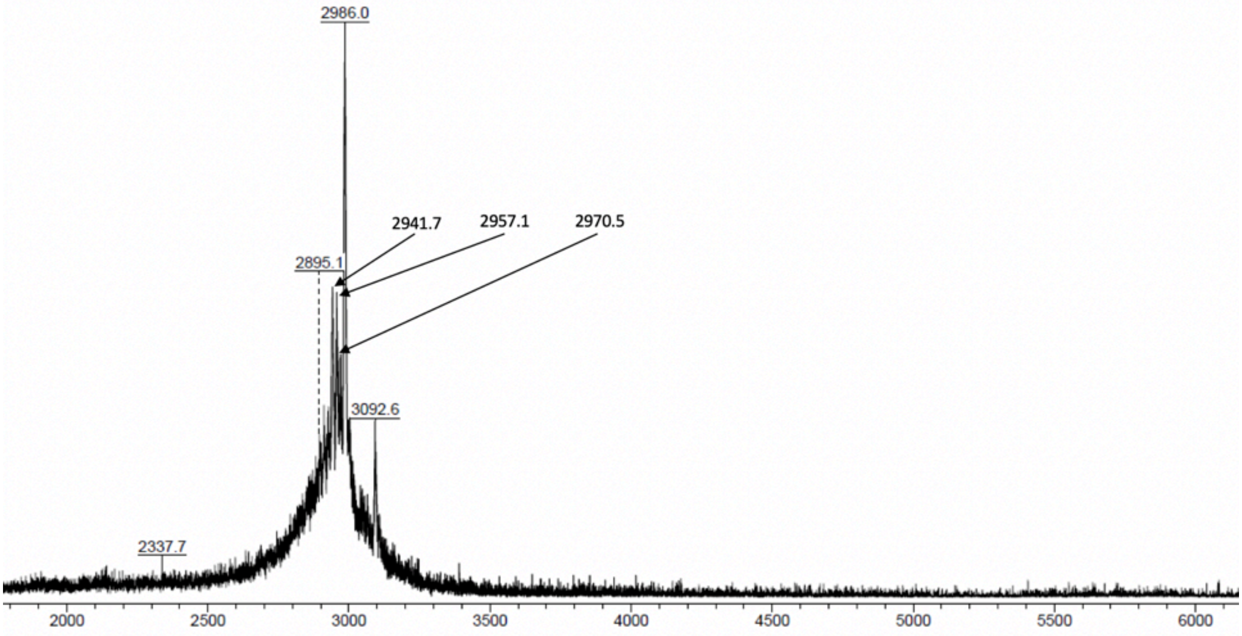

Figure S48: MALDI-TOF mass spectrum of **2b** (matrix: DCTB).

4.3 Melting

The methyl esters **1a**/**2a** as well as the free acids **1b**/**2b** do not melt up to a temperature of 210 °C, which is a sharp contrast to phenylene wheels with six trihexadecyloxybenzyl groups.[S6]

5 Aggregation of 1a

While the methyl esters **1a**/**2a** dissolve well in common organic solvents under ambient conditions, the acids **1b**/**2b** dissolve well only in THF. Solutions of the methyl esters MSWs **1a**/**1b** in THF show (in the ^{1}H NMR spectrum) sharp signals, and the positions of the signals as well as their intensities coincide with their structures (see Fig. S49). In DCM, a concentration dependence of the spectra of **1a** is observed (see Figure S49) that indicates a fast monomer-dimer equilibrium accompanied with a slow monomer/dimer-oligomer equilibrium. At low concentrations ($< 2 \times 10^{-5}$ M), the benzyl protons of the oligoalkoxy side groups, for example, show a sharp signal that shifts upfield when the concentration of the wheel is increased. In addition, a second, broader singlet close nearby starts to appear and changes its position only slightly with increasing concentration. At present, we interpret this behavior as a signature of a fast monomer-dimer equilibrium which leads to a concentration-dependent sharp NMR signal at any concentration. With increasing concentration, higher aggregates are formed. These stand in slow exchange with the monomer and dimer relative to the timescale of NMR, so that a second set of signals appears in the NMR spectrum.

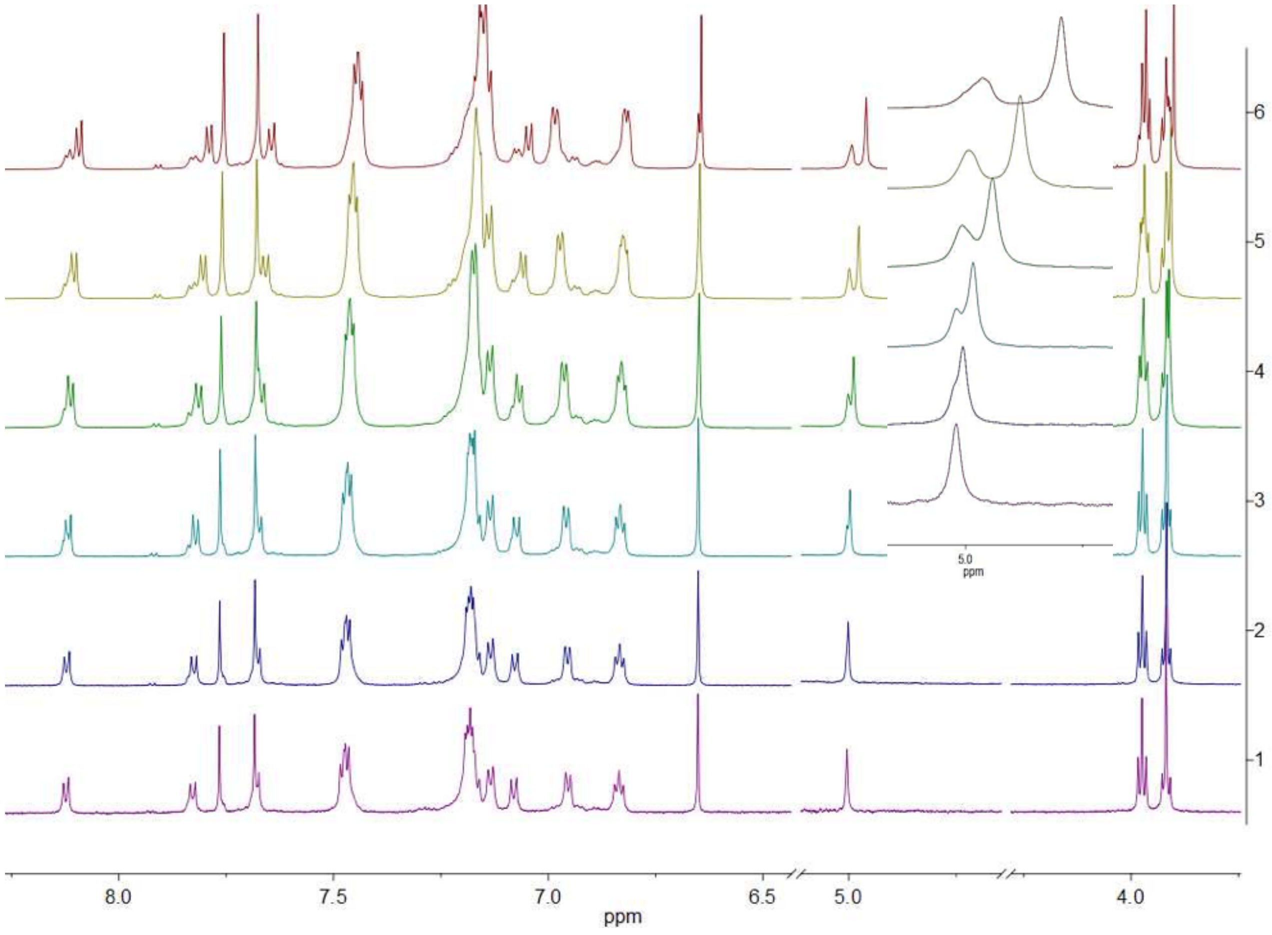

Figure S49: ^{1}H NMR spectrum of **1a** in CD_2Cl_2 at different concentrations (6: 1.57×10^{-3} M, 5: 7.86×10^{-4} M, 4: 3.93×10^{-4} M, 3: 1.97×10^{-4} M, 2: 9.83×10^{-5} M, 1: 4.92×10^{-5} M).

Scheme S2: Proposed monomer-dimer-polymer equilibrium in DCM (schematically).

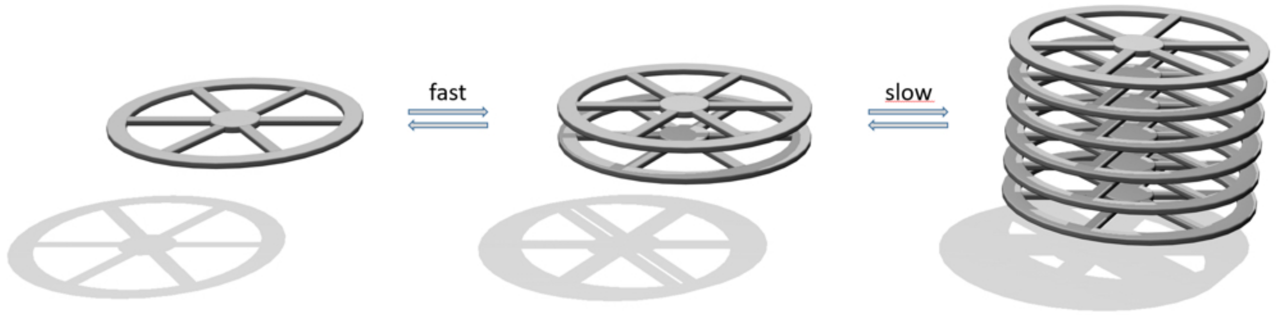

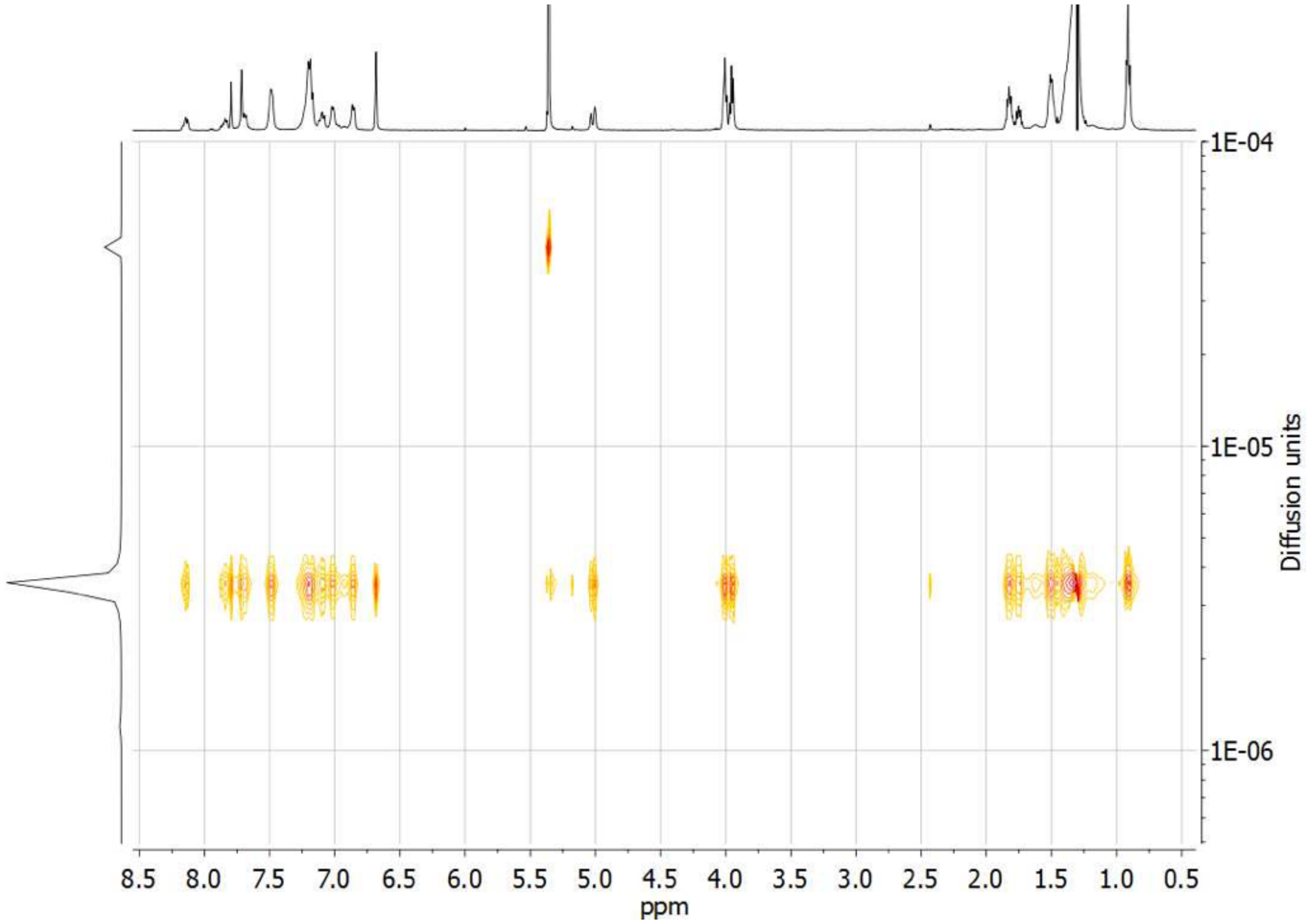

Figure S50: ^{1}H-^{1}H DOSY NMR spectrum of **1a** in CD_2Cl_2 (1.57×10^{-3} mol $\times$ L^{-1}).

In the DOSY NMR experiment no different diffusion units could be detected, thus making DOSY measurements inadequate for the determination of the aggregated species.

6 Photophysical properties

6.1 UV/vis absorption spectra

UV/vis absorption spectra of **1a** (Fig. S51) exhibit a longest wavelength maximum at 300 nm, similar to the longest wavelength absorption of *p*-quaterphenyl.

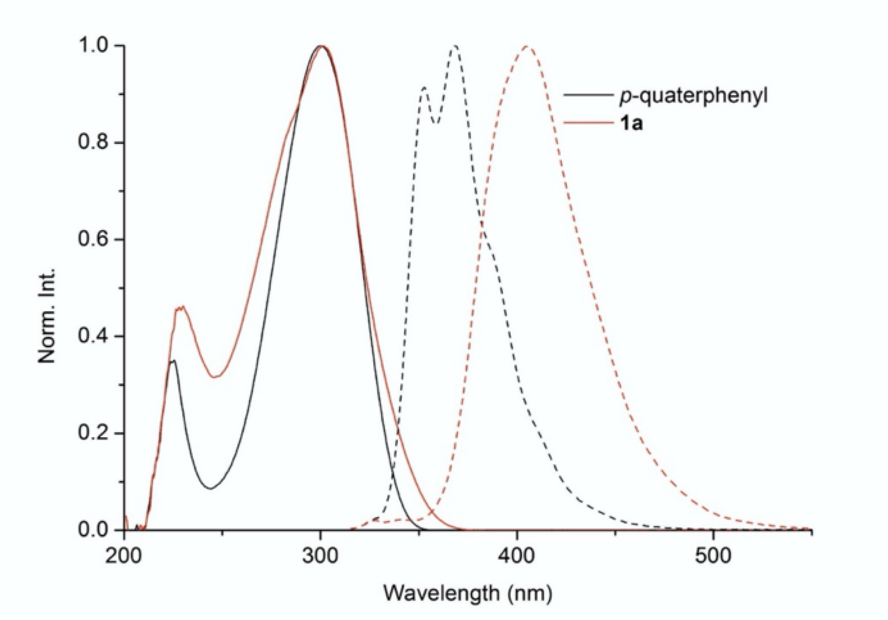

Figure S51: Normalized UV/vis spectra (solid) and fluorescence (F) spectra (dashed) of **1a** (maxima at UV/vis: 301.5 nm, F: 406.0 nm) and *p*-quaterphenyl (maxima at UV/vis: 300.0 nm, F: 368.5 nm) in THF at r.t. in air.

6.2 Photoluminescence lifetime decay of **1a** and *p*-quaterphenyl

The conclusions on aggregation of the compounds (cf. chapter 6) are consistent with the observation of aggregate luminescence in solution (even at low concentrations). The fluorescence of **1a** in toluene solution is found to be shifted by 40 nm to the red compared to *p*-quaterphenyl. In addition, the spectrum appears broadened and featureless, with the characteristic vibronic progression suppressed. The fluorescence lifetime increases in **1a** to 1.5 ns from 0.9 ns for *p*-quaterphenyl (Figure S52). These changes in spectral properties of the emitting chromophore are reminiscent of aggregation effects and the aggregation-induced emission of *p*-quaterphenyl.[S8]

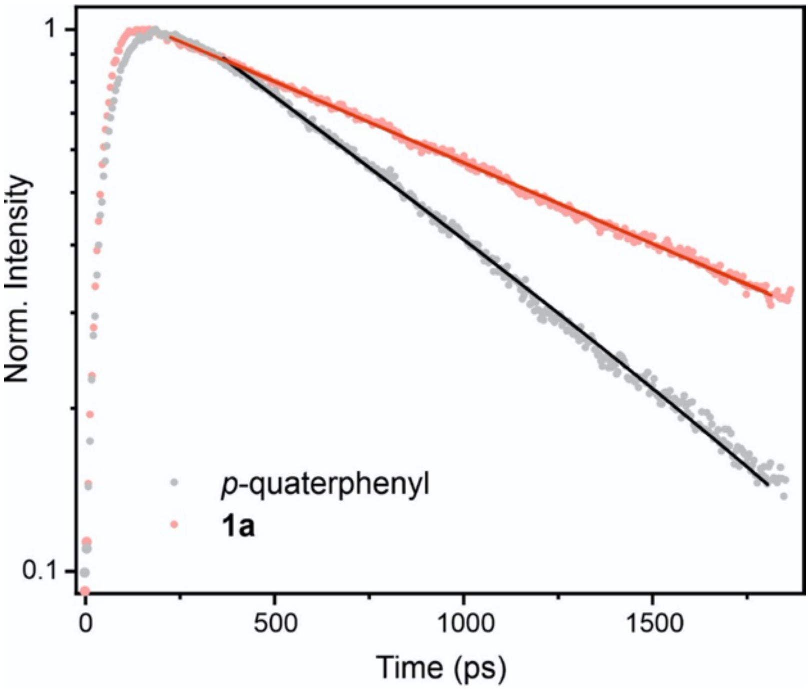

Figure S52: Photoluminescence lifetime decays of dilute toluene toluene solutions of **1a** and *p*-quaterphenyl, measured using a streak camera and excited at 300 nm by a frequency-tripled femtosecond laser at 80 MHz repetition rate. A single-exponential decay (solid lines) fits the kinetics, yielding the PL lifetime for **1a** (red) as $\tau_{PL} = 1.48 \pm 0.02$ ns and for *p*-quaterphenyl (black) as $\tau_{PL} = 0.86 \pm 0.01$ ns. The increase of the PL lifetime in **1a** can be assigned to aggregation effects due to π-π stacking of the *p*-quaterphenyl chromophores of the MSW. Restrictions of the rotation of phenyl units in such aggregates can give rise to aggregation induced emission as reported recently.[S9]

6.3 Streak camera images of **1a** and *p*-quaterphenyl

We examined the fluorescence by picosecond time-resolved detection using a streak camera, but were not able to resolve emission of the individual *p*-quaterphenyl chromophores in **1a** (see Figure S53), suggesting that dimerization of the MSW is prevalent, even in dilute solution.

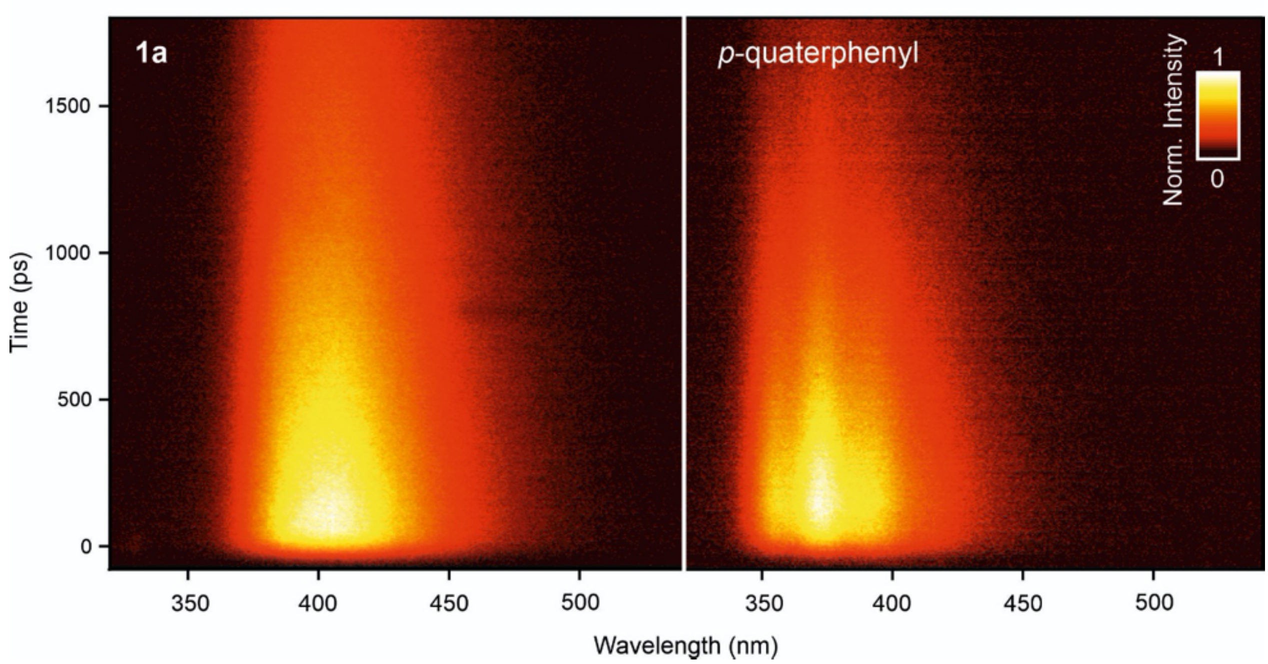

Figure S53: Streak-camera images of dilute toluene solutions of **1a** and *p*-quaterphenyl, excited at 300 nm by a frequency-tripled femtosecond laser at 80 MHz repetition rate. The normalized PL intensity is plotted versus wavelength and time. This measurement shows that there are no dynamic spectral effects in the photoluminescence. Such effects could conceivably arise due to energy transfer or structural relaxation. However, all emission of **1a** comes from the aggregate state of *p*-quaterphenyl and there are no spectral features of the isolated *p*-quaterphenyl chromophore detectable, suggesting that aggregation induced emission is prevalent even in dilute solution.

7 References

[S1] Keller, T. J.; Bahr, J.; Gratzfeld, K.; Schönfelder, N.; Majewski, M. A.; Stępień, M.; Höger, S.; Jester, S.-S. *Beilstein J. Org. Chem.* **2019**, *15*, 1848–1855.

[S2] Bannwarth, C.; Ehlert, S.; Grimme, S. *J. Comput. Theory Chem.* **2019**, *15*, 1652–1671.

[S3] Semiempirical extended tight-binding program package xtb, version 6.1, https://github.com/grimme-lab/xtb. Accessed: 2020-04-29.

[S4] Spicher, S.; Grimme S. *Angew. Chem. Int. Ed.* **2020**, *59*, 15665–15673.

[S5] Coutsias, E. A.; Seok, C.; Dill, K. A. *J. Comput. Chem.* **2004**, *25*, 1849–1857.

[S6] Zimmermann, T.; Fischer, G. W. *J. Prakt. Chem.* **1987**, *329*, 975–984.

[S7] Idelson, A.; Sterzenbach, C.; Jester, S.-S.; Tschierske, C.; Baumeister, U.; Höger, S. *J. Am. Chem. Soc.* **2017**, *139*, 4429–4434.

[S8] Eder, T.; Stangl, T.; Gmelch, M.; Remmerssen, K.; Laux, D.; Höger, S.; Lupton, J. M.; Vogelsang, J. *Nat. Commun.* **2017**, 8, 1641.

[S9] Sun, Y.; Wang, Y.; Wu, M.; Yuan, W.; Chen, Y. *Chem. Asian J.* **2017**, 12, 52–59.
